# Coronary artery disease patient-derived iPSC-hepatocytes have distinct miRNA profile that may alter lipid metabolism

**DOI:** 10.1038/s41598-023-28981-7

**Published:** 2023-01-30

**Authors:** Anna Alexanova, Emma Raitoharju, Joona Valtonen, Katriina Aalto-Setälä, Leena E. Viiri

**Affiliations:** 1 The Cardiovascular Research Center Tampere, Faculty of Medicine and Health Technology, Tampere University, Arvo Ylpön Katu 34, 33520 Tampere, Finland; 2 Molecular Epidemiology, Faculty of Medicine and Health Technology, Tampere University, Tampere, Finland; 3 Tampere University Hospital, Tampere, Finland

**Keywords:** Stem-cell differentiation, Cardiovascular diseases

## Abstract

Metabolic dysfunction, partly driven by altered liver function, predisposes to coronary artery disease (CAD), but the role of liver in vulnerable atherosclerotic plaque development remains unclear. Here we produced hepatocyte-like cells (HLCs) from 27 induced pluripotent stem cell (iPSC) lines derived from 15 study subjects with stable CAD (n = 5), acute CAD (n = 5) or healthy controls (n = 5). We performed a miRNA microarray screening throughout the differentiation, as well as compared iPSC-HLCs miRNA profiles of the patient groups to identify miRNAs involved in the development of CAD. MicroRNA profile changed during differentiation and started to resemble that of the primary human hepatocytes. In the microarray, 35 and 87 miRNAs were statistically significantly deregulated in the acute and stable CAD patients, respectively, compared to controls. Down-regulation of miR-149-5p, -92a-3p and -221-3p, and up-regulation of miR-122-5p was verified in the stable CAD patients when compared to other groups. The predicted targets of deregulated miRNAs were enriched in pathways connected to insulin signalling, inflammation and lipid metabolism. The iPSC-HLCs derived from stable CAD patients with extensive lesions had a distinct genetic miRNA profile possibly linked to metabolic dysfunction, potentially explaining the susceptibility to developing CAD. The iPSC-HLCs from acute CAD patients with only the acute rupture in otherwise healthy coronaries did not present a distinct miRNA profile, suggesting that hepatic miRNAs do not explain susceptibility to plaque rupture.

## Introduction

Coronary artery disease (CAD) is the leading cause of death worldwide, however, not all CAD patients are at an equal mortality risk. Patients with stable CAD have atherosclerotic plaques with a thick fibrous cap, which are not prone to rupture and thrombosis that could fully occlude the artery and cause myocardial infarction (MI). Atherosclerotic plaques of acute CAD patients, however, are prone to rupture and thus result in higher MI and mortality risk. Increased risk of cardiovascular events like MI and sudden cardiac death are linked to cardiovascular risk factors including abdominal obesity, hypertension, hyperglycemia and dyslipidemia (high triglyceride, low HDL-cholesterol), clustering of which characterizes metabolic syndrome^1,2^. The liver plays a central role in lipid metabolism through participating in the production and clearance of lipoprotein particles, as well as metabolism of triglycerides and cholesterol. While the pathomechanisms leading to diverse CAD entities remain unclear, it is evident that there is an intricate link between hepatic metabolic dysfunction and development of CAD. Thus, an in vitro hepatocyte model offers a unique setting to study liver-related factors involved in lipid/lipoprotein metabolism and atherogenesis contributing to the development of different CAD phenotypes^3^.

MicroRNAs (miRNAs) are short non-coding RNA molecules with a typical length of 22 nucleotides that can bind to mRNA with a complementary sequence, which usually leads to post-transcriptional downregulation of the corresponding gene^4^. In the liver, miRNAs are known to regulate lipoprotein and cholesterol metabolism by inhibiting the expression of cholesterol transporters, LDL receptor and scavenger receptor BI (SR-BI). Therefore, miRNAs are involved in the atherosclerotic process underlying CAD through regulation of cholesterol homeostasis, but also through modulating endothelial dysfunction, inflammation and neoangiogenesis^5^. In addition, miRNAs play a significant role in lipid metabolism beyond cholesterol, and also in insulin signalling in the liver, thus contributing to the pathways that are often dysregulated in metabolic disorders, such as non-alcoholic fatty liver disease (NAFLD) and type 2 diabetes mellitus (T2DM), which are closely linked to CAD^6^. MicroRNAs have been proposed as regulators and biomarkers of CAD pathogenesis but only few of these have been shown in several studies to be consistently up- or down-regulated in stable or acute CAD^7^. Variable results in miRNA studies are most probably due to factors like differences in miRNA detection methods, variable sample source as well as variable CAD criteria. Furthermore, the tissue origin of the circulating miRNAs often remains unclear. It has been shown that the liver has a very specific miRNA profile, and liver-derived miRNA levels in the circulation change as metabolic dysfunction develops. These circulating miRNAs can then facilitate communication with other organs, and it has been proposed that changes in miRNA expression in the liver can affect vascular health^8^.

Identifying CAD patients with increased plaque rupture and mortality risk is an ongoing challenge, which is not fully answered by conventional risk factors such as elevated LDL cholesterol^9^. Several attempts have been made to discover novel biomarkers for CAD namely by analysis of plasma from large cohorts, and certain molecular lipids, such as ceramides, have previously been linked to increased cardiovascular mortality^10^. We wanted to study genetic alterations in miRNA expression that could be attributed to CAD process. We utilized induced pluripotent stem cell (iPSC) technology and hepatocyte differentiation to produce patient-specific iPSC hepatocyte-like cells (iPSC-HLCs) from acute and stable CAD patients as well as healthy individuals. The iPSC-HLCs have previously been shown to closely mimic gene expression and lipidomic profile of primary human hepatocytes (PHHs)^11^, and here we aimed to study whether the miRNA profiles of iPSC-HLCs would differ between the CAD patient groups, and reflect their phenotype.

## Materials and methods

### Study subjects and ethical approval

Written and informed consent was obtained from all study subjects. The Ethics Committee of Tampere University Hospital has approved the study and patient recruitment (Approval Number: R12123) and all experiments were performed in accordance with relevant guidelines and regulations. Altogether 15 study subjects were recruited and classified based on their clinical history and angiography findings. Five patients that had experienced an acute MI were classified as the acute CAD group. Based on coronary angiography, they had a single location with acute rupture and otherwise the coronaries appeared completely healthy. Five patients that had severe coronary artery stenosis in several arteries but no history of MI were classified in the stable group. The five study subjects assigned to the control group were individuals with normal angiography. Only two study subjects (patients 108, 137) had T2DM, both diagnosed only recently, years after the skin biopsy was taken, and none of the subjects had familial hypercholesterolemia. Most of the CAD patients had family history for CAD, i.e. a relative, who had either suffered an acute MI or undergone coronary artery bypass graft surgery or percutaneous coronary intervention by the age of 55 or 65, for male and female respectively (Table S1).

### iPSC reprogramming, cell culture and characterization

Skin fibroblasts derived from the patients’ skin biopsies were cultured and reprogrammed into iPSCs with Sendai reprogramming kit or plasmids as described before^12^. For each patient, two cell lines arising from different colonies of the reprogrammed cells were generated, altogether 30 cell lines (Table S1). iPSCs were cultured with mouse embryonic fibroblasts (26,000 cells/cm^2^; CellSystems Biotechnologie Vertrieb Gmbh) and characterized for pluripotency by PCR and immunostaining as described before^11–14^.

### Hepatic differentiation

Before hepatic differentiation, the iPSCs were cultured in feeder-free conditions for 2–3 passages on culture plates coated with Geltrex (1:100 dilution) and mTeSR-1 medium (Stemcell Technologies). The iPSCs were differentiated to HLCs using the protocol described by Kajiwara et al. 2012^15^ that was slightly modified. iPSCs were dissociated with 5 min Versene (Gibco) treatment at 37 °C and re-suspended in RPMI 1640 + GlutaMAX (Gibco) supplemented with 1 × B27 (Gibco), 100 ng/ml Activin A (Peprotech, Cat No. 120-14E), 75 ng/ml Wnt3 (R&D systems), 50 U/ml penicillin/streptomycin and 10 µM Rock inhibitor. Cells were seeded with 5–10 × 10^4^ /cm^2^ density. Next 4–12 days cells were cultured in the medium where Rock inhibitor was replaced with 0.5 µM NaB (Sigma, B5887) until cells obtained definitive endoderm (DE) specific morphology. Cells were then transferred to KO-DMEM with 20% KO-SR, 2 mM GlutaMAX, 0.1 mM 2-mercaptoethanol, 1% NEAA, 50 U/ml penicillin/streptomycin and 1% DMSO (Sigma, D2650) and cultured for 4–7 days. Finally, cells were cultured additional 5–11 days in the medium prepared with HCM Hepatocyte BulletKit (Lonza, CC-3198) and supplemented with 25 ng/ml HGF (PHG0254, Thermo Scientific) and 20 ng/ml Oncostatin M (OSM, 295-OM, R&D systems).

### Primary human hepatocytes and HepG2 cells

Primary human hepatocytes (PHH) from two donors Hu8132 (Caucasian 57-year-old female) and HU8210 (Caucasian 51-year-old male) were purchased from Gibco and cultured as described before^16^. Hepatocellular carcinoma cells (HepG2) were acquired from ATCC (HepG2, ATCC‐HB‐8065, Lot. No. 59947519) and cultured in RPMI 1640 + GlutaMAX (Gibco) with 10% fetal bovine serum (FBS, Biosera) and 50 U/ml penicillin/streptomycin (Lonza).

### Characterization of iPSC-derived DE and HLCs

To confirm efficient DE and hepatic differentiation as well as functionality of iPSC-HLCs, the cells were characterized by flow cytometry, LDL uptake, ELISA assays, immunocytochemistry and RT-qPCR as follows.

#### Flow cytometry

To ensure efficient DE differentiation a CXCR4 FACS was performed. For each cell line, cells from three wells were detached with gentle cell dissociation reagent (StemCell) at 37 °C for 5 min and washed with 5% FBS in PBS. Each replicate was stained with CXCR4 antibody (R&D Systems, Minneapolis, USA) for 15 min at room temperature. A mix of the replicates was used as an unstained blank control. Cells were then washed three times with 5% FBS in PBS and analysed with BD Accuri C6 cytometer.

#### Low density lipoprotein uptake

HLCs were tested for ability to uptake low density lipoprotein (LDL) with either LDL conjugated to DyLight™ 550 (Cayman Chemicals, USA, Cat. No. 10011125) or Bodipy FL LDL complex (ThermoFisher Scientific, Cat. No. L3483). Cells were incubated at 37 °C for 3.5 h with labelled LDL that was added to the cell culture medium at 1:100 dilution. HLCs tested with DyLight™ LDL were fixed with 4% PFA at room temperature for 15 min, mounted with Vectashield containing DAPI (Vector Laboratories) and visualized with Olympus IX51 phase-contrast microscope equipped with fluorescence optics and an Olympus DP30BW camera (Olympus Corporation, Hamburg, Germany). HLCs tested with Bodipy LDL were stained with NucBlue (Applied Biosystems) for 30 min at the end of the incubation. They were then fixed with 4% PFA at room temperature for 15 min and mounded with ProLong Gold (Thermo Fisher) followed by visualization with Olympus IX51 microscope as above.

#### Albumin, triglyceride and urea secretion assays

The conditioned medium was collected from HLCs on the last day of culture after 24-h incubation and was used for albumin, triacylglyceride and urea secretion assays. Total protein content from cell lysate was used for normalization. Cells were lysed and proteins extracted with M-PER mammalian protein extraction reagent (ThermoFisher Scientific) and the protein content was measured with Bradford reagent (Thermo Fisher). Albumin secretion assay was performed with Human Albumin ELISA Quantitation set (Bethyl Laboratories, USA), urea secretion was assessed with QuantiChrom™ Urea Assay Kit (BioAssay Systems, USA), and triglyceride secretion with Triglyceride Quantification Kit (BioVision Inc., Cat. No. K622‐100, USA) according to the manufacturers’ instructions.

#### Immunocytochemistry

The HLCs were stained for hepatic markers (AFP, ALB, ASGPR1 and LDLR) and analysed as described before^12^. Secondary antibody only controls were performed for every ICC batch (data not shown). The antibodies used in this study are presented in Table S2.

#### RNA sample collection, RNA extraction and real-time quantitative PCR (RT-qPCR)

RNA samples were collected just before starting the differentiation (iPSCs), at DE stage before moving to the second differentiation stage and at the end of the final stage (HLCs). Three biological replicates (i.e. cell plate wells) were collected at each time point. Hu8210 and Hu2010 PHHs were collected immediately after plating (d0). Total cell RNA was extracted with miRNeasy Mini Kit according to manufacturer’s instructions (Qiagen).

Protein-coding gene RT-qPCR (*OCT4*, *SOX17*, *FOXA2*, *AFP*, *ALB*) was performed to DE, HLC, four randomly chosen iPSC samples (10,100.EURCAs, 11,304.EURCCs, 11,104.EURCAs, 13,701.EURCSs), HepG2 cells, PHHs and Human Liver Total RNA (TLR, Ambion). cDNA was generated with High Capacity cDNA Reverse Transcription kit (Applied Biosystems) and RT-qPCR was performed with Power SYBR Green PCR Master Mix (Life Technologies, Cat. No. 1408470, Austin, TX) and gene‐specific primers as published before^12^. 7900HT Quantitative Real time qPCR system (Applied Biosystems) with 384-well block was used for signal detection. Expression was normalized with ΔΔCT method. The geometric mean of GAPDH and B-actin was used for normalization, Hu8132 PHH sample was used as reference for *SOX17*, *FOXA2*, *AFP* and *ALB* while iPSC sample 11,304.EURCCs was used as reference for *OCT4*.

### Studying the microRNA (miRNA) expression

#### MicroRNA microarray

Altogether 17 randomly selected cell lines from 12 patients (four from each of the three patient groups) were used for microarray screening. Pooled RNA samples were prepared from three biological replicates from each cell line and differentiation time point (iPSC, DE, HLC) with each replicate contributing equal amount of total RNA. In addition, HepG2 cells and Hu8210 PHHs day 0 were used as reference samples. Adequate RNA quality (RNA integrity number > 6) was confirmed with Agilent 2100 Bioanalyzer.

The miRNA expression profile was determined with Agilent SurePrint G3 human miRNA (8 × 60 K) microarrays containing probes for all Sanger miRBase release 21 human miRNAs from 17 Homo sapiens cell culture samples. High-Resolution Microarray Scanner G2505C and Agilent Feature Extraction software (11.0.1.1) were used to process microarray signals. Microarray raw data was corrected for background, normalized and log-2-scaled with robust multi-array average (RMA) algorithm. iPSC samples UTA.11916.EURCSp and UTA.12212.EURCAs were identified as outliers and excluded from further analyses.

#### Validation of miRNA expression by RT-qPCR

We selected miRNAs for validation based on low *p* value and expression trend in the microarray analyses as well as the relevance of the miRNAs to CAD and metabolic dysfunction based on published literature. The RNA samples were collected as presented in Section "RNA sample collection, RNA extraction and real-time quantitative PCR " and cDNA was generated from 10 ng of total RNA with Advanced miRNA cDNA synthesis kit (Applied Biosystems). MicroRNA expression was measured with TaqMan Advanced miRNA assays (Table S3) and TaqMan Fast Advanced Master Mix with BioRad CX384 Real-Time PCR Detection system according to miRNA assay instruction. Expression was normalized with ΔΔCT method. Mean of hsa-miR-16-5p and hsa-miR-423-3p expression was used for normalization and corresponding median of control individuals’ samples was used as reference for each assay.

### Statistical and bioinformatic analysis

Statistical and bioinformatic analysis were carried out with R (4.0.2). Differential miRNA expression analysis for miRNA microarray data was carried out by fitting a linear regression model with limma R-package^17^. When analysing HLCs, the linear model was adjusted for expression of hsa-miR-302a-5p, a miRNA specific to undifferentiated cells^18^. Only miRNAs that were expressed in all compared biological groups were included in differential miRNA expression analysis (differentiation stages n = 380, HLCs n = 433). A miRNA was considered expressed if its signal was above threshold in at least half of the samples.

Hierarchical clustering analysis of samples was performed to scaled expression data matrix using Pearson’s correlation distance and average linkage method. Clustering of the rows was performed with k-means clustering.

Six lists of miRNAs were generated for target prediction and pathway analysis based on pairwise comparison of patient groups’ miRNA microarray expression. Each list included either down- or up-regulated (*p* < 0.05) miRNAs of a compared pair as well as miRNAs considered expressed in one but not the other group (Table S4). Targets of each miRNA list were predicted and further analysed for pathway (Reactome, KEGG) and function (Gene Onthology) enrichment with miRWalk miRNA target prediction tool^19^. Pathway analysis is based on the hyper geometric test (chi-square selection algorithm) and was carried out from targets predicted by both TarPmiR and Targetscan and by TarPmir and MiRTarBase. Gene ratio was calculated as hits divided by population hits from the miRWalk output. Pathways with adjusted *p*-value (FDR) < 0.05 and gene ratio > 0.2 were considered significant.

Mean was used as a central tendency measure and error bars represent standard error of mean in all but RT-qPCR miRNA analysis where median and boxplots were used. Kruskall-Wallis was used to test the significance of group differences in the latter case followed by pairwise assessment with Mann–Whitney test.

## Results

### The iPSC characterisation and hepatic differentiation of patient-specific iPSCs to HLCs

The iPSC lines used in this study that had not previously been used in any publication, were characterized for pluripotency and the results are presented in Figs. S1,S2.

Two iPSC lines from each patient were differentiated to HLCs, except for three patients in which case only one cell line was successfully differentiated (Table S1). At the DE stage (Fig. S3), pluripotency marker OCT4 was down-regulated and cells expressed Sox17 and FoxA2 (Fig. S4A–C), which are involved in development of multiple endoderm-derived organ systems^20^. 60–96% of cells at DE stage were CXCR4 positive (Fig. S5A). Functional HLCs were obtained from all patients (Fig S6). Cells expressed *albumin* (*ALB*) and *alfa-fetoprotein* (*AFP*) at transcriptional level (Fig. S4D,E) and ALB, AFP, asialoglycoprotein receptor 1 (ASGPR1) and low-density lipoprotein (LDL) receptor at protein level (Figs. S7–S10). HLCs demonstrated ability to secrete urea, triglycerides and albumin and ability to uptake labelled LDL (Fig. S5B–D, Figs. S7–S10).

### The iPSCs, DE and HLCs have distinct miRNA profiles

**Figure 1:**
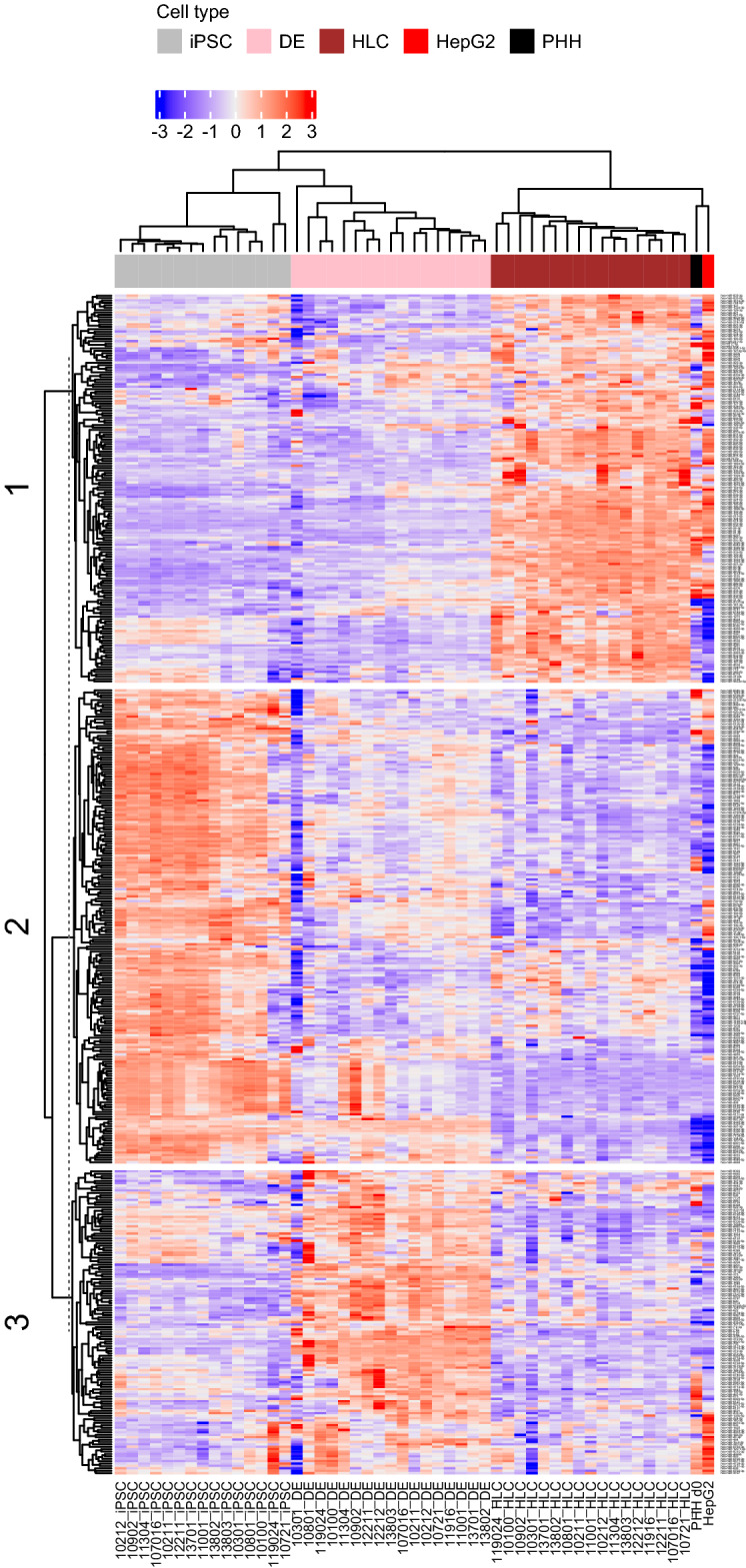
The miRNA expression profile changes during hepatocyte differentiation from iPSCs through DE to HLCs. Heatmap includes differentially expressed (FDR < 0.05) miRNAs between differentiation stages as well as miRNAs expressed only in one or two differentiation stages. Clusters 1, 2 and 3 represent miRNAs that were specific to HLCs, iPSCs and DEs, respectively. The iPSC samples from two cell lines (UTA.11916.EURCSp and UTA.12212.EURCAs) were identified as outliers and excluded from the analysis. iPSC, induced pluripotent stem cells; DE, definitive endoderm; HLC, hepatocyte-like cells; PHH, primary human hepatocyte; HepG2, liver hepatocellular carcinoma cell line.

**Figure 2:**
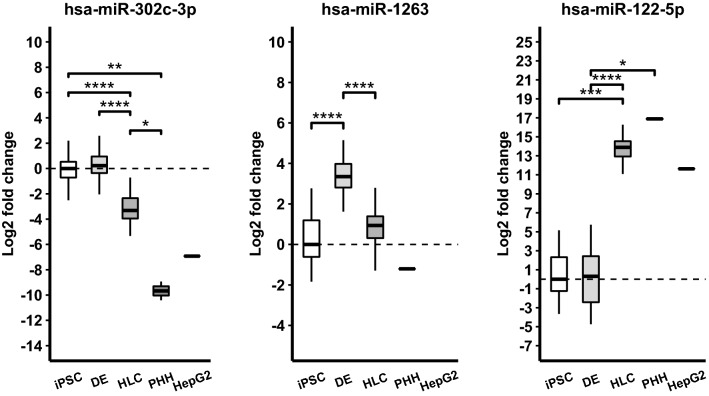
Validation of differentiation stage-specific miRNAs with RT-qPCR. miR-302c-3p, miR-1263 and miR-122-5p have high expression in iPSCs, DEs and HLCs, respectively. Expression of miR-1263 was undetectable in HepG2 cells. Brackets represent *p* value of pairwise Mann–Whitney test: *p < 0.05, **p < 0.01, ***p < 0.001, ****p < 0.0001. HepG2 cells were left out of pairwise comparison.

Microarray data analysis of 17 cell lines determined 241 differentially expressed miRNAs between iPSCs and DEs (FDR adjusted *p* value < 0.05). In addition, 220 miRNAs were differentially expressed between DEs and HLCs (Table S5). Altogether 69, 18 and 56 miRNAs were expressed only in iPSCs, DE and HLC, respectively, when the three cell types were compared (Table S6). Hierarchical clustering showed closer resemblance of DEs to iPSCs rather than HLCs, whereas miRNA profile of HLCs was closer to that of PHHs and HepG2 cells (Fig. 1). Expression of three differentiation stage-specific miRNAs, miR-302c-3p^21^, miR-1263^22^ and miR-122-5p^23^ was further validated by RT-qPCR in all 27 cell lines (Fig. 2). The mir-1263 was not detected in the HepG2 cells.

### Several miRNAs were differentially expressed in HLCs derived from the CAD cell lines compared to the control cell lines

**Figure 3:**
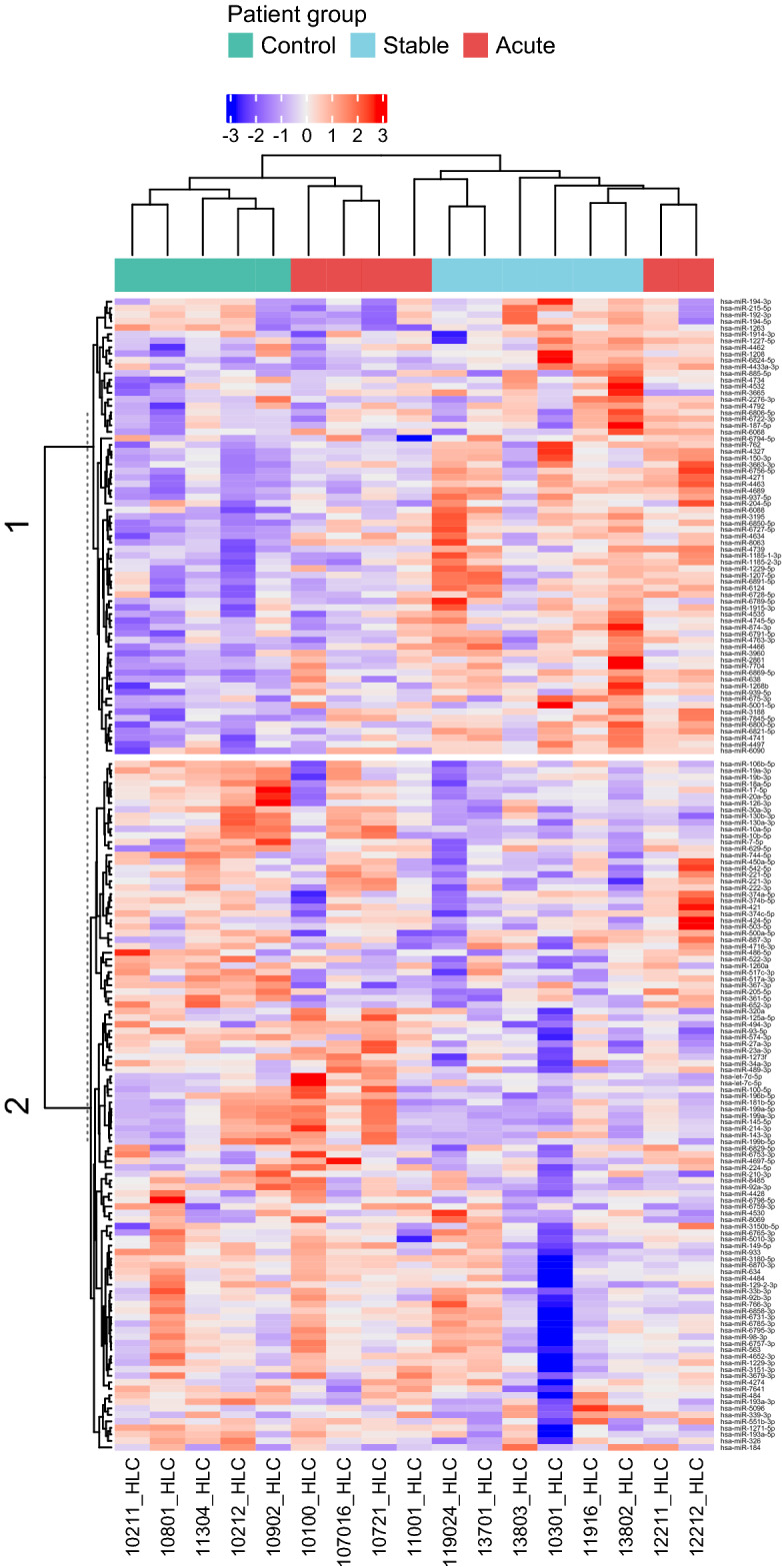
Heatmap of differentially expressed miRNAs (*p* < 0.05) in HLCs based on miRNA microarray. Heatmap also includes miRNAs expressed only in one or two patient groups. Cluster 1 contains miRNAs that were up-regulated in most of the CAD cell lines and cluster 2 contains miRNAs that were mostly down-regulated in the CAD cell lines compared to the control group. HLC, hepatocyte-like cell.

Based on the microarray data altogether 80 miRNAs were differentially expressed between CAD, including both stable and acute, and control cell lines (Table S7). Moreover, altogether 35 miRNAs were differentially expressed (*p* < 0.05) between control and acute CAD cell lines, 87 miRNAs between control and stable CAD cell lines and 18 between stable and acute CAD cell lines in pairwise comparison (Table S8). In addition, several miRNAs were found to be expressed in either one or two patient groups (Table S9). In addition, miRNA profiles of control and stable CAD cell lines appeared to be uniform within their own groups, while acute CAD cell lines showed more variation (Fig. 3).

**Figure 4:**
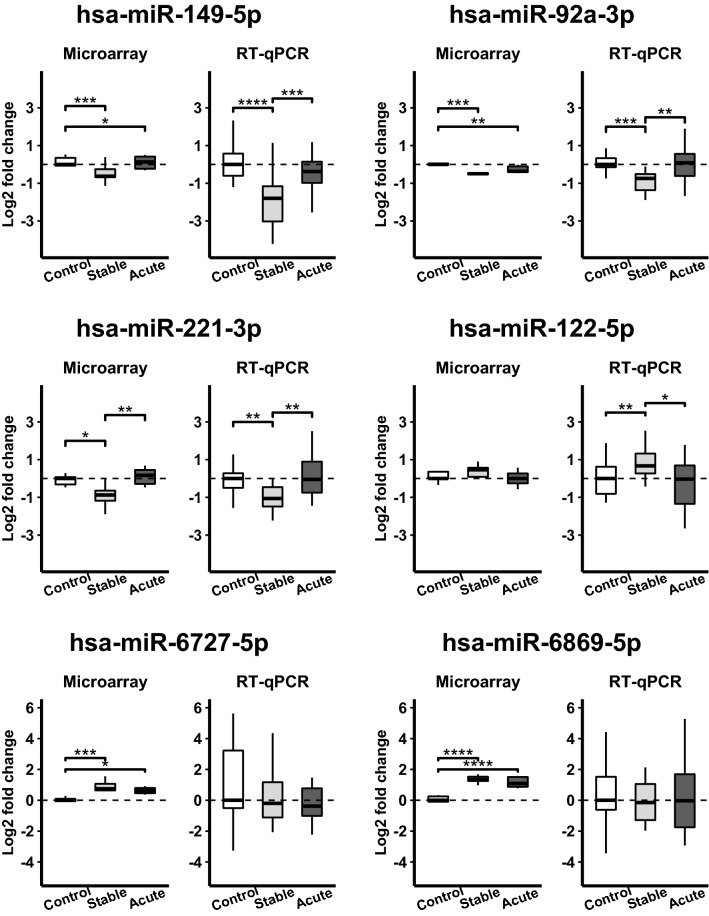
Validation of differentially expressed miRNAs in the studied patient groups by RT-qPCR. Brackets represent *p* value of pairwise Mann–Whitney test in RT-qPCR figures and *p* value of pairwise limma comparison in microarray data. *p < 0.05, **p < 0.01, ***p < 0.001, **** *p* < 0.0001.

Based on the low *p* value, expression trend in the microarray analysis and relevance to CAD or metabolic dysfunction according to the literature, six miRNAs were selected for further RT-qPCR validation in all 27 cell lines. The miRNAs miR-149-5p, miR-92a-3p, miR-221-3p and miR-122-5p replicated the expression pattern of microarray screening results (Fig. 4). Surprisingly, miR-6869-5p and miR-6727-5p were non-significant in RT-qPCR analysis despite their differential expression in the microarray screening.

### Predicted targets of differentially expressed miRNAs suggest a role in sphingolipid metabolism and insulin signalling

**Figure 5:**
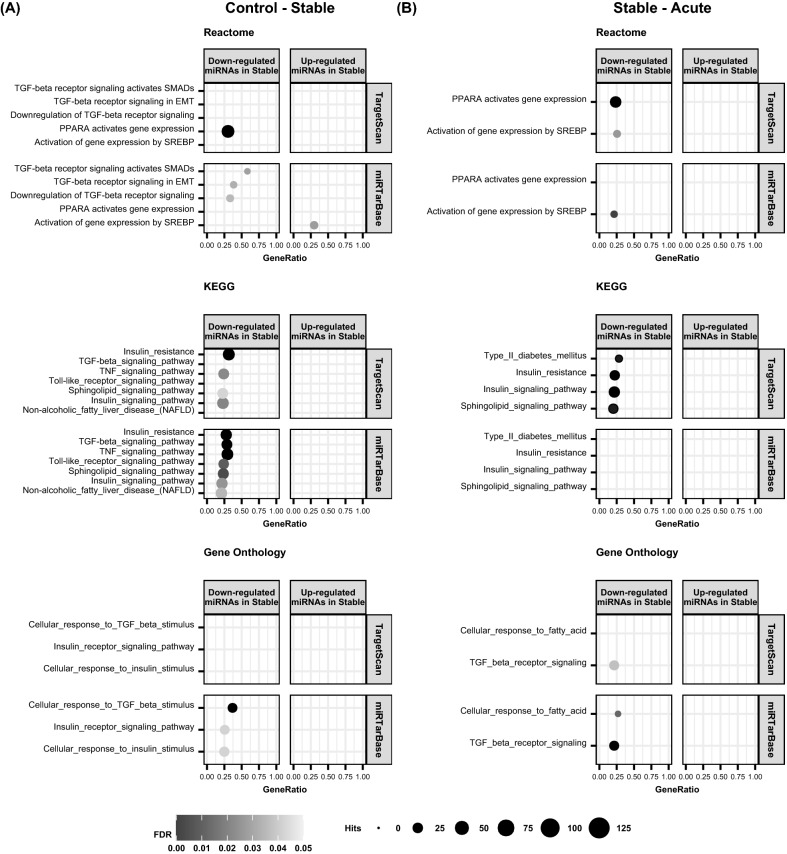
Selected pathways from pathway enrichment analysis of predicted miRNA targets. (**A**) Analysis of miRNAs either down- or up-regulated in the stable CAD patients compared to the controls. (**B**) Analysis of miRNAs either down- or up-regulated in the stable CAD patients compared to the acute CAD patients. Analysis was carried out either with the targets predicted by TarPmiR and computational algorithm by TargetScan or TarPmiR and experimentally validated target database miRTarBase. Only pathways that were statistically significant (FDR < 0.5) and pathways with gene ratio > 0.2 are presented. Bubble size represents the amount of hits within the pathway from the predicted target genes. Gene ratio represents the ratio of the predicted targets within a pathway and all genes in that pathway.

Differentially up- or down-regulated miRNAs and those expressed in only one of the groups were analysed with miRWalk target prediction and pathway enrichment tools to shed light on possible function of those miRNAs. First targets predicted with both TarPmiR and Targetscan computational algorithms were analysed (Figs. S11–S13, Table S10^24^). By combining observations from the Reactome, KEGG and GO pathway enrichment analyses we observed that miRNAs down-regulated in the stable CAD patients compared to the acute CAD patients and controls are involved in regulation of e.g. peroxisome proliferator-activated receptor alpha (PPARα) activated gene expression, insulin resistance, insulin signalling and sphingolipid signalling pathways (Fig. 5A,B). In addition, miRNAs down-regulated in the stable CAD compared to the acute CAD patients are possibly involved in regulation of activation of gene expression by sterol regulatory element-binding protein (SREBP) and type II diabetes mellitus pathways (Fig. 5B).

We then carried out pathway enrichment analysis with targets from both TarPmiR and miRTarBase, which included only experimentally validated miRNA targets (Figs. S14–S16, Table S11^24^). The analysis of Reactome, KEGG and GO showed that miRNAs down-regulated in the stable CAD patients compared to the control individuals could regulate insulin resistance, insulin signalling, sphingolipid signalling and non-alcoholic fatty liver disease (NAFLD) pathways. Also, inflammatory pathways related to tumour necrosis factor (TNF), transforming growth factor β (TGF-β) and toll-like receptor signalling could be regulated by these miRNAs (Fig. 5A). The miRNAs up-regulated in the stable CAD patients compared to controls could regulate activation of gene expression by SREBP (Fig. 5A). The miRNAs that were down-regulated in the stable CAD patients compared to the acute CAD patients were possibly involved in pathways such as activation of gene expression by SREBP, cellular response to fatty acid and TGF beta receptor signalling (Fig. 5B). MicroRNAs up-regulated in the acute CAD patients compared to controls could regulate activation of gene expression by SREBP (Fig. S14).

## Discussion

We studied miRNA profile of cells during the differentiation process from iPSCs through DE cells into iPSC-HLCs, and furthermore in different patient groups: stable CAD, acute CAD and healthy individuals. The miRNA profile of the cells changed during the hepatic differentiation process and reproduced the miRNA profile of differentiation stages described before^25^. In our study, the miRNA profiles of iPSC-HLCs closely resembled that of the PHHs and HepG2 cells. The miRNA microarray analyses of the iPSC-HLCs from stable and acute CAD patients compared to healthy individuals showed that especially the miRNA profiles of stable CAD patients formed a distinct group, while profiles of acute CAD patients were more heterogeneous. In our miRNA microarray analyses, we found a total of 80 miRNAs differentially expressed between all CAD patients versus the healthy controls. More specifically, 35 and 87 miRNAs were statistically significantly deregulated in the acute and stable CAD patients, respectively, compared to controls in a pairwise comparison. We successfully validated by RT-qPCR the differential expression pattern of miR-149-5p, miR-92a-3p, miR-221-3p and miR-122-5p between the disease groups, the first three down-regulated and miR-122-5p up-regulated in the stable CAD compared to other groups.

Our miRNA target prediction analyses showed that miRNAs down-regulated in the stable CAD patients compared to both the controls and the acute CAD patients could be involved in lipid metabolism by targeting genes regulated by PPARα and SREBPs. PPARα is known to regulate hundreds of genes involved in e.g. hepatic fatty acid uptake and fatty acid oxidation. PPARα agonists such as fibrates raise plasma HDL and reduce plasma triglycerides^26^. SREBPs regulate genes involved in cholesterol biosynthesis^27^, and they are also involved in cholesterol-inducible inflammation along with other transcriptional regulators like NF-κB, AP-1, C/EBPs^28^. Typically, miRNAs down-regulate protein expression and since these miRNAs were down-regulated in the stable patients in our study, we hypothesized that these pathways could be less suppressed in the stable patients compared to the controls and acute CAD patients. Pathway analysis suggested that down-regulation of these miRNAs could also result in higher insulin resistance, which is a significant risk factor for metabolic disease. Furthermore, we saw predicted targets of down-regulated miRNAs in the stable CAD to be involved in sphingolipid signalling. Sphingolipids, such as ceramides are bioactive lipids, which are largely produced by the liver, play a role in regulation of inflammation, apoptosis, steatosis as well as insulin resistance, and are implicated in contributing to NAFLD and CAD^29^. Thus, in our study, the signalling would be increased in the cells of stable CAD patients, possibly contributing to atherogenesis.

We then validated expression of four miRNAs by RT-qPCR. We found miR-149 down-regulated in the stable CAD compared to other groups, concurring with a previous study in which circulating miR-149 was found to be down-regulated in stable CAD and unstable angina patients in a study of 69 angiographically documented geriatric patients^30^. Reported functions of miR-149-5p in the liver indicate it to promote lipogenesis in HepG2 cells by down-regulating FGF-21^31^. MiR-149 was down-regulated in the livers of NAFLD mice, and administration of miRNA-149-mimic to these mice resulted in elevation of lipid metabolism proteins SCD-1, PPARα and ABCA1 and reduced fatty tissue. Inflammation was reduced through ATF6 down-regulation with high levels of miR-149^32^. The miR-149 has also been found up-regulated in the liver of high-fat-diet mice and is possibly associated with insulin resistance^31,33^. Thus, our findings of significantly lower levels of miR-149-5p in stable CAD patients support its active role in the development of atherosclerosis through regulating genes involved in the lipid metabolism as well as suppressing STAT3- or ATF6-mediated inflammation^31,34^.

We found miR-92a-3p to be decreased in iPSC-HLC from stable CAD group, but not those from those suffering from acute CAD. This is in line with previous studies showing circulating miR-92a to be down-regulated in patients with (stable) CAD^35,36^ and increased in patients with acute coronary syndrome, especially in patients with T2DM^37^. MicroRNA-92a is well known for its association with atherosclerosis, especially via its functions in endothelial cells^38^. Moreover, in hyperlipidemic hamsters, inhibition of miR-92 led to reduction of total cholesterol in plasma and reduction of cholesterol, triglycerides and free fatty acids in the liver^39^. So, apart from endothelial cells, miR-92a is expressed in the liver, where it might contribute to atherogenesis via regulation of lipid metabolism. Interestingly, a previous study showed that miR-92a levels are different in different HDL subpopulations secreted by the liver (miR-92 prevailing in the HDL_3_), and the levels are lower in stable CAD compared to vulnerable patients^40^. Similarly, lower level of mir-92a in the HLCs derived from stable CAD patients in our study suggests a protective role against acute events but to explicitly clarify whether this is through regulation of lipid metabolism or other mechanisms related to e.g. endothelial cell functionality, requires further studies.

The miR-221-3p was decreased in the stable CAD patients compared to other groups both in the miRNA microarray and in the qPCR validation experiments in our study. This concurs with an earlier study, where circulating miR-221 was found down-regulated in stable CAD^41^. The miR-221 is involved in atherogenic processes in the endothelium and atherosclerotic lesions^42^, and it has been shown to have opposite effects in vascular smooth muscle cells and vascular endothelial cells regarding proliferation, migration and apoptosis^43^. Decreased circulatory level of miR-221-3p has been associated with isolated low HDL level, which is an independent CVD risk factor. In the liver, miR-221 has been found to inhibit apoptosis, accelerate hepatocyte proliferation and to be involved in hepatocarcinogenesis^44,45^. Knockout and knockdown of miR-221 in non-alcoholic steatohepatitis (NASH) mouse model reduced hepatic triglyceride content, increased slightly serum triglyceride levels and reduced inflammation and fibrosis^46^. The mir-221 is heavily involved in vascular remodelling along with miR-222, and can contribute to cardiovascular pathology through its effects on fat and glucose metabolism in nonvascular tissues such as adipose tissue, liver, and skeletal muscles^42^. Our results support the miR-221 involvement in vascular remodelling as it was clearly down-regulated in the iPSC-HLCs derived from stable CAD patients but its effect assumably vary depending on the target cell type as well as the arterial territory^47^.

MicroRNA-122 is a highly liver-specific miRNA, which has an important role in controlling metabolic homeostasis, specifically by regulating hepatic cholesterol and lipid metabolism and by promoting hyperlipidemia^48^. In our study, the level of miR-122-5p was higher in the iPSC-HLC from stable CAD patients compared to controls, which concurs with a previous study showing higher levels of both circulating miR-122 and miR–126 in patients with angiographically significant CAD compared to controls^49^. Expression of hepatic miR-122 has also been shown to correlate with circulating miR-122 in patients with NAFLD, which would support that higher expression of miR-122 in stable CAD HLCs’ could correlate with higher secreted miR-122^50^. Interestingly, increased plasma levels of miR-122 have earlier been associated with hyperlipidemia, and were positively correlated with total cholesterol, triglyceride, and LDL-cholesterol levels as well as severity of CAD^51^. A direct association of circulatory miR-122-5p with subfractions of small VLDL, IDL and large LDL, as well as apoB levels has previously been shown, indicating a connection of miR-122-5p with cholesterol levels^52^. This is also supported by animal studies, which have suggested that miR-122 expression controls cholesterol synthesis and VLDL secretion^48,53^. High levels of miR-122 in HLCs from the stable CAD patients in our study, could point to increased cholesterol synthesis and VLDL secretion in these cells.

We expect the iPSC-HLCs in our model to mainly reflect genetic differences in miRNA expression as we assume that the lifestyle effect mostly disappears when skin fibroblasts are reprogrammed into iPSCs. In addition, skin fibroblasts are most likely not altered by the lifestyle effects in a similar manner as the liver in these patients. The patients were carefully selected for this study to try to recapitulate two distinct and exaggerated CAD phenotypes. Stable CAD patients were intentionally older, with extensive atherosclerotic lesions in coronary arteries but no history of MI, whereas acute CAD patients were younger and had a single lesion that led to MI. Most of the CAD patients had strong family history of cardiovascular events. The stable CAD patients had extensive stenosis in coronary arteries. Thus, it is perhaps not surprising that the patients’ iPSC-HLCs share a distinct miRNA profile, which seems to promote metabolic dysfunction as well as triglyceride and cholesterol synthesis and secretion, which is then deposited in the coronary arteries. On the other hand, our acute CAD patients presented only one atherosclerotic lesion which led to MI, suggesting that possibly substantial lipid accumulation is not in the central role in these patients. Alternatively, atherogenesis and plaque rupture in the acute CAD patients is not conveyed by congenital miRNA expression pattern but instead by other genes or other still unknown factors. Interestingly, while miRNA profiles of acute CAD cell lines showed more variation within the group than those of controls and stable CAD (Fig. 3), the cell lines of the same acute CAD patients clustered together. This suggests that our model captures true genetic differences as opposed to reflecting e.g. conditions of differentiation process.

One limitation of the study could be uncertainty related to variation in the maturity and differentiation efficiency of iPSC-HLCs. We tackled this problem by using two iPSC-HLC cell lines per patient as well as adjusting for the amount of iPSC-specific miR-302a in our linear model when comparing iPSC-HLCs of the disease groups. We also demonstrated that miRNA profile changed during differentiation and the miRNA profile of iPSC-HLCs closely resembled that of the PHHs. Another limitation is the limited number of patients that participated in this study, which could result in lack of statistical power or patient heterogeneity affecting the results. To avoid the latter, special attention was given to patient selection based on their distinct CAD phenotypes. Still, while convincingly reflecting the miRNA expression and possible connection of miRNAs to atherosclerosis in these particular individuals, these results might not be fully generalized to a larger population. Moreover, higher number of miRNAs in validation studies might have identified even more relevant miRNAs. Nevertheless, our iPSC-HLC model is a useful tool to study e.g. hepatic lipid and lipoprotein metabolism underlying the development of atherosclerosis in a patient-specific manner without invasive liver biopsy.

In summary, we found that the iPSC-HLCs derived from the stable CAD patients had a distinct genetic miRNA profile that could influence lipid metabolism, sphingolipid and insulin signalling as well as inflammation processes. This in turn, could provide a liver-driven explanation to the susceptibility of these patients to developing CAD. It is possible that the acute patients differ from stable patients due to the limited amount of atherosclerosis observed in their coronaries and liver-specific miRNAs do not explain susceptibility to acute plaque ruptures. Our iPSC-HLC model can be used as a patient-specific in vitro model to study how e.g. increased insulin or glucose levels or inflammatory factors affect the miRNA expression in the hepatocytes derived from patients with different CAD phenotypes. Moreover, the model can be utilised to study specific miRNA target genes or miRNA and their target effects on lipid/lipoprotein production in vitro.

## Supplementary Information

Supplementary Information 1.

Supplementary Table S10.

Supplementary Table S11.

## Data Availability

Additional data is available in the supplementary data and at Mendeley data: https://doi.org/10.17632/5wsbncgvj5.1.

## Abbreviations

iPSC: Induced pluripotent stem cell
DE: Definitive endoderm
HLC: Hepatocyte-like cell
CAD: Coronary artery disease
miRNA: MicroRNA
DE: Definitive endoderm
PHH: Primary human hepatocyte
