# Supplementary material for: Coronary artery disease patient-derived iPSC-hepatocytes have distinct miRNA profile that may alter lipid metabolism
Source: Sci Rep. 2023 Jan 30;13:1706. doi: 10.1038/s41598-023-28981-7 (PMC9886909; doi:10.1038/s41598-023-28981-7)
Supplement: Supplementary file 1 — Supplementary Information 1. [file 41598_2023_28981_MOESM1_ESM.pdf]

Coronary artery disease patient-derived iPSC-hepatocytes have distinct miRNA profile that may alter lipid metabolism

Anna Alexanova[1], Emma Raitoharju[1,2,3], Joona Valtonen[1], Katriina Aalto-Setälä[1] & Leena E. Viiri[1,*]

[1]The Cardiovascular Research Center Tampere, Faculty of Medicine and Health Technology, Tampere University, Finland.

[2]Molecular Epidemiology, Faculty of Medicine and Health Technology, Tampere University, Tampere, Finland.

[3] Tampere University Hospital, Tampere, Finland.

[*]Corresponding author, email: leena.viiri@tuni.fi; Arvo Ylpön katu 34, 33520 Tampere, Finland.

Supplementary Figures and legends

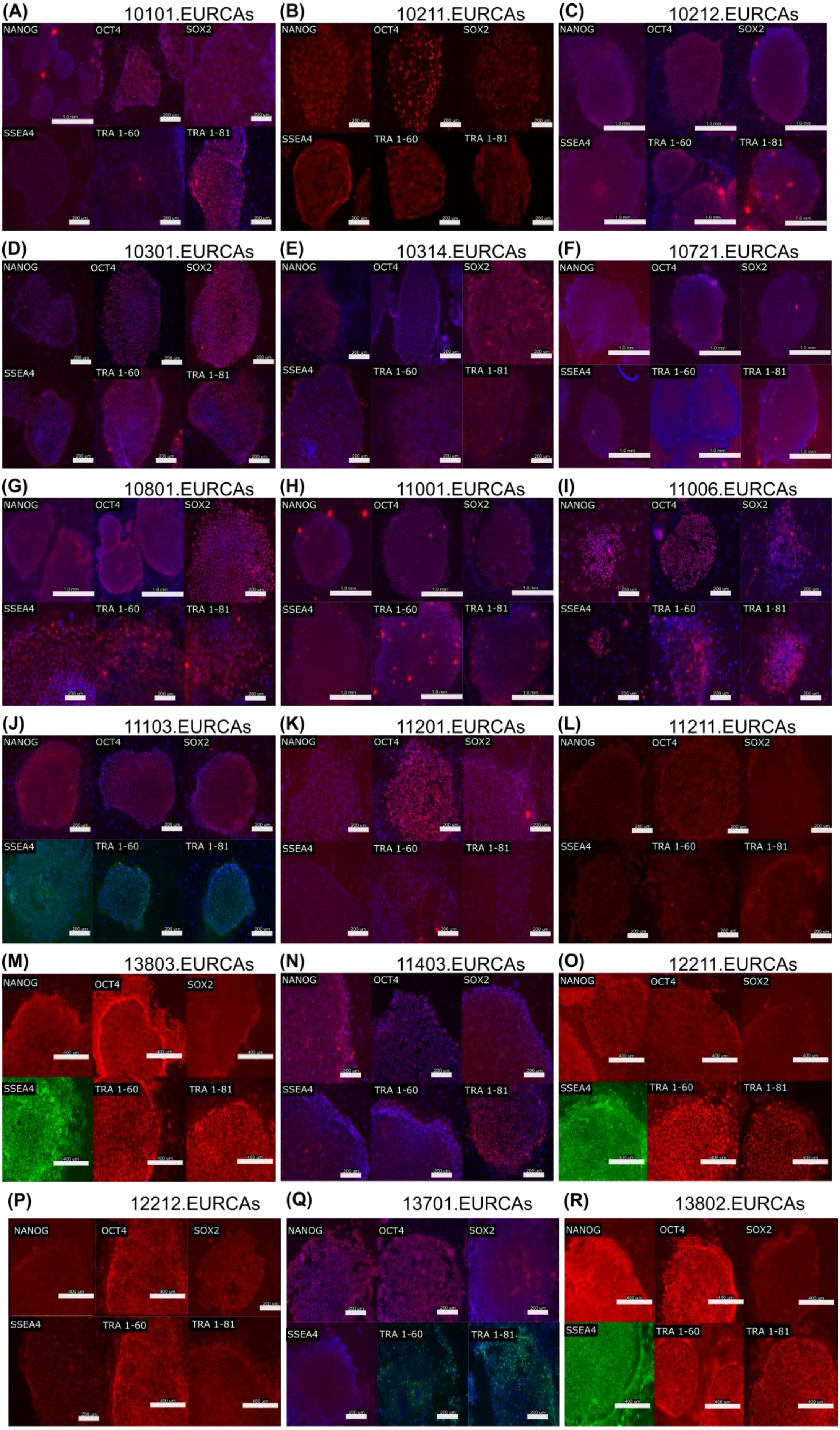

Figure S1. Expression of pluripotency markers by induced pluripotent stem cells (iPSCs) detected by immunocytochemistry.

Figure S2.

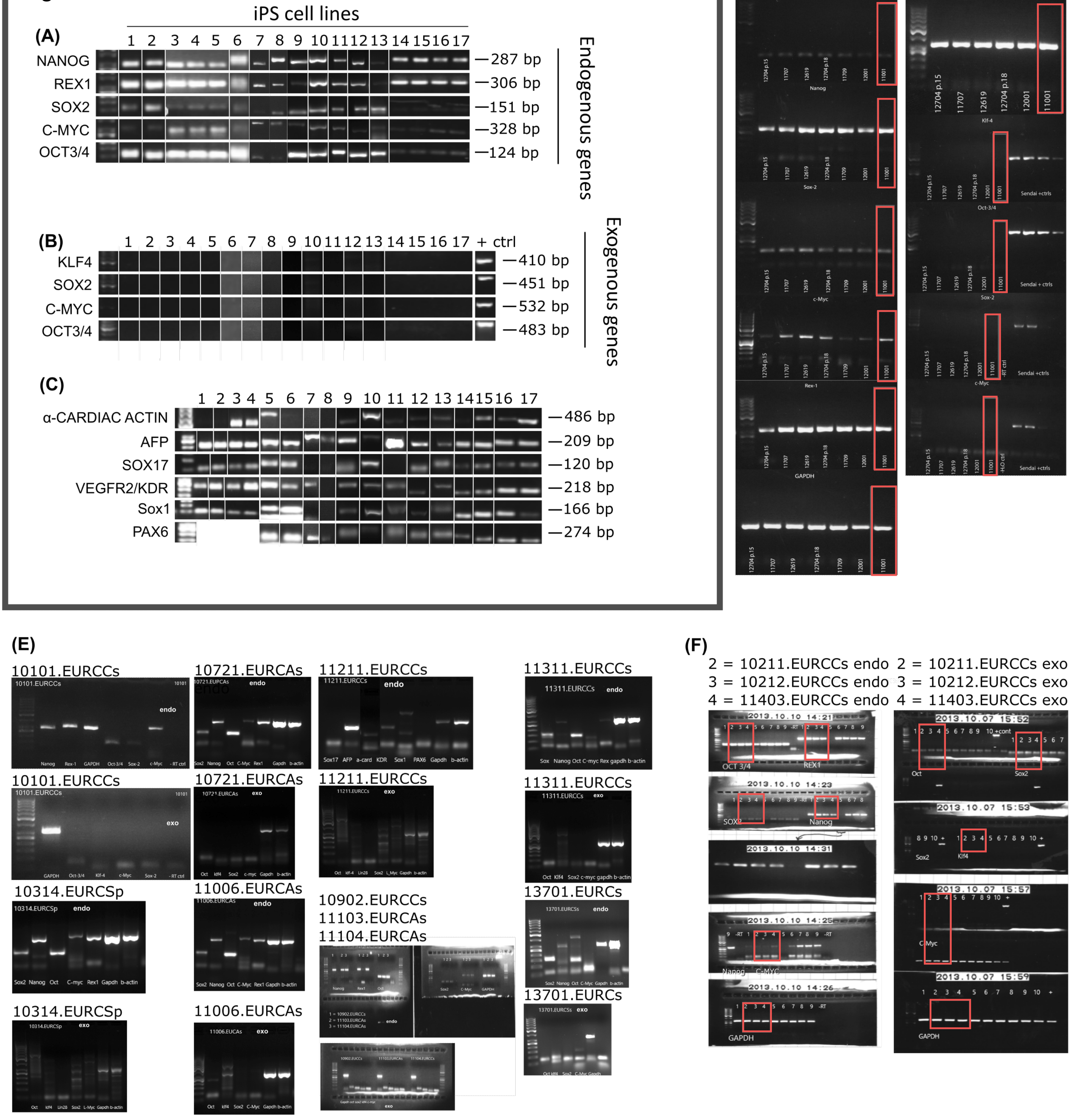

iPS cell lines
(A)
1 2 3 4 5 6 7 8 9 10 11 12 13 14 15 16 17
NANOG
REX1
SOX2
C-MYC
OCT3/4
—287 bp
—306 bp
—151 bp
—328 bp
—124 bp
Endogenous genes
(B)
1 2 3 4 5 6 7 8 9 10 11 12 13 14 15 16 17 + ctrl
KLF4
SOX2
C-MYC
OCT3/4
—410 bp
—451 bp
—532 bp
—483 bp
Exogenous genes
(C)
1 2 3 4 5 6 7 8 9 10 11 12 13 14 15 16 17
α-CARDIAC ACTIN
AFP
SOX17
VEGFR2/KDR
Sox1
PAX6
—486 bp
—209 bp
—120 bp
—218 bp
—166 bp
—274 bp
(D) 11001.EURCAs
Oct-3/4
Nanog
Sox-2
c-Myc
Rex-1
GAPDH
Klf-4
12704 p.15
11707
12619
12704 p.18
11709
12001
11001
Sendai +ctrls
(E)
10101.EURCCs
10721.EURCAs
11211.EURCCs
11311.EURCCs
10314.EURCSp
11006.EURCAs
10902.EURCCs
11103.EURCAs
11104.EURCAs
13701.EURCs
endo
exo
(F)
2 = 10211.EURCCs endo
3 = 10212.EURCCs endo
4 = 11403.EURCCs endo
2 = 10211.EURCCs exo
3 = 10212.EURCCs exo
4 = 11403.EURCCs exo
OCT 3/4
REX1
SOX2
Nanog
C-MYC
GAPDH
Oct
Sox2
Klf4
C-Myc

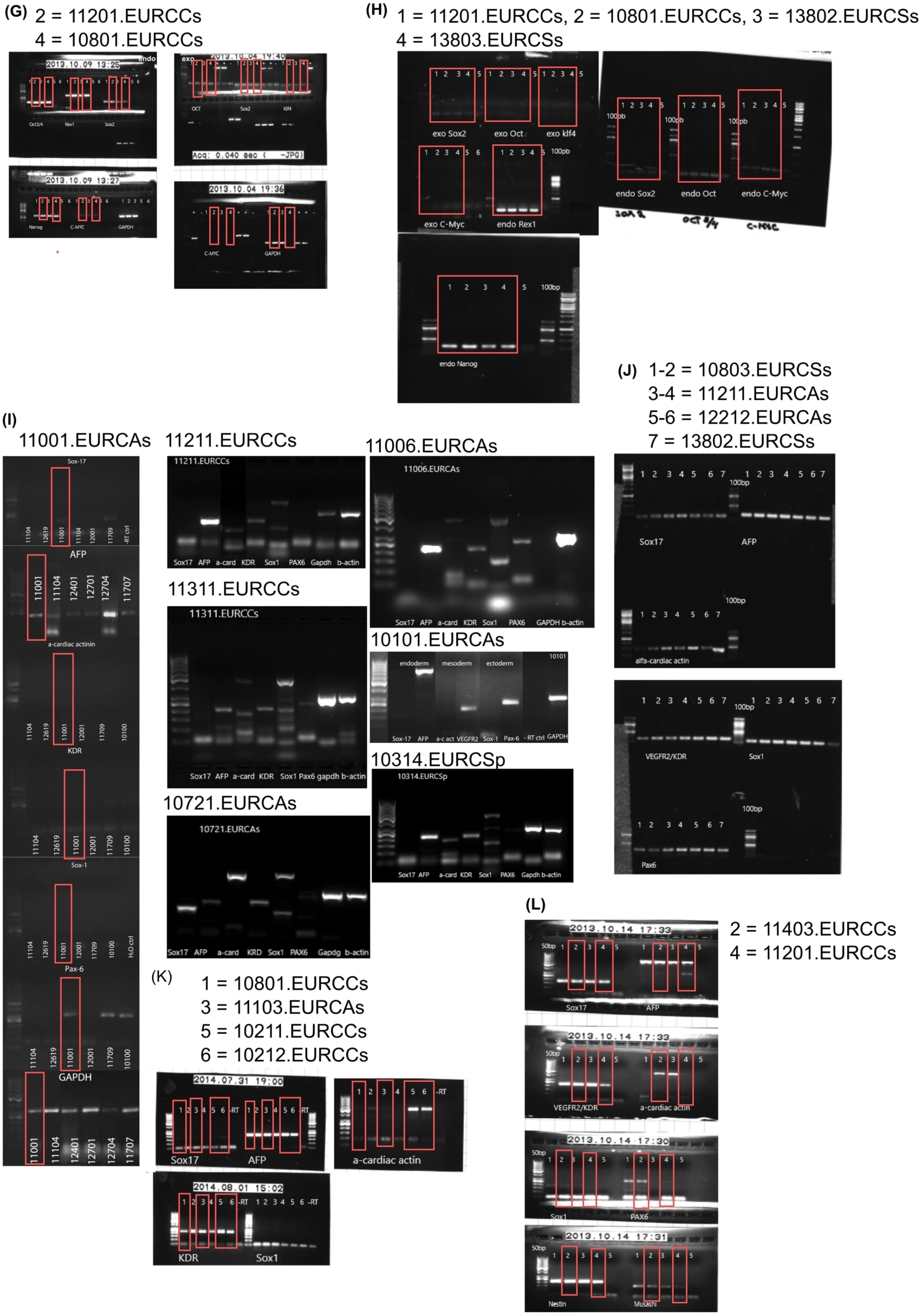
(G)
2 = 11201.EURCCs
4 = 10801.EURCCs
(H)
1 = 11201.EURCCs, 2 = 10801.EURCCs, 3 = 13802.EURCSs
4 = 13803.EURCSs
exo Sox2
exo Oct
exo klf4
exo C-Myc
endo Rex1
endo Sox2
endo Oct
endo C-Myc
endo Nanog
(J)
1-2 = 10803.EURCSs
3-4 = 11211.EURCAs
5-6 = 12212.EURCAs
7 = 13802.EURCSs
(I)
11001.EURCAs
11211.EURCCs
11006.EURCAs
11311.EURCCs
10101.EURCAs
10314.EURCSp
10721.EURCAs
(K)
1 = 10801.EURCCs
3 = 11103.EURCAs
5 = 10211.EURCCs
6 = 10212.EURCCs
(L)
2 = 11403.EURCCs
4 = 11201.EURCCs

Figure S2. Characterization of induced pluripotent stem cells (iPSCs) by PCR. **(A)** Expression of pluripotency marker genes. **(B)** Absence of the expression of exogenes which were used during the reprogramming process. **(C)** Expression of genes from the three embryonic germ layers, endoderm, mesoderm and exoderm after the differentiation of iPSCs into embryoid bodies.

Full-length gels showing expression of pluripotency marker genes and absence of exogenes **(D)** 11001.EURCAs. **(E)** 10101.EURCAs, 10314.EURCSp, 10721.EURCAs, 11006.EURCAs, 11211.EURCCs, 11311.EURCCs, 13701.EURCSs. **(F)** 10211.EURCCs (F2), 10212.EURCCs (F3), 11403.EURCCs (F4). **(G)** 11201.EURCCs (G2), 10801.EURCCs (G4). **(H)** 12211.EURCAs (H1), 12212.EURCAs (H2), 13802.EURCSs (H3), 13803.EURCSs (H4)

Full-length gels showing expression of genes from the three embryonic germ layers, endoderm, mesoderm and exoderm after the differentiation of iPSCs into embryoid bodies. **(I)** 11001.EURCAs, 11211.EURCCs, 11311.EURCCs, 10721.EURCAs, 11006.EURCAs, 10101.EURCAs, 10314.EURCSp. **(J)** 13803.EURCSs (J1,2), 12211.EURCAs (J3,4), 12212.EURCAs (J5,6), 13802.EURCSs (J7). **(K)** 10801.EURCCs (K1), 11103.EURCAs (K3), 10211.EURCCs (K5), 10212.EURCCs (K6). **(L)** 11403.EURCCs (L2), 11201.EURCCs (L4).

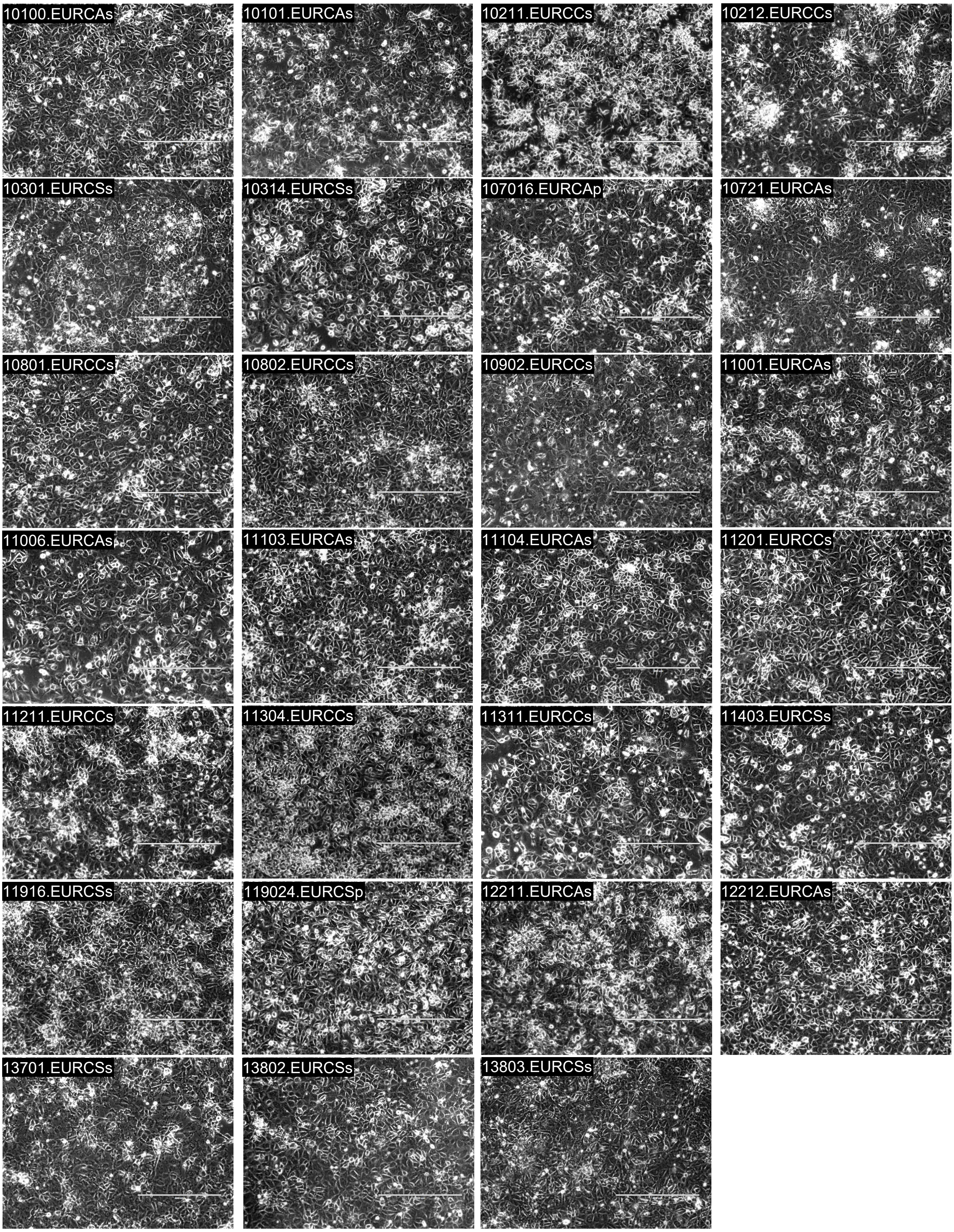

Figure S3. Definitive endoderm cells. Scale bar 400 µm.

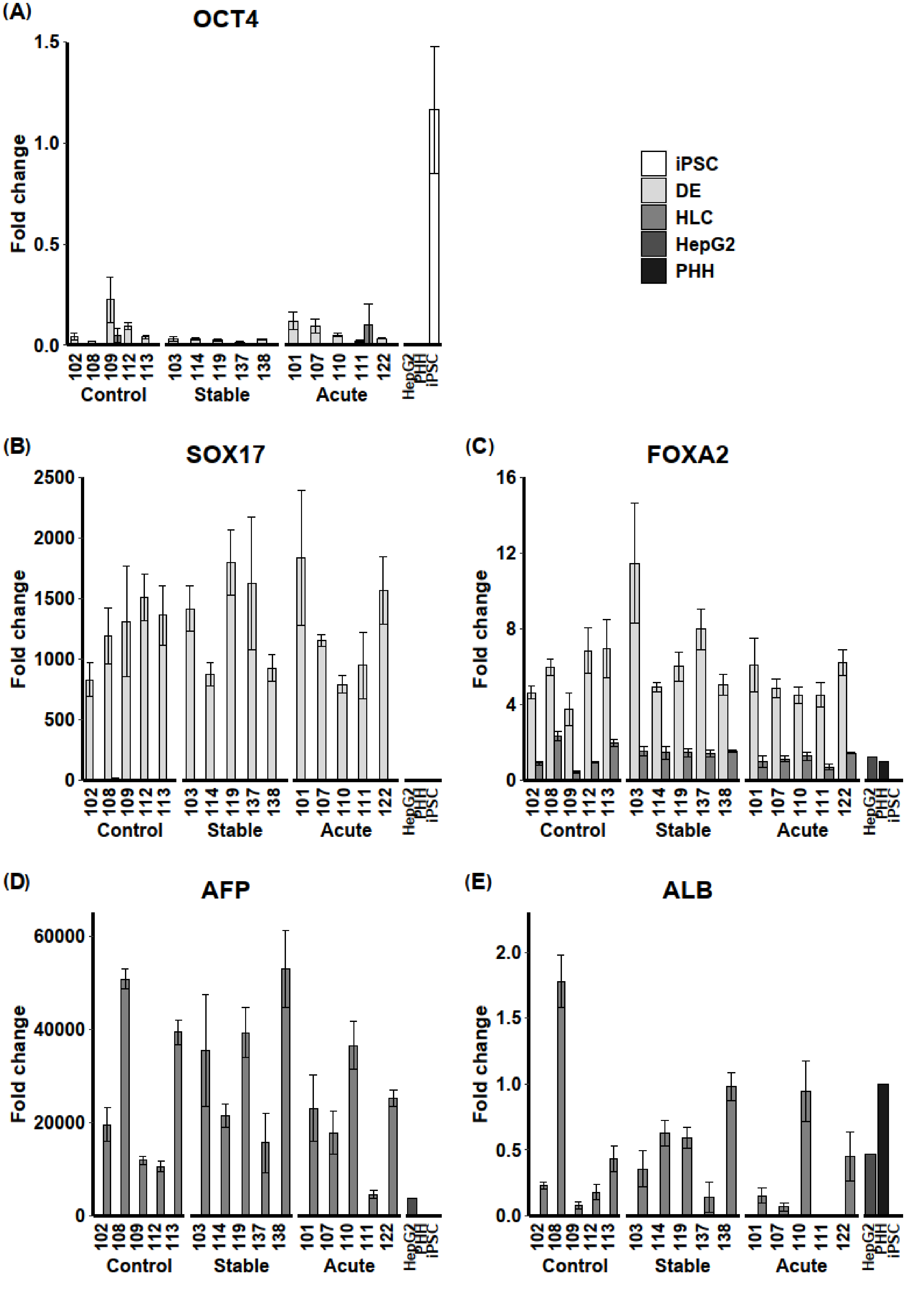

Figure S4. Gene expression analysis by RT-qPCR during hepatic differentiation. Average normalized expression of the patients' cell lines is presented. (A) Expression of pluripotency marker *OCT4*. (B-C) Expression of endoderm-specific markers *SOX17* and *FOXA2*. (D-E) Expression of hepatic markers *AFP* and *ALB*.

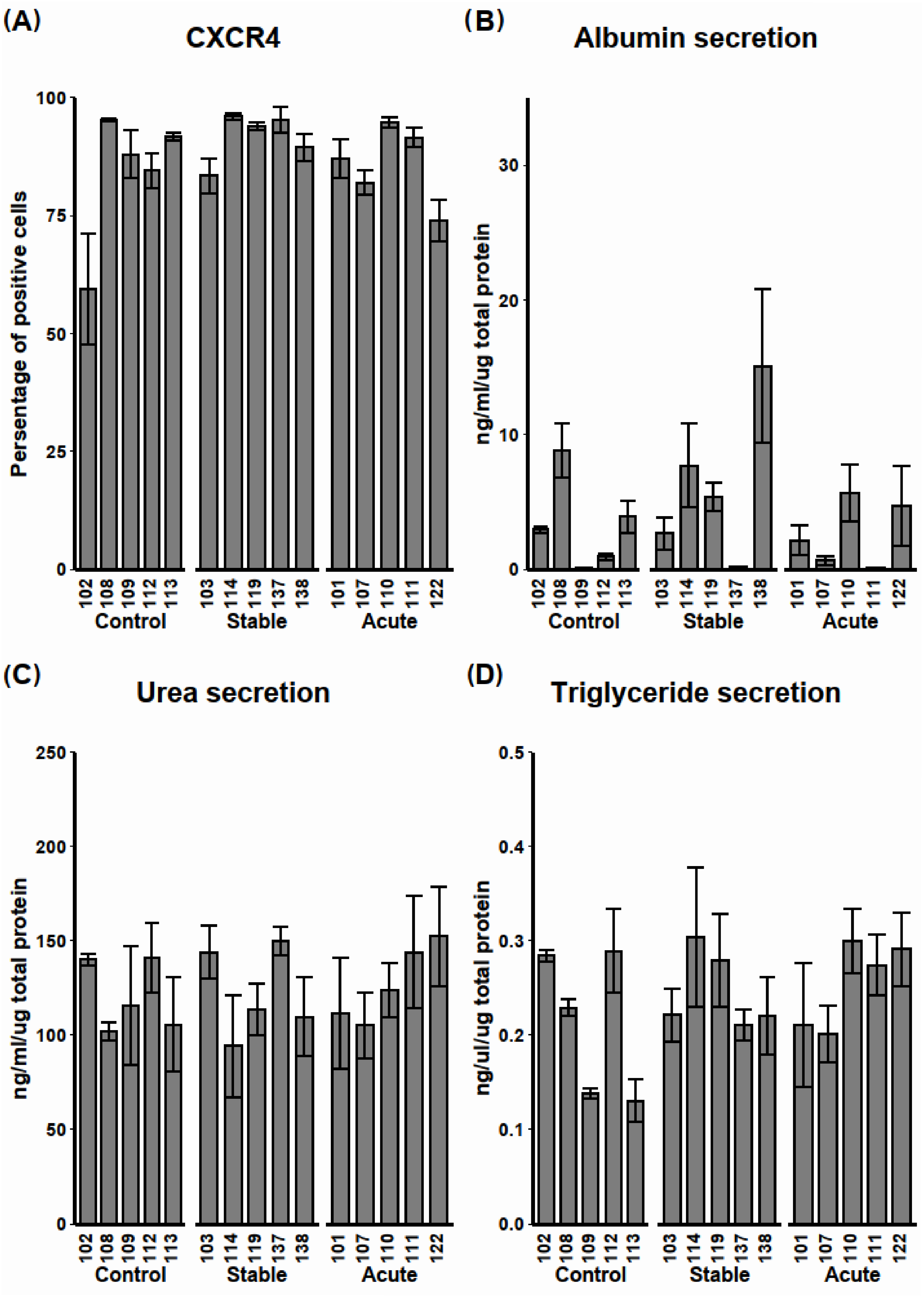

Figure S5. Characterization of the cells during differentiation process at protein level. Average of the patient's cell lines is presented. (A) Percentage of cells positive for CXCR4 expression at definitive endoderm stage detected by flow cytometry. (B) Secretion of albumin to conditioned medium (24h) by hepatocyte-like cells (HLCs). (C) Secretion of urea to conditioned medium (24h) by HLCs. (D) Triglyceride content of conditioned medium (24 h) at HLC stage.

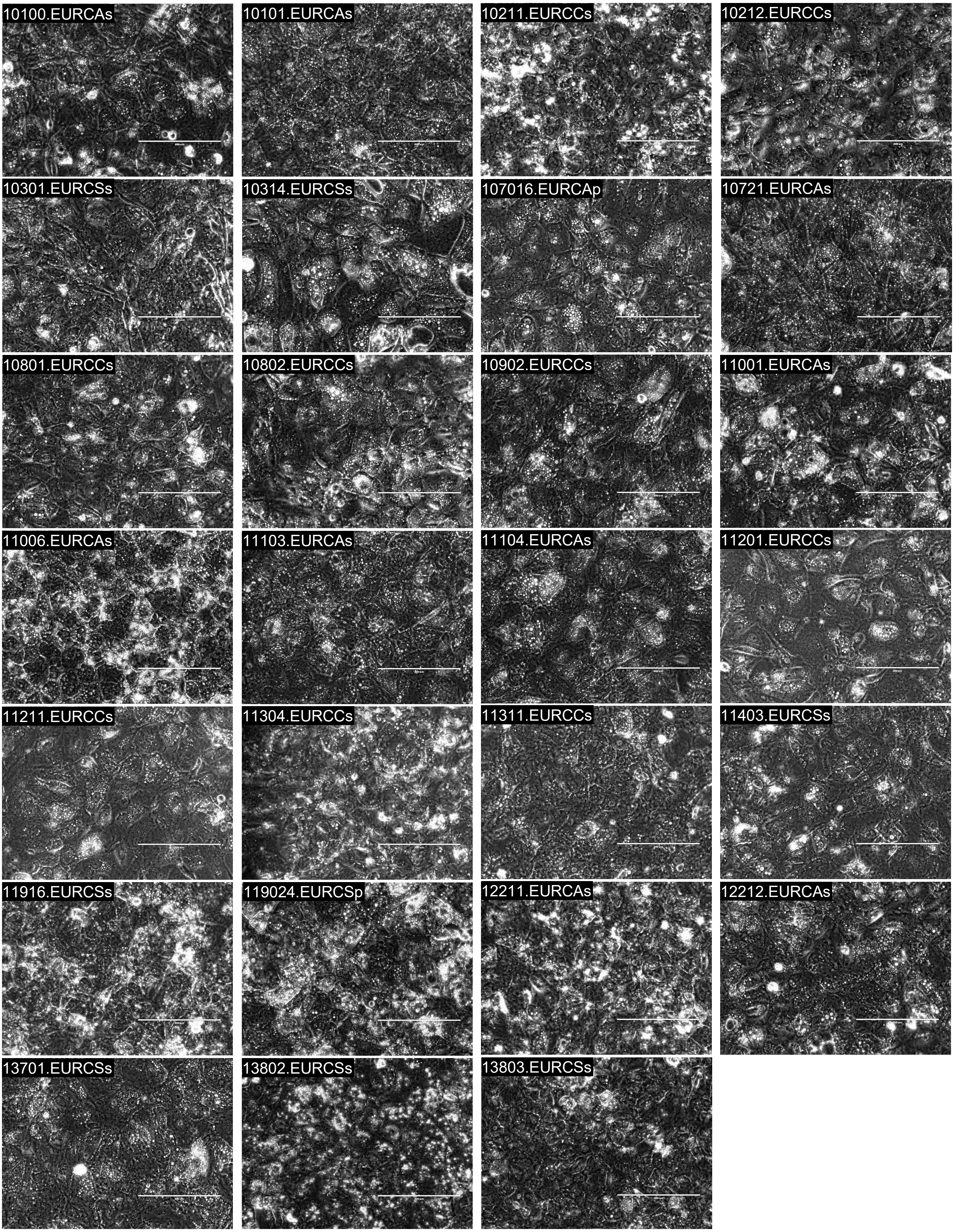

Figure S6. Hepatocyte-like cells (HLCs) at the end of differentiation. Scale bar 200 μm.

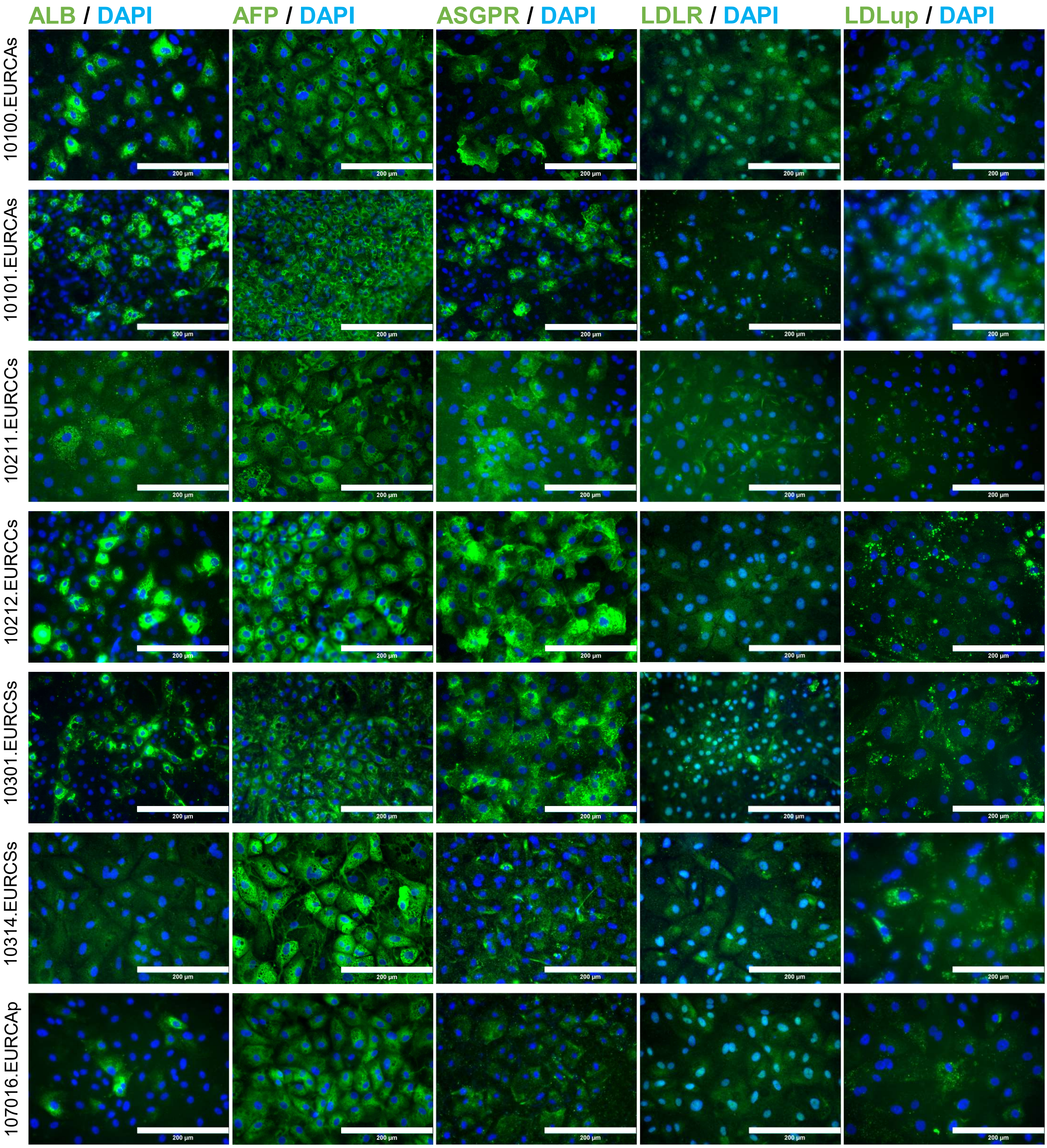

Figure S7. Characterization of HLCs with immunohistochemistry. LDLup – LDL uptake assay.

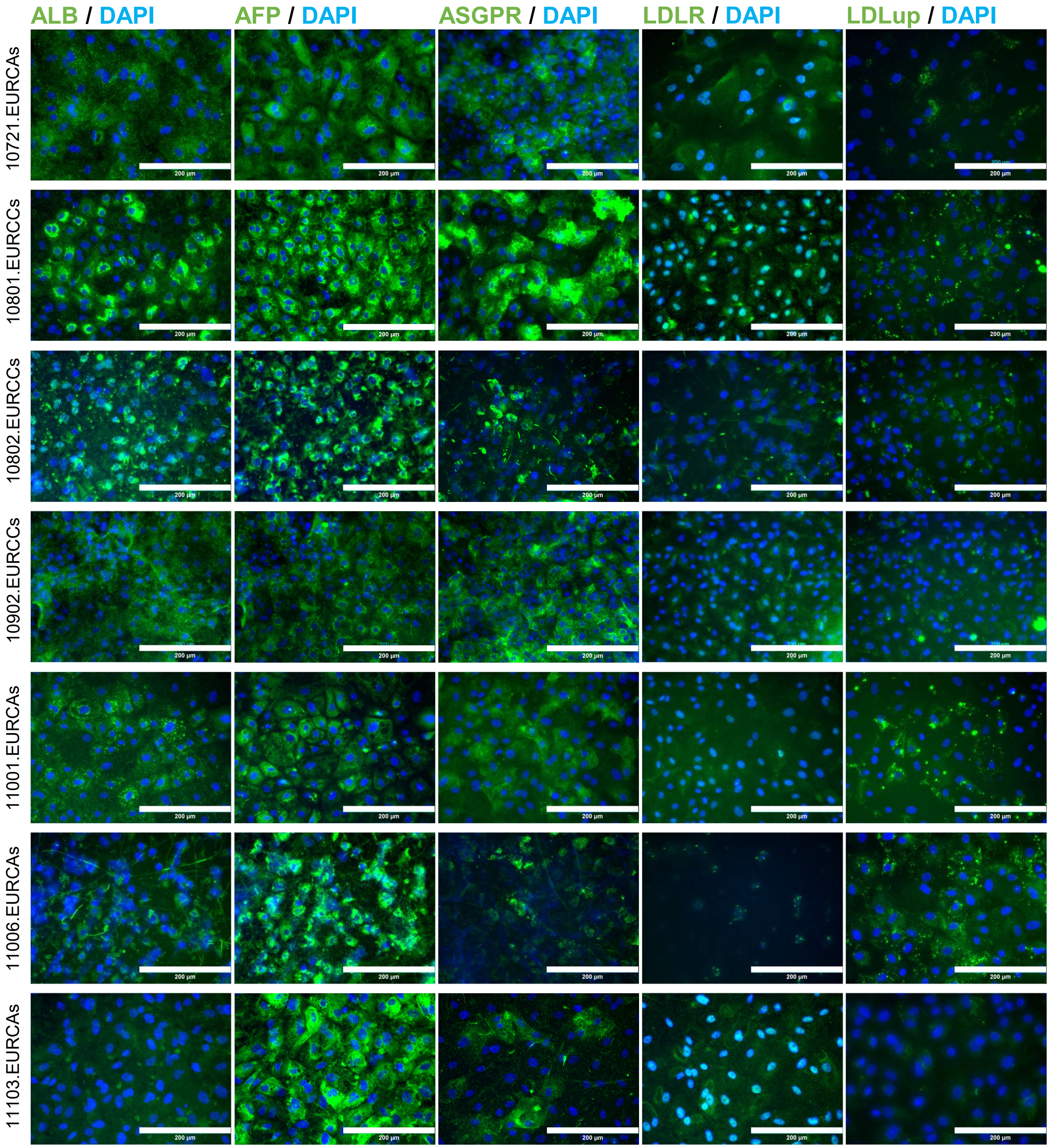

Figure S8. Characterization of HLCs with immunohistochemistry. LDLup – LDL uptake assay.

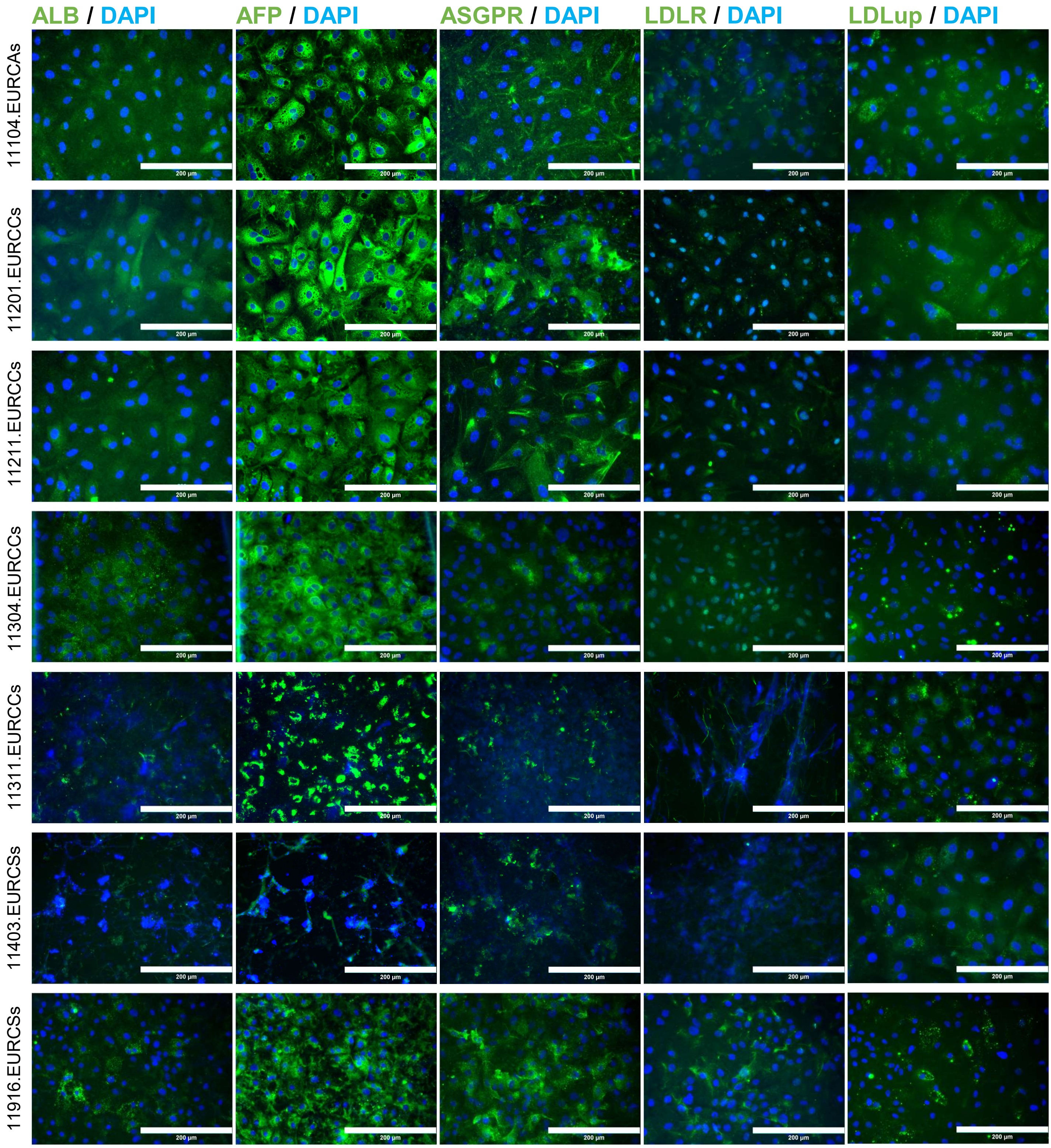

Figure S9. Characterization of HLCs with immunohistochemistry. LDLup – LDL uptake assay.

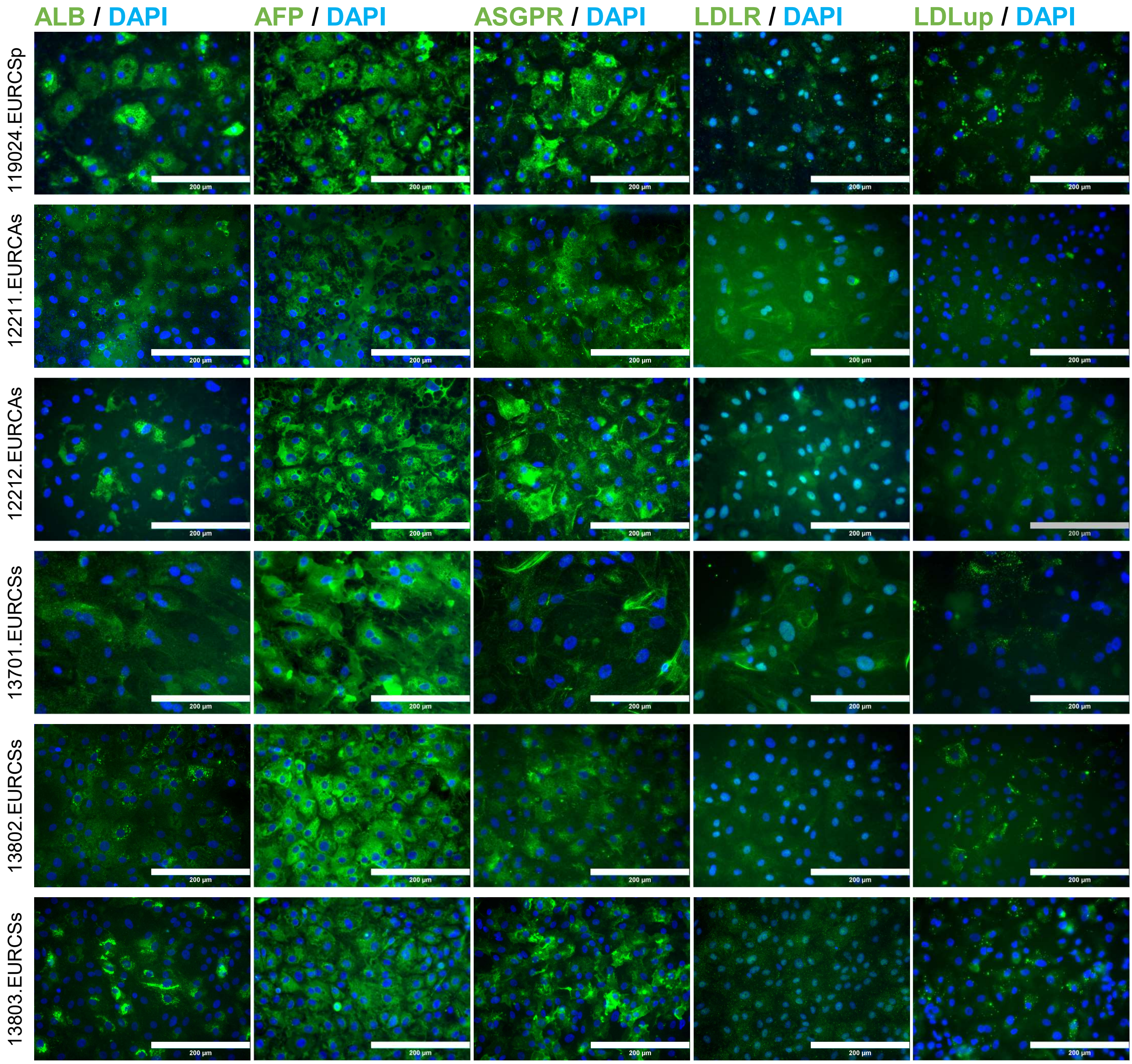

Figure S10. Characterization of HLCs with immunohistochemistry. LDLup – LDL uptake assay.

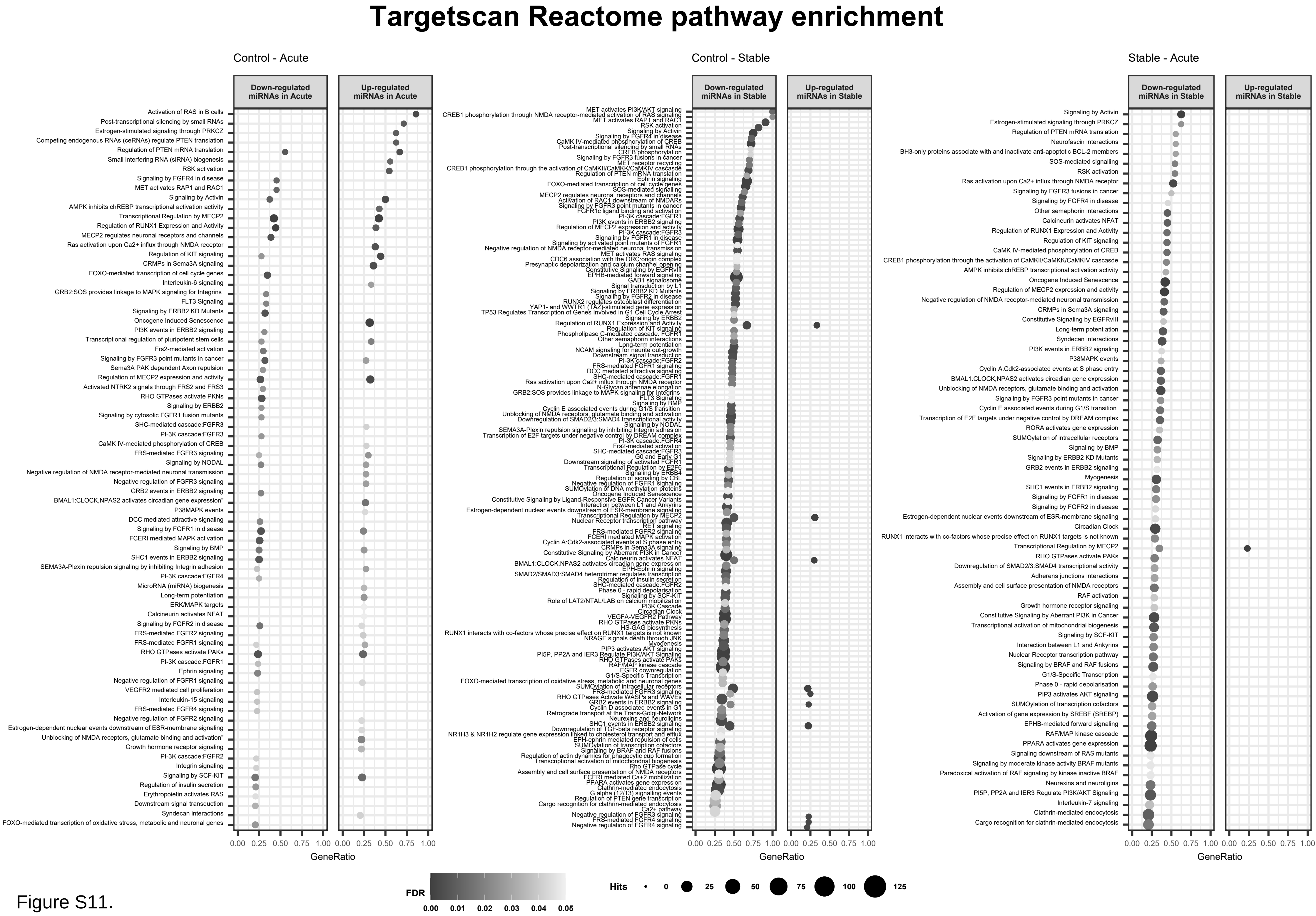
Targetscan Reactome pathway enrichment
Control - Acute
Control - Stable
Stable - Acute
Down-regulated miRNAs in Acute
Up-regulated miRNAs in Acute
Down-regulated miRNAs in Stable
Up-regulated miRNAs in Stable
Down-regulated miRNAs in Stable
Up-regulated miRNAs in Stable
GeneRatio
FDR
0.00 0.01 0.02 0.03 0.04 0.05
Hits
0 25 50 75 100 125

Figure S11.

Figure S11. Reactome pathway enrichment analysis of miRNA targets predicted with TarPmiR and Targetscan. Analysis of miRNAs either down- or up-regulated in the acute CAD patients compared to controls, miRNAs either down- or up-regulated in the stable CAD patients compared to the controls, miRNAs either down- or up-regulated in the stable CAD patients compared to the acute CAD patients. Only pathways that are statistically significant (FDR <0.5) and pathways with gene ratio >0.2 are presented. Bubble size represents the amount of hits within the pathway from the predicted target genes. Gene ratio represents the ratio of the predicted targets within a pathway and all genes in that pathway.

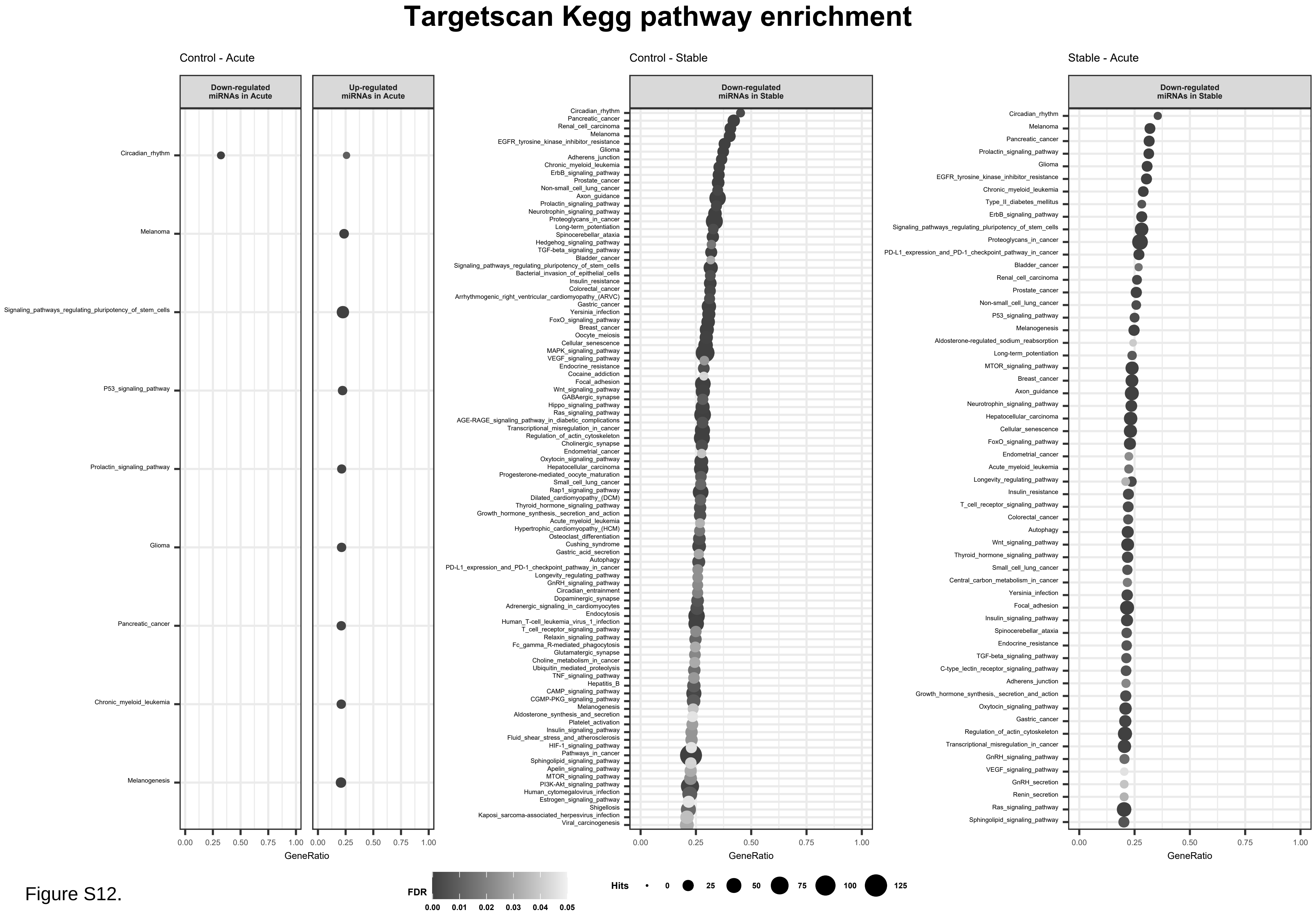
Targetscan Kegg pathway enrichment
Control - Acute
Down-regulated miRNAs in Acute
Up-regulated miRNAs in Acute
Circadian_rhythm
Melanoma
Signaling_pathways_regulating_pluripotency_of_stem_cells
P53_signaling_pathway
Prolactin_signaling_pathway
Glioma
Pancreatic_cancer
Chronic_myeloid_leukemia
Melanogenesis
GeneRatio
Control - Stable
Down-regulated miRNAs in Stable
Circadian_rhythm
Pancreatic_cancer
Renal_cell_carcinoma
Melanoma
EGFR_tyrosine_kinase_inhibitor_resistance
Glioma
Adherens_junction
Chronic_myeloid_leukemia
ErbB_signaling_pathway
Prostate_cancer
Non-small_cell_lung_cancer
Axon_guidance
Prolactin_signaling_pathway
Neurotrophin_signaling_pathway
Proteoglycans_in_cancer
Long-term_potentiation
Spinocerebellar_ataxia
Hedgehog_signaling_pathway
TGF-beta_signaling_pathway
Bladder_cancer
Signaling_pathways_regulating_pluripotency_of_stem_cells
Bacterial_invasion_of_epithelial_cells
Insulin_resistance
Colorectal_cancer
Arrhythmogenic_right_ventricular_cardiomyopathy_(ARVC)
Gastric_cancer
Yersinia_infection
FoxO_signaling_pathway
Breast_cancer
Oocyte_meiosis
Cellular_senescence
MAPK_signaling_pathway
VEGF_signaling_pathway
Endocrine_resistance
Cocaine_addiction
Focal_adhesion
Wnt_signaling_pathway
GABAergic_synapse
Hippo_signaling_pathway
Ras_signaling_pathway
AGE-RAGE_signaling_pathway_in_diabetic_complications
Transcriptional_misregulation_in_cancer
Regulation_of_actin_cytoskeleton
Cholinergic_synapse
Endometrial_cancer
Oxytocin_signaling_pathway
Hepatocellular_carcinoma
Progesterone-mediated_oocyte_maturation
Small_cell_lung_cancer
Rap1_signaling_pathway
Dilated_cardiomyopathy_(DCM)
Thyroid_hormone_signaling_pathway
Growth_hormone_synthesis,_secretion_and_action
Acute_myeloid_leukemia
Hypertrophic_cardiomyopathy_(HCM)
Osteoclast_differentiation
Cushing_syndrome
Gastric_acid_secretion
Autophagy
PD-L1_expression_and_PD-1_checkpoint_pathway_in_cancer
Longevity_regulating_pathway
GnRH_signaling_pathway
Circadian_entrainment
Dopaminergic_synapse
Adrenergic_signaling_in_cardiomyocytes
Endocytosis
Human_T-cell_leukemia_virus_1_infection
T_cell_receptor_signaling_pathway
Relaxin_signaling_pathway
Fc_gamma_R-mediated_phagocytosis
Glutamatergic_synapse
Choline_metabolism_in_cancer
Ubiquitin_mediated_proteolysis
TNF_signaling_pathway
Hepatitis_B
CAMP_signaling_pathway
CGMP-PKG_signaling_pathway
Melanogenesis
Aldosterone_synthesis_and_secretion
Platelet_activation
Insulin_signaling_pathway
Fluid_shear_stress_and_atherosclerosis
HIF-1_signaling_pathway
Pathways_in_cancer
Sphingolipid_signaling_pathway
Apelin_signaling_pathway
MTOR_signaling_pathway
PI3K-Akt_signaling_pathway
Human_cytomegalovirus_infection
Estrogen_signaling_pathway
Shigellosis
Kaposi_sarcoma-associated_herpesvirus_infection
Viral_carcinogenesis
GeneRatio
Stable - Acute
Down-regulated miRNAs in Stable
Circadian_rhythm
Melanoma
Pancreatic_cancer
Prolactin_signaling_pathway
Glioma
EGFR_tyrosine_kinase_inhibitor_resistance
Chronic_myeloid_leukemia
Type_II_diabetes_mellitus
ErbB_signaling_pathway
Signaling_pathways_regulating_pluripotency_of_stem_cells
Proteoglycans_in_cancer
PD-L1_expression_and_PD-1_checkpoint_pathway_in_cancer
Bladder_cancer
Renal_cell_carcinoma
Prostate_cancer
Non-small_cell_lung_cancer
P53_signaling_pathway
Melanogenesis
Aldosterone-regulated_sodium_reabsorption
Long-term_potentiation
MTOR_signaling_pathway
Breast_cancer
Axon_guidance
Neurotrophin_signaling_pathway
Hepatocellular_carcinoma
Cellular_senescence
FoxO_signaling_pathway
Endometrial_cancer
Acute_myeloid_leukemia
Longevity_regulating_pathway
Insulin_resistance
T_cell_receptor_signaling_pathway
Colorectal_cancer
Autophagy
Wnt_signaling_pathway
Thyroid_hormone_signaling_pathway
Small_cell_lung_cancer
Central_carbon_metabolism_in_cancer
Yersinia_infection
Focal_adhesion
Insulin_signaling_pathway
Spinocerebellar_ataxia
Endocrine_resistance
TGF-beta_signaling_pathway
C-type_lectin_receptor_signaling_pathway
Adherens_junction
Growth_hormone_synthesis,_secretion_and_action
Oxytocin_signaling_pathway
Gastric_cancer
Regulation_of_actin_cytoskeleton
Transcriptional_misregulation_in_cancer
GnRH_signaling_pathway
VEGF_signaling_pathway
GnRH_secretion
Renin_secretion
Ras_signaling_pathway
Sphingolipid_signaling_pathway
GeneRatio
0.00 0.25 0.50 0.75 1.00
FDR
0.00 0.01 0.02 0.03 0.04 0.05
Hits
0 25 50 75 100 125

Figure S12.

Figure S12. KEGG pathway enrichment analysis of miRNA targets predicted with TarPmiR and Targetscan. Analysis of miRNAs either down- or up-regulated in the acute CAD patients compared to controls, miRNAs either down- or up-regulated in the stable CAD patients compared to the controls, miRNAs either down- or up-regulated the stable CAD patients compared to the acute CAD patients. Only pathways that are statistically significant (FDR <0.5) and pathways with gene ratio >0.2 are presented. Bubble size represents the amount of hits within the pathway from the predicted target genes. Gene ratio represents the ratio of the predicted targets within a pathway and all genes in that pathway.

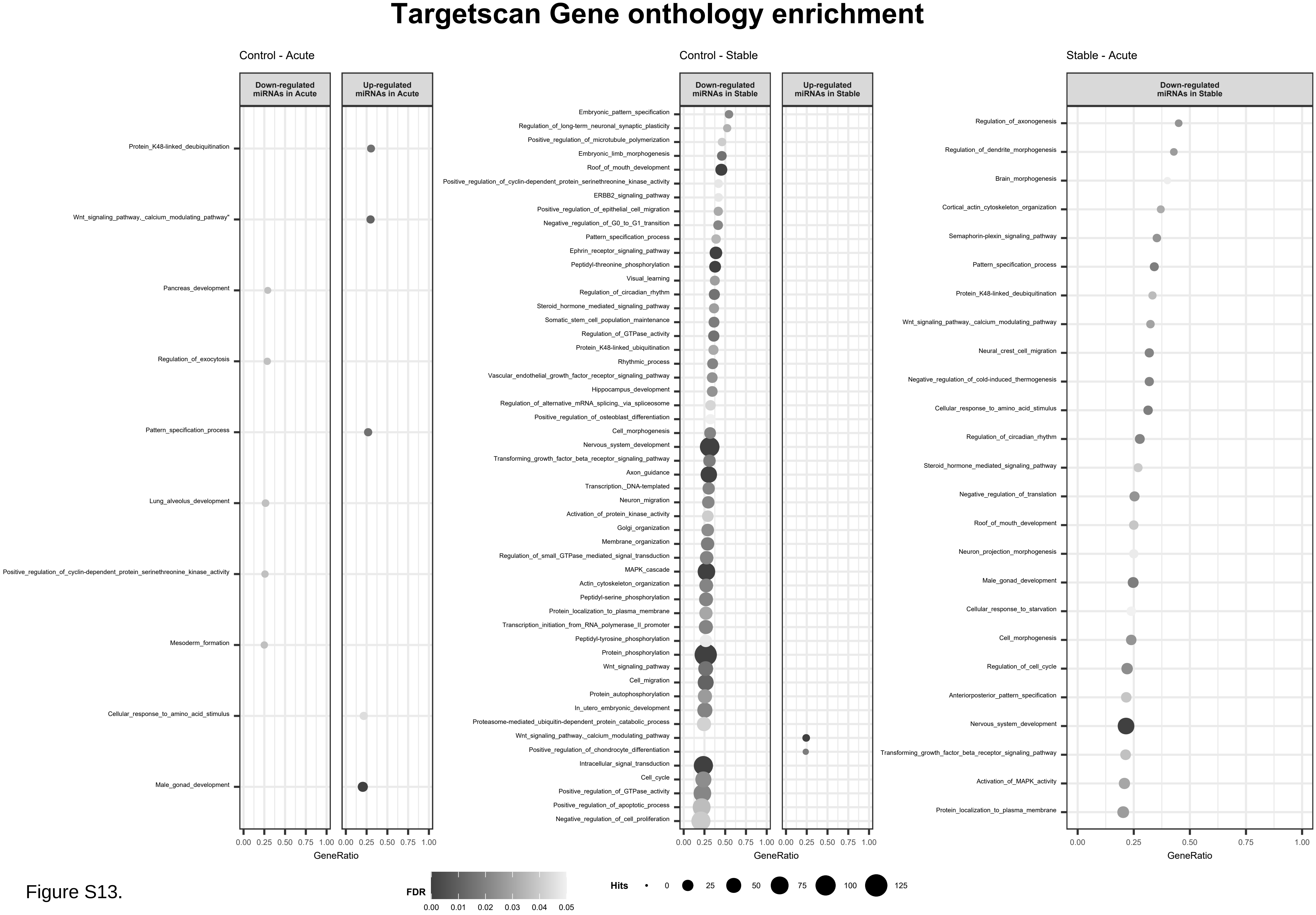
Targetscan Gene onthology enrichment
Control - Acute
Down-regulated miRNAs in Acute
Up-regulated miRNAs in Acute
Protein_K48-linked_deubiquitination
Wnt_signaling_pathway,_calcium_modulating_pathway"
Pancreas_development
Regulation_of_exocytosis
Pattern_specification_process
Lung_alveolus_development
Positive_regulation_of_cyclin-dependent_protein_serinethreonine_kinase_activity
Mesoderm_formation
Cellular_response_to_amino_acid_stimulus
Male_gonad_development
0.00 0.25 0.50 0.75 1.00
GeneRatio
Control - Stable
Down-regulated miRNAs in Stable
Up-regulated miRNAs in Stable
Embryonic_pattern_specification
Regulation_of_long-term_neuronal_synaptic_plasticity
Positive_regulation_of_microtubule_polymerization
Embryonic_limb_morphogenesis
Roof_of_mouth_development
Positive_regulation_of_cyclin-dependent_protein_serinethreonine_kinase_activity
ERBB2_signaling_pathway
Positive_regulation_of_epithelial_cell_migration
Negative_regulation_of_G0_to_G1_transition
Pattern_specification_process
Ephrin_receptor_signaling_pathway
Peptidyl-threonine_phosphorylation
Visual_learning
Regulation_of_circadian_rhythm
Steroid_hormone_mediated_signaling_pathway
Somatic_stem_cell_population_maintenance
Regulation_of_GTPase_activity
Protein_K48-linked_ubiquitination
Rhythmic_process
Vascular_endothelial_growth_factor_receptor_signaling_pathway
Hippocampus_development
Regulation_of_alternative_mRNA_splicing,_via_spliceosome
Positive_regulation_of_osteoblast_differentiation
Cell_morphogenesis
Nervous_system_development
Transforming_growth_factor_beta_receptor_signaling_pathway
Axon_guidance
Transcription,_DNA-templated
Neuron_migration
Activation_of_protein_kinase_activity
Golgi_organization
Membrane_organization
Regulation_of_small_GTPase_mediated_signal_transduction
MAPK_cascade
Actin_cytoskeleton_organization
Peptidyl-serine_phosphorylation
Protein_localization_to_plasma_membrane
Transcription_initiation_from_RNA_polymerase_II_promoter
Peptidyl-tyrosine_phosphorylation
Protein_phosphorylation
Wnt_signaling_pathway
Cell_migration
Protein_autophosphorylation
In_utero_embryonic_development
Proteasome-mediated_ubiquitin-dependent_protein_catabolic_process
Wnt_signaling_pathway,_calcium_modulating_pathway
Positive_regulation_of_chondrocyte_differentiation
Intracellular_signal_transduction
Cell_cycle
Positive_regulation_of_GTPase_activity
Positive_regulation_of_apoptotic_process
Negative_regulation_of_cell_proliferation
0.00 0.25 0.50 0.75 1.00
GeneRatio
Stable - Acute
Down-regulated miRNAs in Stable
Regulation_of_axonogenesis
Regulation_of_dendrite_morphogenesis
Brain_morphogenesis
Cortical_actin_cytoskeleton_organization
Semaphorin-plexin_signaling_pathway
Pattern_specification_process
Protein_K48-linked_deubiquitination
Wnt_signaling_pathway,_calcium_modulating_pathway
Neural_crest_cell_migration
Negative_regulation_of_cold-induced_thermogenesis
Cellular_response_to_amino_acid_stimulus
Regulation_of_circadian_rhythm
Steroid_hormone_mediated_signaling_pathway
Negative_regulation_of_translation
Roof_of_mouth_development
Neuron_projection_morphogenesis
Male_gonad_development
Cellular_response_to_starvation
Cell_morphogenesis
Regulation_of_cell_cycle
Anteriorposterior_pattern_specification
Nervous_system_development
Transforming_growth_factor_beta_receptor_signaling_pathway
Activation_of_MAPK_activity
Protein_localization_to_plasma_membrane
0.00 0.25 0.50 0.75 1.00
GeneRatio
FDR
0.00 0.01 0.02 0.03 0.04 0.05
Hits
0 25 50 75 100 125

Figure S13.

Figure S13. Gene onthology function enrichment analysis of miRNA targets predicted with TarPmiR and Targetscan. Analysis of miRNAs either down-regulated or up-regulated in the acute CAD patients compared to controls, miRNAs either down- or up-regulated in the stable CAD patients compared to the controls, miRNAs either down- or up-regulated in the stable CAD patients compared to the acute CAD patients. Only pathways that are statistically significant (FDR <0.5) and pathways with gene ratio >0.2 are presented. Bubble size represents the amount of hits within the pathway from the predicted target genes. Gene ratio represents the ratio of the predicted targets within a pathway and all genes in that pathway.

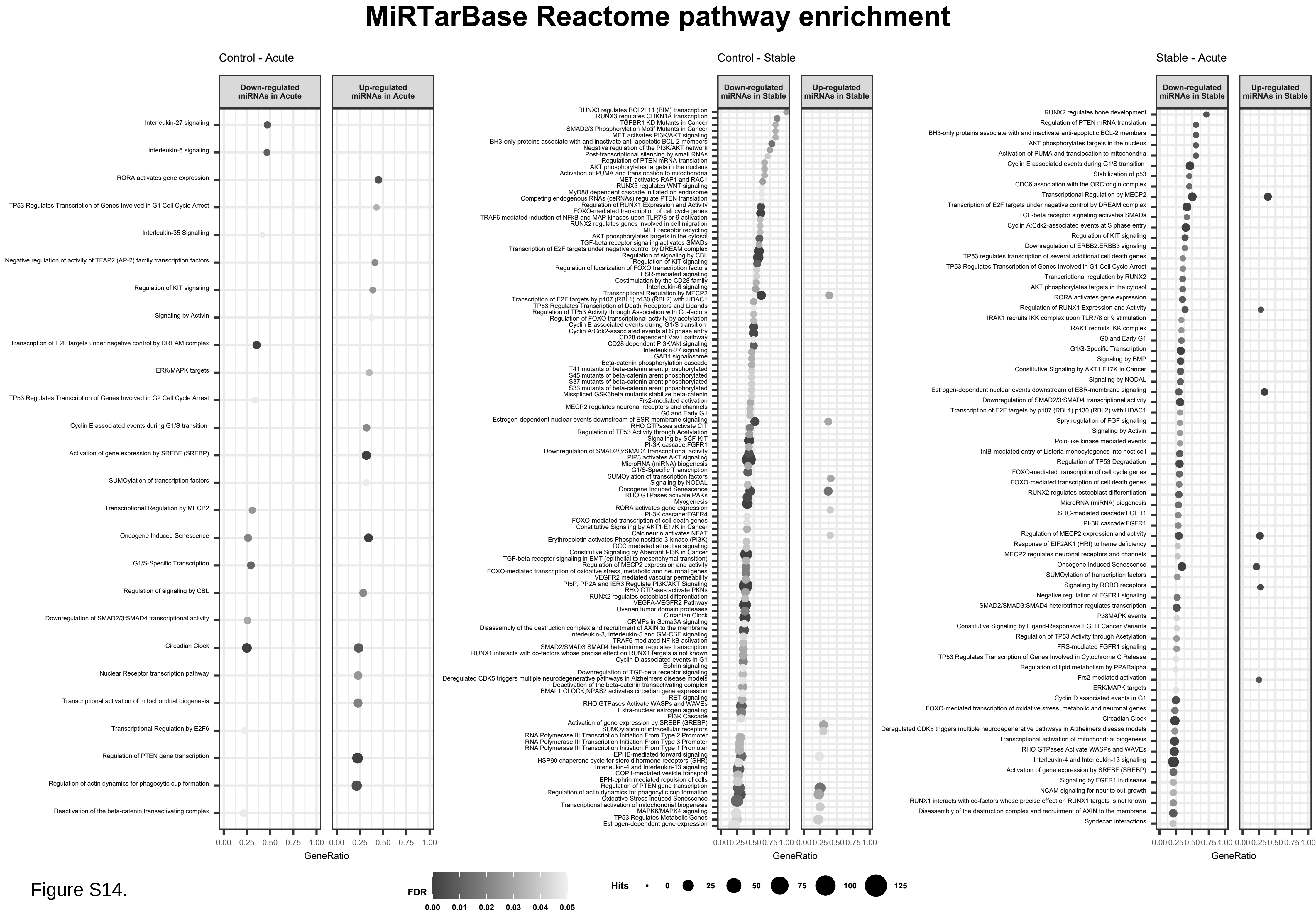

MiRTarBase Reactome pathway enrichment
Control - Acute
Control - Stable
Stable - Acute
Down-regulated miRNAs in Acute
Up-regulated miRNAs in Acute
Down-regulated miRNAs in Stable
Up-regulated miRNAs in Stable
Down-regulated miRNAs in Stable
Up-regulated miRNAs in Stable
Interleukin-27 signaling
Interleukin-6 signaling
RORA activates gene expression
TP53 Regulates Transcription of Genes Involved in G1 Cell Cycle Arrest
Interleukin-35 Signalling
Negative regulation of activity of TFAP2 (AP-2) family transcription factors
Regulation of KIT signaling
Signaling by Activin
Transcription of E2F targets under negative control by DREAM complex
ERK/MAPK targets
TP53 Regulates Transcription of Genes Involved in G2 Cell Cycle Arrest
Cyclin E associated events during G1/S transition
Activation of gene expression by SREBF (SREBP)
SUMOylation of transcription factors
Transcriptional Regulation by MECP2
Oncogene Induced Senescence
G1/S-Specific Transcription
Regulation of signaling by CBL
Downregulation of SMAD2/3:SMAD4 transcriptional activity
Circadian Clock
Nuclear Receptor transcription pathway
Transcriptional activation of mitochondrial biogenesis
Transcriptional Regulation by E2F6
Regulation of PTEN gene transcription
Regulation of actin dynamics for phagocytic cup formation
Deactivation of the beta-catenin transactivating complex
RUNX3 regulates BCL2L11 (BIM) transcription
RUNX3 regulates CDKN1A transcription
TGFBR1 KD Mutants in Cancer
SMAD2/3 Phosphorylation Motif Mutants in Cancer
MET activates PI3K/AKT signaling
BH3-only proteins associate with and inactivate anti-apoptotic BCL-2 members
Negative regulation of the PI3K/AKT network
Post-transcriptional silencing by small RNAs
Regulation of PTEN mRNA translation
AKT phosphorylates targets in the nucleus
Activation of PUMA and translocation to mitochondria
MET activates RAP1 and RAC1
RUNX3 regulates WNT signaling
MyD88 dependent cascade initiated on endosome
Competing endogenous RNAs (ceRNAs) regulate PTEN translation
Regulation of RUNX1 Expression and Activity
FOXO-mediated transcription of cell cycle genes
TRAF6 mediated induction of NFkB and MAP kinases upon TLR7/8 or 9 activation
RUNX2 regulates genes involved in cell migration
MET receptor recycling
AKT phosphorylates targets in the cytosol
TGF-beta receptor signaling activates SMADs
Transcription of E2F targets under negative control by DREAM complex
Regulation of signaling by CBL
Regulation of KIT signaling
Regulation of localization of FOXO transcription factors
ESR-mediated signaling
Costimulation by the CD28 family
Interleukin-6 signaling
Transcriptional Regulation by MECP2
Transcription of E2F targets by p107 (RBL1) p130 (RBL2) with HDAC1
TP53 Regulates Transcription of Death Receptors and Ligands
Regulation of TP53 Activity through Association with Co-factors
Regulation of FOXO transcriptional activity by acetylation
Cyclin E associated events during G1/S transition
Cyclin A:Cdk2-associated events at S phase entry
CD28 dependent Vav1 pathway
CD28 dependent PI3K/Akt signaling
Interleukin-27 signaling
GAB1 signalosome
Beta-catenin phosphorylation cascade
T41 mutants of beta-catenin arent phosphorylated
S45 mutants of beta-catenin arent phosphorylated
S37 mutants of beta-catenin arent phosphorylated
S33 mutants of beta-catenin arent phosphorylated
Misspliced GSK3beta mutants stabilize beta-catenin
Frs2-mediated activation
MECP2 regulates neuronal receptors and channels
G0 and Early G1
Estrogen-dependent nuclear events downstream of ESR-membrane signaling
RHO GTPases activate CIT
Regulation of TP53 Activity through Acetylation
Signaling by SCF-KIT
PI-3K cascade:FGFR1
Downregulation of SMAD2/3:SMAD4 transcriptional activity
PIP3 activates AKT signaling
MicroRNA (miRNA) biogenesis
G1/S-Specific Transcription
SUMOylation of transcription factors
Signaling by NODAL
Oncogene Induced Senescence
RHO GTPases activate PAKs
Myogenesis
RORA activates gene expression
PI-3K cascade:FGFR4
FOXO-mediated transcription of cell death genes
Constitutive Signaling by AKT1 E17K in Cancer
Calcineurin activates NFAT
Erythropoietin activates Phosphoinositide-3-kinase (PI3K)
DCC mediated attractive signaling
Constitutive Signaling by Aberrant PI3K in Cancer
TGF-beta receptor signaling in EMT (epithelial to mesenchymal transition)
Regulation of MECP2 expression and activity
FOXO-mediated transcription of oxidative stress, metabolic and neuronal genes
VEGFR2 mediated vascular permeability
PI5P, PP2A and IER3 Regulate PI3K/AKT Signaling
RHO GTPases activate PKNs
RUNX2 regulates osteoblast differentiation
VEGFA-VEGFR2 Pathway
Ovarian tumor domain proteases
Circadian Clock
CRMPs in Sema3A signaling
Disassembly of the destruction complex and recruitment of AXIN to the membrane
Interleukin-3, Interleukin-5 and GM-CSF signaling
TRAF6 mediated NF-kB activation
SMAD2/SMAD3:SMAD4 heterotrimer regulates transcription
RUNX1 interacts with co-factors whose precise effect on RUNX1 targets is not known
Cyclin D associated events in G1
Ephrin signaling
Downregulation of TGF-beta receptor signaling
Deregulated CDK5 triggers multiple neurodegenerative pathways in Alzheimers disease models
Deactivation of the beta-catenin transactivating complex
BMAL1:CLOCK,NPAS2 activates circadian gene expression
RET signaling
RHO GTPases Activate WASPs and WAVEs
Extra-nuclear estrogen signaling
PI3K Cascade
Activation of gene expression by SREBF (SREBP)
SUMOylation of intracellular receptors
RNA Polymerase III Transcription Initiation From Type 2 Promoter
RNA Polymerase III Transcription Initiation From Type 3 Promoter
RNA Polymerase III Transcription Initiation From Type 1 Promoter
EPHB-mediated forward signaling
HSP90 chaperone cycle for steroid hormone receptors (SHR)
Interleukin-4 and Interleukin-13 signaling
COPII-mediated vesicle transport
EPH-ephrin mediated repulsion of cells
Regulation of PTEN gene transcription
Regulation of actin dynamics for phagocytic cup formation
Oxidative Stress Induced Senescence
Transcriptional activation of mitochondrial biogenesis
MAPK6/MAPK4 signaling
TP53 Regulates Metabolic Genes
Estrogen-dependent gene expression
RUNX2 regulates bone development
Regulation of PTEN mRNA translation
BH3-only proteins associate with and inactivate anti-apoptotic BCL-2 members
AKT phosphorylates targets in the nucleus
Activation of PUMA and translocation to mitochondria
Cyclin E associated events during G1/S transition
Stabilization of p53
CDC6 association with the ORC:origin complex
Transcriptional Regulation by MECP2
Transcription of E2F targets under negative control by DREAM complex
TGF-beta receptor signaling activates SMADs
Cyclin A:Cdk2-associated events at S phase entry
Regulation of KIT signaling
Downregulation of ERBB2:ERBB3 signaling
TP53 regulates transcription of several additional cell death genes
TP53 Regulates Transcription of Genes Involved in G1 Cell Cycle Arrest
Transcriptional regulation by RUNX2
AKT phosphorylates targets in the cytosol
RORA activates gene expression
Regulation of RUNX1 Expression and Activity
IRAK1 recruits IKK complex upon TLR7/8 or 9 stimulation
IRAK1 recruits IKK complex
G0 and Early G1
G1/S-Specific Transcription
Signaling by BMP
Constitutive Signaling by AKT1 E17K in Cancer
Signaling by NODAL
Estrogen-dependent nuclear events downstream of ESR-membrane signaling
Downregulation of SMAD2/3:SMAD4 transcriptional activity
Transcription of E2F targets by p107 (RBL1) p130 (RBL2) with HDAC1
Spry regulation of FGF signaling
Signaling by Activin
Polo-like kinase mediated events
InlB-mediated entry of Listeria monocytogenes into host cell
Regulation of TP53 Degradation
FOXO-mediated transcription of cell cycle genes
FOXO-mediated transcription of cell death genes
RUNX2 regulates osteoblast differentiation
MicroRNA (miRNA) biogenesis
SHC-mediated cascade:FGFR1
PI-3K cascade:FGFR1
Regulation of MECP2 expression and activity
Response of EIF2AK1 (HRI) to heme deficiency
MECP2 regulates neuronal receptors and channels
Oncogene Induced Senescence
SUMOylation of transcription factors
Signaling by ROBO receptors
Negative regulation of FGFR1 signaling
SMAD2/SMAD3:SMAD4 heterotrimer regulates transcription
P38MAPK events
Constitutive Signaling by Ligand-Responsive EGFR Cancer Variants
Regulation of TP53 Activity through Acetylation
FRS-mediated FGFR1 signaling
TP53 Regulates Transcription of Genes Involved in Cytochrome C Release
Regulation of lipid metabolism by PPARalpha
Frs2-mediated activation
ERK/MAPK targets
Cyclin D associated events in G1
FOXO-mediated transcription of oxidative stress, metabolic and neuronal genes
Circadian Clock
Deregulated CDK5 triggers multiple neurodegenerative pathways in Alzheimers disease models
Transcriptional activation of mitochondrial biogenesis
RHO GTPases Activate WASPs and WAVEs
Interleukin-4 and Interleukin-13 signaling
Activation of gene expression by SREBF (SREBP)
Signaling by FGFR1 in disease
NCAM signaling for neurite out-growth
RUNX1 interacts with co-factors whose precise effect on RUNX1 targets is not known
Disassembly of the destruction complex and recruitment of AXIN to the membrane
Syndecan interactions
0.00 0.25 0.50 0.75 1.00
GeneRatio
FDR
0.00 0.01 0.02 0.03 0.04 0.05
Hits
0 25 50 75 100 125

Figure S14.

Figure S14. Reactome pathway enrichment analysis of miRNA targets predicted with TarPmiR and miRTarBase. Analysis of miRNAs either down- or up-regulated in the acute CAD patients compared to controls, miRNAs either down- or up-regulated in the stable CAD patients compared to the controls, miRNAs either down- or up-regulated the stable CAD patients compared to the acute CAD patients. Only pathways that are statistically significant (FDR <0.5) and pathways with gene ratio >0.2 are presented. Bubble size represents the amount of hits within the pathway from the predicted target genes. Gene ratio represents the ratio of the predicted targets within a pathway and all genes in that pathway.

MiRTarBase Kegg pathway enrichment

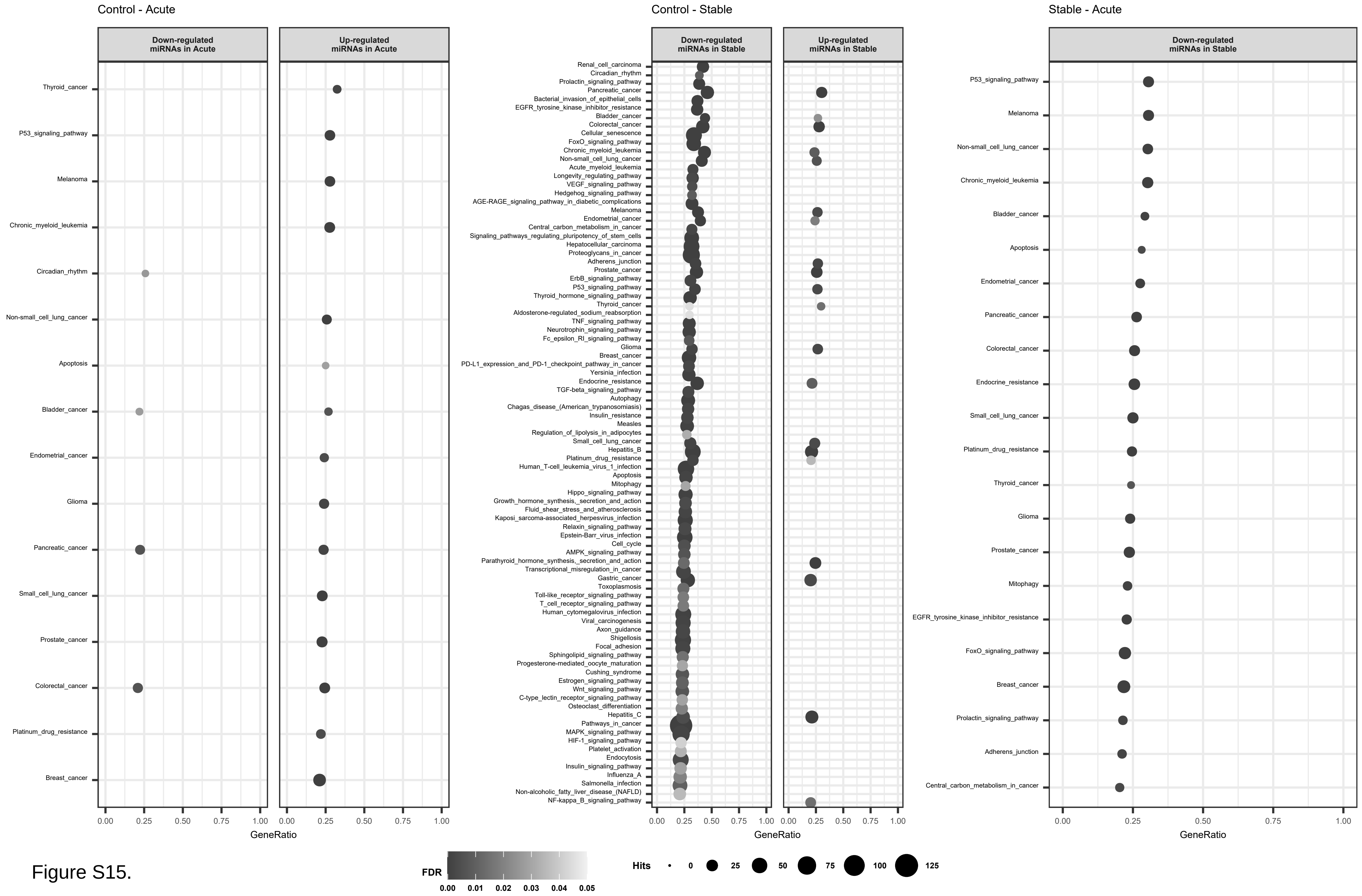

Figure S15.

Figure S15. KEGG pathway enrichment analysis of miRNA targets predicted with TarPmiR and miRTarBase. Analysis of miRNAs either down- or up-regulated in the acute CAD patients compared to controls, miRNAs either down- or up-regulated in the stable CAD patients compared to the controls, miRNAs either down- or up-regulated in the stable CAD patients compared to the acute CAD patients. Only pathways that are statistically significant (FDR <0.5) and pathways with gene ratio >0.2 are presented. Bubble size represents the amount of hits within the pathway from the predicted target genes. Gene ratio represents the ratio of the predicted targets within a pathway and all genes in that pathway.

MiRTarBase Gene Onthology enrichment

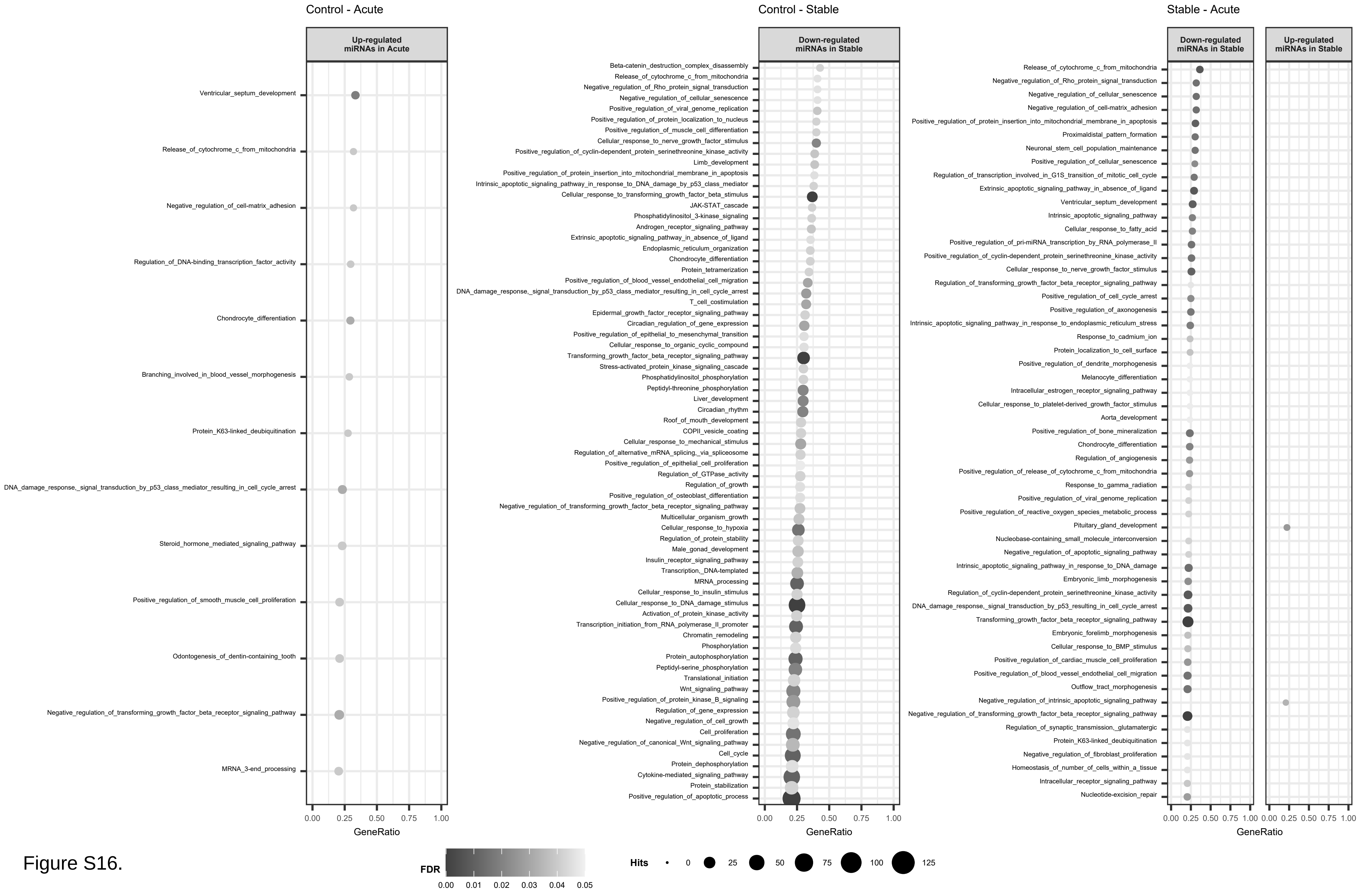

Figure S16.

Figure S16. Gene onthology function enrichment analysis of miRNA targets predicted with TarPmiR and miRTarBase. Analysis of miRNAs either down- or up-regulated in the acute CAD patients compared to controls, miRNAs either down- or up-regulated in the stable CAD patients compared to the controls, miRNAs either down- or up-regulated in the stable CAD patients compared to the acute CAD patients. Only pathways that are statistically significant (FDR< 0.5) and pathways with gene ratio >0.2 are presented. Bubble size represents the amount of hits within the pathway from the predicted target genes. Gene ratio represents the ratio of the predicted targets within a pathway and all genes in that pathway.

Supplementary Tables

Table S1. The induced pluripotent stem cell (iPSC) lines used in the study. The last letter at the end of the cell line name refers to the iPSC reprogramming method: s stands for Sendai reprogramming and p for plasmid reprogramming. A reference is added after the cell line if it has been characterized before.

Group	Patient	Age at biopsy	Family history	Cell line 1	Cell line 2
Acute	101	57	N.A.	UTA.10100.EURCAs [1]	UTA.10101.EURCAs
Acute	107	54	-	UTA.107016.EURCAp [2]	UTA.10721.EURCAs
Acute	110	53	+	UTA.11001.EURCAs,	UTA.11006.EURCAs
Acute	111	41	+	UTA.11103.EURCAs	UTA.11104.EURCAs
Acute	122	37	+	UTA.12211.EURCAs	UTA.12212.EURCAs
Control	102	53	N.A.	UTA.10211.EURCCs	UTA.10212.EURCCs [3]
Control	108	72	N.A.	UTA.10801.EURCCs [3]	UTA.10802.EURCCs [3]
Control	109	78	N.A.	UTA.10901.EURCCs*	UTA.10902.EURCCs [3]
Control	112	78	-	UTA.11201.EURCCs	UTA.11211.EURCCs
Control	113	70	-	UTA.11304.EURCCs [1]	UTA.11311.EURCCs [4]
Stable	103	61	+	UTA.10301.EURCSs	UTA.10314.EURCSs
Stable	114	75	+	UTA.11401.EURCSs* [1]	UTA.11403.EURCSs
Stable	119	54	N.A.	UTA.11916.EURCSs [2]	UTA.119024.EURCSp [2]
Stable	137	62	+	UTA.13701.EURCSs	UTA.137062.EURCSp [2]*
Stable	138	86	N.A.	UTA.13802.EURCSs	UTA.13803.EURCSs

**Did not differentiate from iPSC to HLC.*

[1] M. Kiamehr, L.E. Viiri, T. Vihervaara, K.M. Koistinen, M. Hilvo, K. Ekroos, R. Käkelä, K. Aalto-Setälä, Lipidomic profiling of patient-specific iPSC-derived hepatocyte-like cells., Dis. Model. Mech. 10 (2017) 1141–1153. https://doi.org/10.1242/dmm.030841.

[2] S. Manzini, L.E. Viiri, S. Marttila, K. Aalto-Setälä, A Comparative View on Easy to Deploy non-Integrating Methods for Patient-Specific iPSC Production, Stem Cell Rev. Reports. 11 (2015) 900–908.

https://doi.org/10.1007/s12015-015-9619-3.

[3] M. Kiamehr, A. Klettner, E. Richert, A. Koskela, A. Koistinen, H. Skottman, K. Kaarniranta, K. Aalto-Setälä, K. Juuti-Uusitalo, Compromised barrier function in human induced pluripotent stem-cell-derived retinal pigment epithelial cells from type 2 diabetic patients, Int. J. Mol. Sci. 20 (2019). https://doi.org/10.3390/ijms20153773.

[4] M. Häkli, Jäntti S, Joki T, Sukki L, Tornberg K, Aalto-Setälä K, Kallio P, Pekkanen-Mattila M, Narkilahti S. Electrophysiological changes of human induced pluripotent stem cell-derived cardiomyocytes during acute hypoxia and reoxygenation, Stem Cells Int. 23 (2022) 3148. https://doi.org/10.3390/ijms23063148.

Table S2. Antibodies used in the study. Superscripts indicate secondary antibody used.

**Antibody**	**Animal**	**Dilution**	**Manufacturer**	**Catalogue number**
CXCR4	Mouse anti human	1:68	R&D Systems	FAB172P
OCT4[1 or 6]	Goat anti human	1:400	R&D Systems	AF1759
SOX17[2 or 4]	Mouse anti human	1:200	R&D Systems	MAB1924
FOXA2[1]	Goat anti human	1:200	Abcam	ab5074
AFP[5]	Rabbit anti human	1:500	DAKO	A000829
ALB[2]	Mouse anti human	1:400	Abcam	ab10241
ALB[2]	Mouse anti human	1:400	R&D Systems	MAB1455
ASGPR1[2]	Mouse anti human	1:100	Novus Biologicals	NBP1-60150
LDLR[3]	Rabbit anti human	1:200	Cayman Chemical	10012422
**Secondary antibodies**				
1 Alexa Fluor 488	Donkey anti goat	1:600	Life Technologies	A11055
2 Alexa Fluor 488	Donkey anti mouse	1:600	Life Technologies	A21202
3 Alexa Fluor 488	Donkey anti rabbit	1:600	Life Technologies	A21206
4 Alexa Fluor 568	Donkey anti mouse	1:600	Life Technologies	A10037
5 Alexa Fluor 568	Donkey anti rabbit	1:600	Life Technologies	A10042
6 Alexa Fluor 568	Donkey anti goat	1:600	Life Technologies	A11057

Table S3. The miRNA assays and assay ID numbers (Applied Biosystems) used for miRNA validation by real-time quantitative PCR .

miRNA	Assay ID
hsa-miR-16-5p	477860_mir
hsa-miR-423-3p	478327_mir
hsa-miR-302c-3p	478509_mir
hsa-miR-1263	478669_mir
hsa-miR-122-5p	477855_mir
hsa-miR-6869-5p	480491_mir
hsa-miR-6727-5p	480235_mir
hsa-miR-149-5p	477917_mir
hsa-miR-92a-3p	477827_mir
hsa-miR-221-3p	477981_mir

Table S4. Lists used for miRNA target prediction.

List 1. Control-Acute, downregulated in Acute

	logFC	P-value	FDR
hsa-miR-574-3p	0.88	5.94E-04	1.29E-01
hsa-miR-92a-3p	0.59	4.11E-03	3.17E-01
hsa-miR-933	0.49	6.13E-03	3.17E-01
hsa-miR-93-5p	0.44	6.39E-03	3.17E-01
hsa-miR-210-3p	0.92	7.14E-03	3.17E-01
hsa-miR-1271-5p	0.54	1.22E-02	3.47E-01
hsa-miR-8485	0.69	1.22E-02	3.47E-01
hsa-miR-6858-3p	0.41	1.93E-02	3.80E-01
hsa-miR-4530	0.81	2.26E-02	4.10E-01
hsa-miR-129-2-3p	0.43	2.71E-02	4.19E-01
hsa-miR-766-3p	0.48	2.74E-02	4.19E-01
hsa-miR-8069	0.34	3.59E-02	4.83E-01
hsa-miR-149-5p	0.50	3.62E-02	4.83E-01
hsa-miR-33b-3p	0.47	3.72E-02	4.83E-01
hsa-miR-193a-3p	0.60	4.94E-02	5.12E-01
hsa-miR-1263	expressed in Control not in Acute		
hsa-miR-192-3p	expressed in Control not in Acute		
hsa-miR-486-5p	expressed in Control not in Acute		
hsa-miR-500a-5p	expressed in Control not in Acute		
hsa-miR-517a-3p	expressed in Control not in Acute		
hsa-miR-517c-3p	expressed in Control not in Acute		
hsa-miR-629-5p	expressed in Control not in Acute		

List 2. Control-Acute, upregulated in Acute

	logFC	P-value	FDR
hsa-miR-424-5p	-1.55	8.80E-03	3.17E-01
hsa-miR-6869-5p	-1.24	2.25E-05	9.73E-03
hsa-miR-150-3p	-1.10	8.35E-03	3.17E-01
hsa-miR-4327	-1.08	1.19E-02	3.47E-01
hsa-miR-3663-3p	-1.06	1.56E-02	3.63E-01
hsa-miR-503-5p	-1.03	1.59E-02	3.63E-01
hsa-miR-6821-5p	-0.81	1.92E-02	3.80E-01
hsa-miR-4497	-0.75	7.53E-03	3.17E-01
hsa-miR-5001-5p	-0.69	2.41E-02	4.17E-01
hsa-miR-4463	-0.67	1.33E-03	1.92E-01
hsa-miR-6850-5p	-0.65	5.30E-03	3.17E-01
hsa-miR-937-5p	-0.63	3.79E-02	4.83E-01
hsa-miR-6800-5p	-0.60	1.28E-02	3.47E-01
hsa-miR-4271	-0.60	8.76E-03	3.17E-01
hsa-miR-6727-5p	-0.58	1.42E-02	3.62E-01
hsa-miR-4741	-0.55	2.92E-02	4.21E-01
hsa-miR-4689	-0.48	2.27E-02	4.10E-01
hsa-miR-6756-5p	-0.45	1.82E-02	3.80E-01

hsa-miR-6068	-0.43	2.80E-02	4.19E-01
hsa-miR-638	-0.40	2.77E-02	4.19E-01
hsa-let-7c-5p	expressed in Acute not in Control		
hsa-let-7d-5p	expressed in Acute not in Control		
hsa-miR-100-5p	expressed in Acute not in Control		
hsa-miR-1227-5p	expressed in Acute not in Control		
hsa-miR-1229-3p	expressed in Acute not in Control		
hsa-miR-1273f	expressed in Acute not in Control		
hsa-miR-143-3p	expressed in Acute not in Control		
hsa-miR-181b-5p	expressed in Acute not in Control		
hsa-miR-187-5p	expressed in Acute not in Control		
hsa-miR-1914-3p	expressed in Acute not in Control		
hsa-miR-199b-5p	expressed in Acute not in Control		
hsa-miR-214-3p	expressed in Acute not in Control		
hsa-miR-221-5p	expressed in Acute not in Control		
hsa-miR-224-5p	expressed in Acute not in Control		
hsa-miR-3188	expressed in Acute not in Control		
hsa-miR-34a-3p	expressed in Acute not in Control		
hsa-miR-3679-3p	expressed in Acute not in Control		
hsa-miR-4274	expressed in Acute not in Control		
hsa-miR-4652-3p	expressed in Acute not in Control		
hsa-miR-4697-5p	expressed in Acute not in Control		
hsa-miR-4716-3p	expressed in Acute not in Control		
hsa-miR-542-5p	expressed in Acute not in Control		
hsa-miR-551b-3p	expressed in Acute not in Control		
hsa-miR-6722-3p	expressed in Acute not in Control		
hsa-miR-6731-3p	expressed in Acute not in Control		
hsa-miR-6753-3p	expressed in Acute not in Control		
hsa-miR-6757-3p	expressed in Acute not in Control		
hsa-miR-6765-3p	expressed in Acute not in Control		
hsa-miR-6785-3p	expressed in Acute not in Control		
hsa-miR-6794-5p	expressed in Acute not in Control		
hsa-miR-6795-3p	expressed in Acute not in Control		
hsa-miR-6806-5p	expressed in Acute not in Control		
hsa-miR-6824-5p	expressed in Acute not in Control		
hsa-miR-7845-5p	expressed in Acute not in Control		
hsa-miR-92b-3p	expressed in Acute not in Control		
hsa-miR-98-3p	expressed in Acute not in Control		

List 3. Control-Stable, downregulated in Stable

	logFC	P-value	FDR
hsa-miR-4484	0.28	4.54E-02	2.42E-01
hsa-miR-3180-5p	0.30	1.46E-02	1.51E-01
hsa-miR-6870-3p	0.31	2.66E-02	1.86E-01
hsa-miR-933	0.42	6.42E-03	9.58E-02
hsa-miR-484	0.43	4.04E-02	2.32E-01

hsa-miR-320a	0.43	7.86E-03	1.03E-01
hsa-miR-106b-5p	0.46	4.70E-02	2.45E-01
hsa-miR-125a-5p	0.47	2.39E-02	1.86E-01
hsa-miR-361-5p	0.49	5.27E-03	9.16E-02
hsa-miR-129-2-3p	0.50	4.54E-03	9.16E-02
hsa-miR-93-5p	0.51	6.51E-04	2.67E-02
hsa-miR-23a-3p	0.53	4.58E-02	2.42E-01
hsa-miR-193a-5p	0.55	1.83E-02	1.72E-01
hsa-miR-367-3p	0.56	3.21E-02	2.05E-01
hsa-miR-744-5p	0.56	1.01E-02	1.28E-01
hsa-miR-4428	0.57	4.33E-02	2.42E-01
hsa-miR-193a-3p	0.59	2.55E-02	1.86E-01
hsa-miR-652-3p	0.62	1.23E-02	1.38E-01
hsa-miR-92a-3p	0.63	6.72E-04	2.67E-02
hsa-miR-494-3p	0.64	4.49E-02	2.42E-01
hsa-miR-1271-5p	0.65	1.18E-03	3.80E-02
hsa-miR-19b-3p	0.67	4.42E-02	2.42E-01
hsa-miR-27a-3p	0.68	2.63E-02	1.86E-01
hsa-miR-7641	0.69	3.01E-02	2.00E-01
hsa-miR-6798-5p	0.72	3.74E-02	2.28E-01
hsa-miR-8485	0.74	2.61E-03	6.66E-02
hsa-miR-149-5p	0.80	5.94E-04	2.67E-02
hsa-miR-205-5p	0.80	4.97E-02	2.47E-01
hsa-miR-17-5p	0.80	5.76E-03	9.23E-02
hsa-miR-221-3p	0.81	2.01E-02	1.75E-01
hsa-miR-1260a	0.81	3.05E-02	2.00E-01
hsa-miR-130a-3p	0.82	4.92E-03	9.16E-02
hsa-miR-30a-3p	0.82	2.55E-02	1.86E-01
hsa-miR-20a-5p	0.86	1.36E-02	1.44E-01
hsa-miR-130b-3p	0.89	1.22E-02	1.38E-01
hsa-miR-19a-3p	0.89	3.33E-03	7.59E-02
hsa-miR-126-3p	0.95	4.44E-02	2.42E-01
hsa-miR-574-3p	0.95	6.27E-05	1.36E-02
hsa-miR-18a-5p	0.96	5.54E-03	9.23E-02
hsa-miR-145-5p	1.91	3.37E-02	2.11E-01
hsa-miR-10a-5p	expressed in Control not in Stable		
hsa-miR-10b-5p	expressed in Control not in Stable		
hsa-miR-196b-5p	expressed in Control not in Stable		
hsa-miR-199a-3p	expressed in Control not in Stable		
hsa-miR-199a-5p	expressed in Control not in Stable		
hsa-miR-3151-3p	expressed in Control not in Stable		
hsa-miR-326	expressed in Control not in Stable		
hsa-miR-486-5p	expressed in Control not in Stable		
hsa-miR-489-3p	expressed in Control not in Stable		
hsa-miR-517a-3p	expressed in Control not in Stable		
hsa-miR-517c-3p	expressed in Control not in Stable		

hsa-miR-522-3p	expressed in Control not in Stable
hsa-miR-563	expressed in Control not in Stable
hsa-miR-629-5p	expressed in Control not in Stable
hsa-miR-634	expressed in Control not in Stable
hsa-miR-6759-3p	expressed in Control not in Stable

List 4. Control-Stable, upregulated in Stable

	logFC	P-value	FDR
hsa-miR-6869-5p	-1.34	1.40E-06	6.07E-04
hsa-miR-150-3p	-1.06	3.56E-03	7.72E-02
hsa-miR-4327	-1.03	5.99E-03	9.26E-02
hsa-miR-6821-5p	-1.02	1.53E-03	4.41E-02
hsa-miR-4497	-0.90	5.35E-04	2.67E-02
hsa-miR-6727-5p	-0.86	1.99E-04	2.67E-02
hsa-miR-937-5p	-0.83	3.07E-03	7.39E-02
hsa-miR-4532	-0.81	2.64E-02	1.86E-01
hsa-miR-6850-5p	-0.79	3.17E-04	2.67E-02
hsa-miR-762	-0.79	1.05E-02	1.30E-01
hsa-miR-4741	-0.77	1.23E-03	3.80E-02
hsa-miR-3195	-0.74	6.78E-04	2.67E-02
hsa-miR-6800-5p	-0.73	1.09E-03	3.80E-02
hsa-miR-3663-3p	-0.72	4.90E-02	2.47E-01
hsa-miR-4689	-0.70	6.30E-04	2.67E-02
hsa-miR-874-3p	-0.68	7.73E-03	1.03E-01
hsa-miR-4463	-0.63	5.57E-04	2.67E-02
hsa-miR-4462	-0.63	4.08E-02	2.32E-01
hsa-miR-4535	-0.62	7.13E-03	1.00E-01
hsa-miR-7704	-0.60	2.55E-02	1.86E-01
hsa-miR-5001-5p	-0.60	2.21E-02	1.84E-01
hsa-miR-1207-5p	-0.57	2.03E-02	1.75E-01
hsa-miR-4271	-0.56	4.97E-03	9.16E-02
hsa-miR-6891-5p	-0.54	4.01E-02	2.32E-01
hsa-miR-4739	-0.54	2.50E-02	1.86E-01
hsa-miR-2861	-0.53	2.35E-02	1.86E-01
hsa-miR-1915-3p	-0.53	2.95E-02	2.00E-01
hsa-miR-1229-5p	-0.52	3.93E-02	2.32E-01
hsa-miR-3960	-0.52	1.69E-03	4.59E-02
hsa-miR-1185-2-3p	-0.52	1.33E-02	1.44E-01
hsa-miR-1185-1-3p	-0.51	1.63E-02	1.57E-01
hsa-miR-3665	-0.51	3.43E-02	2.12E-01
hsa-miR-6124	-0.51	7.16E-03	1.00E-01
hsa-miR-4466	-0.46	1.25E-02	1.38E-01
hsa-miR-1268b	-0.45	1.55E-02	1.53E-01
hsa-miR-4634	-0.43	5.29E-03	9.16E-02
hsa-miR-6090	-0.43	3.16E-02	2.04E-01
hsa-miR-6088	-0.43	2.61E-02	1.86E-01

hsa-miR-8063	-0.42	2.06E-02	1.75E-01
hsa-miR-6756-5p	-0.41	1.20E-02	1.38E-01
hsa-miR-6791-5p	-0.41	1.91E-02	1.75E-01
hsa-miR-939-5p	-0.40	1.97E-02	1.75E-01
hsa-miR-638	-0.39	1.50E-02	1.52E-01
hsa-miR-4745-5p	-0.38	2.95E-02	2.00E-01
hsa-miR-4763-3p	-0.37	4.02E-02	2.32E-01
hsa-miR-6789-5p	-0.36	4.77E-02	2.46E-01
hsa-miR-6728-5p	-0.31	4.99E-02	2.47E-01
hsa-miR-100-5p	expressed in Stable not in Control		
hsa-miR-1208	expressed in Stable not in Control		
hsa-miR-1227-5p	expressed in Stable not in Control		
hsa-miR-1229-3p	expressed in Stable not in Control		
hsa-miR-1273f	expressed in Stable not in Control		
hsa-miR-184	expressed in Stable not in Control		
hsa-miR-187-5p	expressed in Stable not in Control		
hsa-miR-1914-3p	expressed in Stable not in Control		
hsa-miR-194-3p	expressed in Stable not in Control		
hsa-miR-204-5p	expressed in Stable not in Control		
hsa-miR-2276-3p	expressed in Stable not in Control		
hsa-miR-3150b-5p	expressed in Stable not in Control		
hsa-miR-3188	expressed in Stable not in Control		
hsa-miR-339-3p	expressed in Stable not in Control		
hsa-miR-3679-3p	expressed in Stable not in Control		
hsa-miR-4274	expressed in Stable not in Control		
hsa-miR-4433a-3p	expressed in Stable not in Control		
hsa-miR-4716-3p	expressed in Stable not in Control		
hsa-miR-4734	expressed in Stable not in Control		
hsa-miR-4792	expressed in Stable not in Control		
hsa-miR-5010-3p	expressed in Stable not in Control		
hsa-miR-5096	expressed in Stable not in Control		
hsa-miR-6722-3p	expressed in Stable not in Control		
hsa-miR-6731-3p	expressed in Stable not in Control		
hsa-miR-675-3p	expressed in Stable not in Control		
hsa-miR-6785-3p	expressed in Stable not in Control		
hsa-miR-6794-5p	expressed in Stable not in Control		
hsa-miR-6795-3p	expressed in Stable not in Control		
hsa-miR-6806-5p	expressed in Stable not in Control		
hsa-miR-6824-5p	expressed in Stable not in Control		
hsa-miR-6829-5p	expressed in Stable not in Control		
hsa-miR-7-5p	expressed in Stable not in Control		
hsa-miR-7845-5p	expressed in Stable not in Control		
hsa-miR-885-5p	expressed in Stable not in Control		
hsa-miR-887-3p	expressed in Stable not in Control		

List 5. Stable-Acute, upregulated in Stable

	logFC	P-value	FDR
hsa-miR-8069	0.28	4.23E-02	9.88E-01
hsa-miR-3960	0.33	3.04E-02	9.88E-01
hsa-miR-6789-5p	0.36	4.63E-02	9.88E-01
hsa-miR-4530	0.72	1.83E-02	9.88E-01
hsa-miR-194-5p	1.14	3.51E-02	9.88E-01
hsa-miR-215-5p	1.19	4.02E-02	9.88E-01
hsa-miR-1208	expressed in Stable not in Acute		
hsa-miR-1263	expressed in Stable not in Acute		
hsa-miR-184	expressed in Stable not in Acute		
hsa-miR-192-3p	expressed in Stable not in Acute		
hsa-miR-194-3p	expressed in Stable not in Acute		
hsa-miR-204-5p	expressed in Stable not in Acute		
hsa-miR-2276-3p	expressed in Stable not in Acute		
hsa-miR-3150b-5p	expressed in Stable not in Acute		
hsa-miR-339-3p	expressed in Stable not in Acute		
hsa-miR-4433a-3p	expressed in Stable not in Acute		
hsa-miR-4734	expressed in Stable not in Acute		
hsa-miR-4792	expressed in Stable not in Acute		
hsa-miR-500a-5p	expressed in Stable not in Acute		
hsa-miR-5010-3p	expressed in Stable not in Acute		
hsa-miR-5096	expressed in Stable not in Acute		
hsa-miR-675-3p	expressed in Stable not in Acute		
hsa-miR-6829-5p	expressed in Stable not in Acute		
hsa-miR-7-5p	expressed in Stable not in Acute		
hsa-miR-885-5p	expressed in Stable not in Acute		
hsa-miR-887-3p	expressed in Stable not in Acute		

List 6. Stable-Acute, downregulated in Stable

	logFC	P.Value	FDR
hsa-miR-424-5p	-1.42	5.52E-03	5.98E-01
hsa-miR-503-5p	-1.14	3.06E-03	5.68E-01
hsa-miR-221-3p	-1.05	3.93E-03	5.68E-01
hsa-miR-205-5p	-1.02	1.57E-02	9.71E-01
hsa-miR-421	-0.79	2.23E-03	5.68E-01
hsa-miR-222-3p	-0.76	3.95E-02	9.88E-01
hsa-miR-652-3p	-0.63	1.20E-02	8.65E-01
hsa-miR-450a-5p	-0.62	3.04E-02	9.88E-01
hsa-miR-374c-5p	-0.62	2.28E-02	9.88E-01
hsa-miR-374b-5p	-0.60	2.34E-02	9.88E-01
hsa-miR-374a-5p	-0.59	3.72E-02	9.88E-01
hsa-miR-361-5p	-0.45	8.96E-03	7.76E-01
hsa-let-7c-5p	expressed in Acute not in Stable		
hsa-let-7d-5p	expressed in Acute not in Stable		
hsa-miR-10a-5p	expressed in Acute not in Stable		
hsa-miR-10b-5p	expressed in Acute not in Stable		

hsa-miR-143-3p	expressed in Acute not in Stable
hsa-miR-181b-5p	expressed in Acute not in Stable
hsa-miR-196b-5p	expressed in Acute not in Stable
hsa-miR-199a-3p	expressed in Acute not in Stable
hsa-miR-199a-5p	expressed in Acute not in Stable
hsa-miR-199b-5p	expressed in Acute not in Stable
hsa-miR-214-3p	expressed in Acute not in Stable
hsa-miR-221-5p	expressed in Acute not in Stable
hsa-miR-224-5p	expressed in Acute not in Stable
hsa-miR-3151-3p	expressed in Acute not in Stable
hsa-miR-326	expressed in Acute not in Stable
hsa-miR-34a-3p	expressed in Acute not in Stable
hsa-miR-4652-3p	expressed in Acute not in Stable
hsa-miR-4697-5p	expressed in Acute not in Stable
hsa-miR-489-3p	expressed in Acute not in Stable
hsa-miR-522-3p	expressed in Acute not in Stable
hsa-miR-542-5p	expressed in Acute not in Stable
hsa-miR-551b-3p	expressed in Acute not in Stable
hsa-miR-563	expressed in Acute not in Stable
hsa-miR-634	expressed in Acute not in Stable
hsa-miR-6753-3p	expressed in Acute not in Stable
hsa-miR-6757-3p	expressed in Acute not in Stable
hsa-miR-6759-3p	expressed in Acute not in Stable
hsa-miR-6765-3p	expressed in Acute not in Stable
hsa-miR-92b-3p	expressed in Acute not in Stable
hsa-miR-98-3p	expressed in Acute not in Stable

Table S5. Differentially expressed miRNAs during hepatic differentiation comparing iPSCs and DE cells as well as DE cells and HLCs. iPSC, induced pluripotent stem cell; DE, definitive endoderm; HLC, hepatocyte-like cell.

**iPSC-DE**				**DE-HLC**			
**miRNA**	**logFC**	**P.Value**	**adj.P.Val**	**miRNA**	**logFC**	**P.Value**	**adj.P.Val**
hsa-miR-302c-3p	-8.25	3.12E-24	1.19E-21	hsa-miR-371a-5p	-3.64	7.76E-33	2.95E-30
hsa-miR-302a-5p	-8.67	6.27E-24	1.19E-21	hsa-miR-21-5p	4.24	3.29E-32	6.25E-30
hsa-miR-302d-3p	-9.01	1.34E-23	1.69E-21	hsa-miR-302b-3p	-5.50	1.81E-30	2.30E-28
hsa-miR-302a-3p	-10.65	1.42E-19	1.35E-17	hsa-miR-302a-5p	-3.82	9.96E-30	9.47E-28
hsa-miR-371a-5p	-5.68	1.92E-19	1.46E-17	hsa-miR-23b-3p	3.74	1.60E-29	1.22E-27
hsa-miR-302b-3p	-9.16	1.22E-18	7.74E-17	hsa-miR-4516	3.30	7.02E-28	4.45E-26
hsa-miR-21-5p	5.51	1.02E-15	5.53E-14	hsa-miR-27b-3p	3.23	2.64E-27	1.43E-25
hsa-miR-29b-3p	5.25	2.76E-15	1.31E-13	hsa-miR-302d-3p	-3.51	8.65E-27	4.11E-25
hsa-miR-367-3p	-9.41	4.37E-14	1.85E-12	hsa-miR-302c-3p	-3.09	1.23E-26	5.19E-25
hsa-miR-29c-3p	3.68	3.14E-13	1.19E-11	hsa-miR-302a-3p	-5.00	2.35E-26	8.91E-25
hsa-miR-29a-3p	6.46	7.47E-13	2.58E-11	hsa-miR-24-3p	2.98	1.92E-24	6.65E-23
hsa-miR-371a-3p	-8.46	1.23E-12	3.90E-11	hsa-miR-29b-3p	2.86	2.28E-24	7.23E-23
hsa-miR-372-3p	-10.41	3.37E-12	9.84E-11	hsa-miR-29a-3p	3.83	6.97E-23	2.04E-21
hsa-miR-373-3p	-9.61	4.83E-12	1.31E-10	hsa-miR-4281	1.91	1.77E-22	4.81E-21
hsa-let-7f-5p	5.65	1.22E-11	3.10E-10	hsa-miR-4800-5p	-2.74	4.58E-22	1.16E-20
hsa-miR-27b-3p	4.01	1.54E-11	3.66E-10	hsa-miR-22-3p	2.86	1.14E-21	2.71E-20
hsa-miR-200c-3p	-4.58	2.11E-11	4.72E-10	hsa-miR-424-5p	3.17	1.98E-21	4.43E-20
hsa-miR-23b-3p	4.10	2.74E-11	5.78E-10	hsa-miR-367-3p	-4.72	3.15E-21	6.65E-20
hsa-miR-24-3p	3.88	2.42E-10	4.84E-09	hsa-miR-29c-3p	1.93	5.09E-21	1.02E-19
hsa-miR-205-5p	-5.31	3.45E-10	6.56E-09	hsa-miR-373-3p	-5.46	5.49E-21	1.04E-19
hsa-miR-185-5p	2.65	4.40E-10	7.96E-09	hsa-miR-371a-3p	-4.47	2.08E-20	3.76E-19
hsa-miR-34a-5p	3.90	1.71E-09	2.96E-08	hsa-miR-30a-5p	2.21	5.17E-19	8.93E-18
hsa-miR-455-3p	3.22	6.27E-09	1.04E-07	hsa-miR-372-3p	-5.22	7.15E-19	1.18E-17
hsa-miR-6891-5p	-2.52	9.77E-09	1.55E-07	hsa-miR-151a-5p	1.69	1.44E-18	2.28E-17
hsa-miR-2392	-2.88	1.36E-08	2.05E-07	hsa-miR-1268a	-1.96	1.60E-18	2.44E-17
hsa-miR-4634	-2.16	1.40E-08	2.05E-07	hsa-miR-574-3p	1.91	2.65E-18	3.87E-17
hsa-miR-140-3p	2.09	4.02E-08	5.66E-07	hsa-miR-6090	1.61	4.23E-18	5.95E-17

hsa-miR-128-3p	2.30	7.35E-08	9.75E-07	hsa-miR-34a-5p	2.25	1.23E-17	1.67E-16
hsa-miR-4800-5p	-3.22	7.65E-08	9.75E-07	hsa-miR-365a-3p	1.98	1.72E-17	2.25E-16
hsa-let-7a-5p	5.50	7.70E-08	9.75E-07	hsa-miR-575	-1.60	8.15E-17	1.03E-15
hsa-miR-141-3p	-4.50	1.13E-07	1.39E-06	hsa-miR-1246	-2.04	9.03E-17	1.11E-15
hsa-miR-140-5p	2.71	1.41E-07	1.67E-06	hsa-miR-193a-3p	1.75	1.31E-16	1.56E-15
hsa-miR-31-5p	-2.78	1.95E-07	2.25E-06	hsa-miR-5703	-3.24	1.61E-16	1.85E-15
hsa-miR-107	2.80	2.02E-07	2.25E-06	hsa-miR-630	-3.30	2.38E-16	2.66E-15
hsa-miR-575	-2.38	2.25E-07	2.44E-06	hsa-miR-151b	1.27	3.18E-16	3.45E-15
hsa-miR-660-5p	2.57	3.94E-07	4.16E-06	hsa-miR-4497	1.94	4.72E-16	4.99E-15
hsa-miR-6510-5p	-2.61	5.09E-07	5.23E-06	hsa-miR-6780b-5p	-1.46	7.02E-16	7.01E-15
hsa-miR-1229-5p	-2.39	6.13E-07	6.13E-06	hsa-miR-185-5p	1.32	7.03E-16	7.01E-15
hsa-miR-6126	-1.92	9.12E-07	8.89E-06	hsa-miR-23a-3p	2.00	7.19E-16	7.01E-15
hsa-miR-363-3p	-4.12	1.01E-06	9.57E-06	hsa-miR-4532	1.78	1.33E-15	1.26E-14
hsa-miR-103a-3p	2.68	1.08E-06	1.00E-05	hsa-miR-3663-3p	1.79	2.18E-15	2.02E-14
hsa-miR-6090	-2.05	1.29E-06	1.17E-05	hsa-miR-200c-3p	1.91	9.75E-15	8.82E-14
hsa-miR-1246	-2.79	1.33E-06	1.18E-05	hsa-miR-7150	-1.49	1.92E-14	1.69E-13
hsa-miR-4763-3p	-2.08	1.75E-06	1.51E-05	hsa-miR-103a-3p	1.67	2.16E-14	1.87E-13
hsa-miR-30d-5p	1.95	1.81E-06	1.53E-05	hsa-miR-660-5p	1.51	3.62E-14	3.05E-13
hsa-miR-17-5p	2.42	2.02E-06	1.67E-05	hsa-miR-107	1.57	6.32E-14	5.22E-13
hsa-miR-7150	-2.23	3.48E-06	2.82E-05	hsa-miR-1275	-1.32	7.31E-14	5.91E-13
hsa-miR-574-3p	2.25	4.38E-06	3.47E-05	hsa-miR-6510-5p	-1.50	1.27E-13	1.01E-12
hsa-miR-7108-5p	-1.85	4.77E-06	3.70E-05	hsa-miR-8072	-1.21	2.29E-13	1.78E-12
hsa-miR-642b-3p	-2.11	5.10E-06	3.88E-05	hsa-miR-4443	-1.33	3.96E-13	3.01E-12
hsa-miR-148b-3p	1.66	6.37E-06	4.74E-05	hsa-miR-92a-3p	-1.50	6.99E-13	5.21E-12
hsa-miR-874-3p	-1.99	7.35E-06	5.37E-05	hsa-miR-4634	1.00	8.93E-13	6.52E-12
hsa-miR-6780b-5p	-1.91	9.03E-06	6.48E-05	hsa-miR-1915-3p	1.16	1.32E-12	9.48E-12
hsa-miR-1207-5p	-1.82	9.65E-06	6.79E-05	hsa-miR-148b-3p	1.01	1.40E-12	9.77E-12
hsa-miR-20a-5p	2.33	1.40E-05	9.66E-05	hsa-miR-151a-3p	1.14	1.41E-12	9.77E-12
hsa-miR-320a	1.74	2.05E-05	1.39E-04	hsa-miR-221-3p	1.74	3.05E-12	2.07E-11
hsa-miR-7107-5p	-2.81	2.17E-05	1.44E-04	hsa-miR-6850-5p	1.27	3.27E-12	2.18E-11
hsa-miR-30b-5p	2.15	2.31E-05	1.51E-04	hsa-miR-6769b-5p	-1.32	3.39E-12	2.22E-11

hsa-miR-6124	-1.70	2.45E-05	1.58E-04	hsa-miR-4327	1.54	6.33E-12	4.08E-11
hsa-miR-762	-2.27	2.51E-05	1.59E-04	hsa-miR-6089	1.16	9.31E-12	5.90E-11
hsa-miR-134-5p	-1.94	2.72E-05	1.70E-04	hsa-miR-150-3p	1.49	1.14E-11	6.98E-11
hsa-miR-642a-3p	-2.39	3.52E-05	2.15E-04	hsa-miR-186-5p	0.89	1.14E-11	6.98E-11
hsa-miR-4485-5p	1.84	3.56E-05	2.15E-04	hsa-miR-6785-5p	-1.45	2.09E-11	1.26E-10
hsa-miR-6869-5p	-2.06	3.96E-05	2.30E-04	hsa-miR-6800-5p	0.90	2.37E-11	1.41E-10
hsa-miR-6794-5p	-1.73	3.99E-05	2.30E-04	hsa-miR-532-5p	1.50	3.06E-11	1.79E-10
hsa-miR-8072	-1.69	4.00E-05	2.30E-04	hsa-miR-6821-5p	1.15	3.43E-11	1.97E-10
hsa-miR-151a-3p	1.65	4.49E-05	2.55E-04	hsa-miR-505-3p	1.06	5.19E-11	2.94E-10
hsa-miR-6821-5p	-1.85	4.80E-05	2.68E-04	hsa-miR-4716-3p	-1.09	5.27E-11	2.94E-10
hsa-miR-6727-5p	-1.98	5.51E-05	3.01E-04	hsa-miR-4793-5p	-1.35	5.44E-11	3.00E-10
hsa-miR-4462	-2.55	5.55E-05	3.01E-04	hsa-miR-3679-5p	-1.01	6.59E-11	3.58E-10
hsa-miR-4741	-2.05	6.39E-05	3.42E-04	hsa-miR-6891-5p	-0.99	1.08E-10	5.73E-10
hsa-miR-1202	-1.71	6.47E-05	3.42E-04	hsa-miR-7107-5p	-1.61	1.09E-10	5.73E-10
hsa-miR-4270	-2.07	6.98E-05	3.63E-04	hsa-miR-874-3p	1.06	1.34E-10	6.97E-10
hsa-miR-320b	1.67	7.67E-05	3.89E-04	hsa-let-7f-5p	1.72	1.96E-10	1.00E-09
hsa-miR-8063	-1.54	7.74E-05	3.89E-04	hsa-miR-6893-5p	-1.01	2.61E-10	1.31E-09
hsa-miR-3195	-1.72	7.78E-05	3.89E-04	hsa-miR-3653-3p	1.11	2.61E-10	1.31E-09
hsa-miR-106b-5p	1.82	8.04E-05	3.93E-04	hsa-miR-6740-5p	-0.87	2.99E-10	1.48E-09
hsa-miR-4306	1.66	8.06E-05	3.93E-04	hsa-miR-6727-5p	1.15	3.73E-10	1.82E-09
hsa-miR-454-3p	1.79	1.05E-04	5.03E-04	hsa-miR-34b-5p	1.03	4.77E-10	2.29E-09
hsa-miR-1181	-1.91	1.17E-04	5.54E-04	hsa-miR-193b-3p	1.59	5.97E-10	2.83E-09
hsa-miR-34b-5p	1.69	1.18E-04	5.54E-04	hsa-miR-4306	0.95	1.11E-09	5.20E-09
hsa-miR-4793-5p	-2.06	1.23E-04	5.70E-04	hsa-miR-27a-3p	1.41	1.14E-09	5.29E-09
hsa-miR-4651	-1.86	1.31E-04	6.01E-04	hsa-miR-1290	-1.71	1.44E-09	6.56E-09
hsa-miR-186-5p	1.27	1.46E-04	6.61E-04	hsa-miR-5006-5p	-0.94	1.45E-09	6.56E-09
hsa-miR-532-5p	2.20	1.58E-04	7.07E-04	hsa-miR-30e-3p	0.86	1.50E-09	6.69E-09
hsa-miR-126-3p	-2.47	1.62E-04	7.14E-04	hsa-miR-222-3p	1.71	2.54E-09	1.12E-08
hsa-miR-6088	-1.33	1.66E-04	7.17E-04	hsa-miR-20a-5p	-1.14	4.24E-09	1.85E-08
hsa-miR-193b-3p	2.58	1.66E-04	7.17E-04	hsa-miR-130b-3p	-1.12	1.00E-08	4.34E-08
hsa-miR-4284	2.19	1.70E-04	7.24E-04	hsa-miR-93-5p	-0.84	1.12E-08	4.78E-08

hsa-miR-26b-5p	2.23	2.50E-04	1.06E-03	hsa-miR-148a-3p	1.13	2.02E-08	8.54E-08
hsa-miR-5703	-3.25	2.53E-04	1.06E-03	hsa-miR-7110-5p	-1.09	2.65E-08	1.10E-07
hsa-miR-1915-3p	-1.48	2.68E-04	1.11E-03	hsa-miR-188-5p	-0.84	3.38E-08	1.40E-07
hsa-miR-4281	-1.38	2.73E-04	1.11E-03	hsa-miR-3651	-0.96	6.60E-08	2.70E-07
hsa-miR-3162-5p	-1.92	2.74E-04	1.11E-03	hsa-miR-30b-5p	0.95	8.94E-08	3.61E-07
hsa-miR-23a-3p	2.06	2.81E-04	1.12E-03	hsa-miR-6734-5p	-0.83	1.14E-07	4.56E-07
hsa-miR-301a-3p	2.12	2.99E-04	1.19E-03	hsa-miR-99b-5p	1.12	1.27E-07	5.01E-07
hsa-miR-4463	-1.67	3.24E-04	1.27E-03	hsa-miR-374b-5p	1.07	1.32E-07	5.17E-07
hsa-miR-18a-5p	2.04	3.27E-04	1.27E-03	hsa-miR-128-3p	0.75	1.35E-07	5.24E-07
hsa-miR-3940-5p	-1.60	3.68E-04	1.41E-03	hsa-miR-5787	-1.40	1.50E-07	5.75E-07
hsa-miR-93-5p	1.42	3.90E-04	1.48E-03	hsa-miR-30c-5p	0.82	1.58E-07	6.01E-07
hsa-miR-6786-5p	-1.74	4.00E-04	1.51E-03	hsa-miR-642a-3p	-1.04	2.34E-07	8.79E-07
hsa-miR-130b-3p	1.87	4.29E-04	1.60E-03	hsa-miR-6879-5p	-0.66	4.69E-07	1.75E-06
hsa-miR-630	-3.19	4.44E-04	1.64E-03	hsa-miR-17-5p	-0.86	5.51E-07	2.03E-06
hsa-miR-3960	-1.87	5.06E-04	1.85E-03	hsa-miR-30d-5p	0.69	6.12E-07	2.24E-06
hsa-miR-6800-5p	-1.19	5.73E-04	2.07E-03	hsa-miR-125b-5p	1.53	6.52E-07	2.35E-06
hsa-miR-4739	-1.88	6.60E-04	2.36E-03	hsa-miR-26b-5p	1.07	6.56E-07	2.35E-06
hsa-miR-3651	1.66	6.64E-04	2.36E-03	hsa-miR-4530	-1.00	7.24E-07	2.57E-06
hsa-miR-652-5p	-1.45	6.72E-04	2.37E-03	hsa-miR-25-3p	-0.76	7.54E-07	2.64E-06
hsa-miR-4466	-1.20	7.05E-04	2.45E-03	hsa-miR-18a-5p	-0.99	7.58E-07	2.64E-06
hsa-miR-6076	-1.47	7.11E-04	2.45E-03	hsa-miR-2392	-0.80	9.28E-07	3.21E-06
hsa-miR-25-3p	1.47	7.21E-04	2.47E-03	hsa-miR-15a-5p	0.89	9.40E-07	3.22E-06
hsa-miR-7847-3p	-1.41	7.46E-04	2.53E-03	hsa-miR-331-3p	0.99	1.23E-06	4.19E-06
hsa-miR-135a-3p	-1.63	7.64E-04	2.57E-03	hsa-miR-4485-3p	-0.79	1.37E-06	4.62E-06
hsa-miR-505-3p	1.39	7.78E-04	2.59E-03	hsa-miR-455-3p	0.85	1.46E-06	4.85E-06
hsa-miR-27a-3p	2.05	8.18E-04	2.68E-03	hsa-miR-6869-5p	0.83	1.50E-06	4.96E-06
hsa-miR-1225-5p	-1.06	8.18E-04	2.68E-03	hsa-miR-7847-3p	-0.70	1.88E-06	6.16E-06
hsa-miR-6785-5p	-1.83	8.51E-04	2.76E-03	hsa-miR-141-3p	1.31	1.96E-06	6.37E-06
hsa-miR-1268b	-1.13	8.58E-04	2.76E-03	hsa-miR-7108-5p	-0.64	2.17E-06	6.99E-06
hsa-miR-320d	1.75	1.05E-03	3.35E-03	hsa-miR-1207-5p	-0.65	2.58E-06	8.23E-06
hsa-miR-150-3p	-1.81	1.08E-03	3.40E-03	hsa-miR-135a-3p	-0.79	3.13E-06	9.92E-06

hsa-miR-6858-5p	-1.39	1.08E-03	3.40E-03	hsa-miR-4741	0.82	3.35E-06	1.05E-05
hsa-miR-197-3p	1.32	1.14E-03	3.54E-03	hsa-miR-718	0.78	4.47E-06	1.39E-05
hsa-miR-4516	-1.59	1.32E-03	4.07E-03	hsa-miR-484	-0.66	5.72E-06	1.77E-05
hsa-miR-3196	-1.36	1.39E-03	4.27E-03	hsa-miR-106b-5p	-0.72	5.79E-06	1.77E-05
hsa-miR-6728-5p	-1.59	1.42E-03	4.31E-03	hsa-miR-6875-5p	-0.65	5.82E-06	1.77E-05
hsa-miR-5787	-2.33	1.45E-03	4.36E-03	hsa-miR-8063	0.59	7.38E-06	2.22E-05
hsa-miR-1227-5p	-1.48	1.52E-03	4.56E-03	hsa-let-7a-5p	1.45	8.88E-06	2.66E-05
hsa-miR-937-5p	-1.57	1.58E-03	4.68E-03	hsa-miR-1234-3p	-0.63	9.03E-06	2.68E-05
hsa-miR-1185-1-3p	-1.34	1.59E-03	4.68E-03	hsa-miR-210-3p	1.07	1.44E-05	4.25E-05
hsa-miR-6768-5p	-1.43	1.62E-03	4.74E-03	hsa-miR-4499	-0.57	1.57E-05	4.60E-05
hsa-miR-6763-5p	-1.19	1.70E-03	4.94E-03	hsa-miR-1973	-0.72	2.20E-05	6.38E-05
hsa-miR-4459	-2.22	1.81E-03	5.21E-03	hsa-miR-3135b	0.67	2.28E-05	6.57E-05
hsa-miR-6791-5p	-1.22	1.87E-03	5.35E-03	hsa-miR-937-5p	0.73	2.40E-05	6.84E-05
hsa-miR-320e	1.67	2.10E-03	5.97E-03	hsa-miR-1273g-3p	0.96	3.00E-05	8.51E-05
hsa-miR-3141	-1.37	2.42E-03	6.80E-03	hsa-miR-4484	-0.49	3.12E-05	8.78E-05
hsa-miR-4327	-1.68	2.53E-03	7.06E-03	hsa-miR-149-5p	-0.82	3.21E-05	8.98E-05
hsa-miR-7110-5p	-1.58	2.60E-03	7.20E-03	hsa-miR-20b-5p	-0.87	3.78E-05	1.05E-04
hsa-miR-331-3p	1.71	2.69E-03	7.34E-03	hsa-miR-374a-5p	0.97	4.01E-05	1.10E-04
hsa-miR-92a-3p	1.52	2.69E-03	7.34E-03	hsa-miR-513a-5p	-0.63	4.18E-05	1.14E-04
hsa-miR-6749-5p	-1.51	2.70E-03	7.34E-03	hsa-miR-320a	0.55	4.19E-05	1.14E-04
hsa-miR-4257	-1.53	2.72E-03	7.34E-03	hsa-miR-4778-5p	-0.67	4.37E-05	1.18E-04
hsa-miR-6756-5p	-1.22	2.76E-03	7.38E-03	hsa-miR-513b-5p	-0.57	4.94E-05	1.32E-04
hsa-miR-3656	-1.38	2.78E-03	7.38E-03	hsa-miR-4459	-0.99	5.14E-05	1.37E-04
hsa-miR-4499	-1.12	2.88E-03	7.60E-03	hsa-miR-6812-5p	-0.59	5.66E-05	1.48E-04
hsa-miR-6850-5p	-1.33	2.93E-03	7.67E-03	hsa-miR-663a	-1.02	5.67E-05	1.48E-04
hsa-miR-151b	1.02	3.14E-03	8.17E-03	hsa-miR-101-3p	0.85	6.18E-05	1.61E-04
hsa-miR-4443	-1.28	3.43E-03	8.87E-03	hsa-miR-30e-5p	0.80	6.24E-05	1.61E-04
hsa-miR-151a-5p	1.18	3.49E-03	8.97E-03	hsa-miR-324-3p	0.65	6.59E-05	1.69E-04
hsa-miR-423-5p	1.20	3.68E-03	9.38E-03	hsa-miR-4669	-0.57	1.05E-04	2.69E-04
hsa-miR-6824-3p	1.13	3.72E-03	9.41E-03	hsa-miR-1587	-0.71	1.12E-04	2.83E-04
hsa-miR-3679-5p	-1.13	3.92E-03	9.86E-03	hsa-miR-134-5p	-0.59	1.15E-04	2.90E-04

hsa-miR-3663-3p	-1.48	3.97E-03	9.92E-03	hsa-miR-1249-5p	-0.50	1.33E-04	3.34E-04
hsa-miR-183-5p	1.91	4.24E-03	1.05E-02	hsa-miR-4466	0.46	1.46E-04	3.62E-04
hsa-miR-939-5p	-1.14	4.29E-03	1.06E-02	hsa-miR-4728-5p	-0.65	1.65E-04	4.08E-04
hsa-miR-1268a	-1.33	4.35E-03	1.07E-02	hsa-miR-4656	-0.50	2.00E-04	4.91E-04
hsa-miR-429	2.26	4.38E-03	1.07E-02	hsa-miR-6728-5p	-0.62	2.32E-04	5.66E-04
hsa-miR-324-3p	1.32	4.85E-03	1.17E-02	hsa-miR-522-3p	-0.68	3.42E-04	8.27E-04
hsa-miR-210-3p	1.98	4.91E-03	1.18E-02	hsa-miR-320c	-0.57	3.58E-04	8.60E-04
hsa-miR-6880-3p	1.00	5.08E-03	1.21E-02	hsa-miR-3162-5p	-0.62	3.83E-04	9.14E-04
hsa-miR-483-5p	1.94	5.28E-03	1.25E-02	hsa-miR-6775-5p	-0.55	4.27E-04	1.01E-03
hsa-miR-6789-5p	-1.26	5.51E-03	1.30E-02	hsa-miR-4465	0.77	4.41E-04	1.04E-03
hsa-miR-30a-5p	-1.42	5.53E-03	1.30E-02	hsa-miR-4298	-0.52	5.44E-04	1.28E-03
hsa-miR-365a-3p	1.36	6.21E-03	1.44E-02	hsa-miR-17-3p	-0.58	5.58E-04	1.30E-03
hsa-miR-4716-3p	-1.14	6.23E-03	1.44E-02	hsa-miR-4721	-0.53	6.33E-04	1.47E-03
hsa-miR-6851-3p	1.00	6.34E-03	1.46E-02	hsa-miR-3665	0.45	8.09E-04	1.86E-03
hsa-miR-130a-3p	-1.32	6.67E-03	1.53E-02	hsa-miR-6867-5p	-0.53	8.68E-04	1.99E-03
hsa-miR-6763-3p	1.07	6.78E-03	1.54E-02	hsa-miR-5585-3p	-0.54	9.18E-04	2.09E-03
hsa-miR-328-5p	-1.28	6.86E-03	1.55E-02	hsa-miR-197-3p	0.44	1.00E-03	2.26E-03
hsa-miR-6893-5p	-1.09	7.56E-03	1.70E-02	hsa-miR-4713-3p	-0.49	1.01E-03	2.28E-03
hsa-miR-4687-3p	-1.09	7.65E-03	1.71E-02	hsa-miR-6127	-0.40	1.09E-03	2.43E-03
hsa-miR-6125	-1.05	7.72E-03	1.72E-02	hsa-miR-135b-5p	-0.67	1.13E-03	2.51E-03
hsa-miR-6808-5p	-1.49	7.86E-03	1.74E-02	hsa-miR-6768-5p	0.49	1.14E-03	2.52E-03
hsa-miR-6785-3p	0.96	7.94E-03	1.74E-02	hsa-miR-652-5p	-0.46	1.18E-03	2.59E-03
hsa-miR-664b-3p	0.88	8.31E-03	1.82E-02	hsa-miR-6132	-0.54	1.19E-03	2.59E-03
hsa-miR-135b-5p	1.61	8.39E-03	1.82E-02	hsa-miR-6724-5p	-0.47	1.20E-03	2.59E-03
hsa-miR-221-3p	1.60	8.43E-03	1.82E-02	hsa-miR-140-3p	-0.37	1.30E-03	2.80E-03
hsa-miR-6865-3p	1.06	8.98E-03	1.93E-02	hsa-miR-15b-5p	-0.57	1.32E-03	2.82E-03
hsa-miR-18b-5p	1.52	9.07E-03	1.94E-02	hsa-miR-4271	0.48	1.44E-03	3.07E-03
hsa-miR-6867-5p	-1.23	9.31E-03	1.98E-02	hsa-miR-638	-0.39	1.85E-03	3.92E-03
hsa-miR-6777-3p	0.87	9.66E-03	2.04E-02	hsa-miR-6088	0.35	2.08E-03	4.40E-03
hsa-miR-6068	-1.04	9.80E-03	2.06E-02	hsa-miR-378a-3p	-0.50	2.14E-03	4.48E-03
hsa-miR-4758-3p	1.02	9.84E-03	2.06E-02	hsa-miR-4463	0.47	2.18E-03	4.55E-03

hsa-miR-222-3p	1.91	1.05E-02	2.18E-02	hsa-miR-183-5p	0.68	2.35E-03	4.88E-03
hsa-miR-1224-5p	-1.05	1.06E-02	2.19E-02	hsa-miR-5739	-0.40	2.81E-03	5.80E-03
hsa-miR-4530	-1.42	1.08E-02	2.21E-02	hsa-miR-7704	-0.43	2.99E-03	6.15E-03
hsa-miR-101-3p	1.54	1.11E-02	2.27E-02	hsa-miR-4739	-0.54	3.14E-03	6.43E-03
hsa-miR-4271	-1.12	1.13E-02	2.29E-02	hsa-miR-1273f	-0.38	4.29E-03	8.73E-03
hsa-miR-425-5p	1.10	1.13E-02	2.29E-02	hsa-miR-4430	-0.47	4.61E-03	9.32E-03
hsa-miR-125a-5p	-1.30	1.17E-02	2.35E-02	hsa-miR-3960	0.50	4.65E-03	9.35E-03
hsa-miR-766-3p	0.97	1.21E-02	2.42E-02	hsa-miR-19b-3p	-0.66	4.85E-03	9.69E-03
hsa-miR-33b-3p	1.03	1.23E-02	2.44E-02	hsa-miR-4428	-0.55	5.21E-03	1.04E-02
hsa-miR-6861-3p	0.79	1.33E-02	2.63E-02	hsa-miR-1825	-0.40	5.38E-03	1.06E-02
hsa-miR-4778-5p	-1.16	1.36E-02	2.67E-02	hsa-miR-4449	-0.53	5.46E-03	1.07E-02
hsa-miR-4485-3p	1.11	1.41E-02	2.75E-02	hsa-miR-619-5p	-0.47	5.55E-03	1.09E-02
hsa-miR-6812-3p	0.91	1.43E-02	2.78E-02	hsa-miR-125a-5p	0.48	5.92E-03	1.15E-02
hsa-miR-1237-3p	0.94	1.44E-02	2.80E-02	hsa-miR-18b-5p	-0.53	6.37E-03	1.23E-02
hsa-miR-3135b	-1.09	1.50E-02	2.89E-02	hsa-miR-629-3p	-0.49	7.08E-03	1.37E-02
hsa-miR-6731-3p	0.86	1.52E-02	2.91E-02	hsa-miR-2861	-0.34	7.37E-03	1.41E-02
hsa-miR-1290	-1.77	1.53E-02	2.92E-02	hsa-miR-6749-5p	0.45	7.46E-03	1.42E-02
hsa-miR-3665	-0.95	1.56E-02	2.93E-02	hsa-miR-3151-3p	-0.24	7.80E-03	1.48E-02
hsa-miR-425-3p	1.08	1.56E-02	2.93E-02	hsa-miR-320b	0.36	8.33E-03	1.57E-02
hsa-miR-6734-5p	-1.02	1.57E-02	2.93E-02	hsa-miR-378i	-0.42	8.73E-03	1.64E-02
hsa-miR-361-5p	0.95	1.57E-02	2.93E-02	hsa-miR-1229-5p	-0.38	9.62E-03	1.80E-02
hsa-miR-1249-5p	-0.91	1.58E-02	2.94E-02	hsa-miR-939-5p	0.34	1.03E-02	1.92E-02
hsa-miR-6800-3p	1.05	1.63E-02	3.02E-02	hsa-miR-8485	-0.44	1.06E-02	1.97E-02
hsa-miR-30c-5p	1.01	1.69E-02	3.12E-02	hsa-miR-4462	-0.51	1.10E-02	2.04E-02
hsa-miR-3652	-0.98	1.71E-02	3.14E-02	hsa-miR-3198	-0.37	1.22E-02	2.23E-02
hsa-miR-7974	0.92	1.73E-02	3.16E-02	hsa-miR-5100	0.69	1.39E-02	2.53E-02
hsa-miR-4745-5p	-1.08	1.84E-02	3.34E-02	hsa-miR-6803-5p	-0.57	1.45E-02	2.63E-02
hsa-miR-26a-5p	1.06	1.86E-02	3.37E-02	hsa-miR-1249-3p	0.32	1.47E-02	2.67E-02
hsa-miR-5006-5p	-0.93	1.94E-02	3.48E-02	hsa-miR-6717-5p	-0.38	1.58E-02	2.85E-02
hsa-miR-15a-5p	1.16	1.94E-02	3.48E-02	hsa-miR-1185-1-3p	0.34	1.59E-02	2.85E-02
hsa-miR-4725-5p	1.12	1.95E-02	3.49E-02	hsa-miR-371b-5p	-0.43	1.60E-02	2.86E-02

hsa-let-7b-3p	1.05	1.99E-02	3.54E-02
hsa-miR-188-5p	-0.94	2.03E-02	3.56E-02
hsa-miR-4669	-0.98	2.03E-02	3.56E-02
hsa-miR-6858-3p	0.81	2.04E-02	3.56E-02
hsa-miR-378i	1.11	2.12E-02	3.70E-02
hsa-let-7f-1-3p	0.92	2.13E-02	3.70E-02
hsa-miR-6740-5p	-0.81	2.16E-02	3.72E-02
hsa-miR-4649-3p	1.00	2.17E-02	3.72E-02
hsa-miR-6508-5p	1.15	2.21E-02	3.79E-02
hsa-miR-22-3p	1.30	2.24E-02	3.82E-02
hsa-miR-378a-3p	1.09	2.33E-02	3.96E-02
hsa-miR-4664-3p	1.00	2.40E-02	4.05E-02
hsa-miR-30e-3p	0.82	2.45E-02	4.11E-02
hsa-miR-6737-3p	0.93	2.45E-02	4.11E-02
hsa-miR-6760-3p	0.73	2.52E-02	4.20E-02
hsa-miR-4442	-0.71	2.60E-02	4.31E-02
hsa-miR-550a-5p	0.96	2.62E-02	4.31E-02
hsa-miR-6813-3p	0.87	2.63E-02	4.31E-02
hsa-miR-4323	0.68	2.65E-02	4.31E-02
hsa-miR-638	-0.82	2.65E-02	4.31E-02
hsa-miR-6087	-0.88	2.66E-02	4.31E-02
hsa-miR-125a-3p	-0.97	2.66E-02	4.31E-02
hsa-miR-1273e	-0.90	2.91E-02	4.67E-02
hsa-miR-2116-3p	0.99	2.91E-02	4.67E-02
hsa-miR-148a-3p	1.15	2.96E-02	4.73E-02
hsa-miR-1260b	-1.15	3.11E-02	4.95E-02
hsa-miR-1908-3p	0.75	3.14E-02	4.96E-02
hsa-miR-1304-3p	1.09	3.16E-02	4.98E-02

hsa-miR-129-2-3p	-0.36	1.73E-02	3.07E-02
hsa-miR-125a-3p	-0.34	2.00E-02	3.53E-02
hsa-miR-6076	0.32	2.18E-02	3.83E-02
hsa-miR-4299	-0.41	2.29E-02	4.02E-02
hsa-miR-19a-3p	-0.56	2.39E-02	4.16E-02
hsa-miR-642b-3p	-0.32	2.50E-02	4.34E-02
hsa-miR-6068	-0.29	2.76E-02	4.77E-02

Table S6. MicroRNAs unique to differentiation stages iPSC (induced pluripotent stem cells), DE (definitive endoderm cells) and HLC (hepatocyte-like cells).

**Unique to iPSC**<br>**n = 69**	**Unique to DE**<br>**n = 18**	**Unique to HLC**<br>**n = 56**
hsa-miR-106a-3p	hsa-miR-224-5p	hsa-let-7e-5p
hsa-miR-1208	hsa-miR-3173-3p	hsa-miR-100-5p
hsa-miR-1226-5p	hsa-miR-372-5p	hsa-miR-10b-5p
hsa-miR-1233-5p	hsa-miR-373-5p	hsa-miR-122-3p
hsa-miR-1236-5p	hsa-miR-3926	hsa-miR-122-5p
hsa-miR-124-3p	hsa-miR-4652-3p	hsa-miR-1270
hsa-miR-1469	hsa-miR-4697-5p	hsa-miR-1271-5p
hsa-miR-19b-1-5p	hsa-miR-497-5p	hsa-miR-138-5p
hsa-miR-3132	hsa-miR-5010-3p	hsa-miR-145-5p
hsa-miR-3150b-5p	hsa-miR-516a-5p	hsa-miR-146a-5p
hsa-miR-3158-5p	hsa-miR-6075	hsa-miR-152-3p
hsa-miR-3610	hsa-miR-629-5p	hsa-miR-181b-5p
hsa-miR-3622a-5p	hsa-miR-6732-5p	hsa-miR-187-5p
hsa-miR-3682-3p	hsa-miR-6778-5p	hsa-miR-192-3p
hsa-miR-3911	hsa-miR-6809-5p	hsa-miR-193a-5p
hsa-miR-3938	hsa-miR-7-2-3p	hsa-miR-193b-5p
hsa-miR-4419a	hsa-miR-9-3p	hsa-miR-194-5p
hsa-miR-4646-5p	hsa-miR-9-5p	hsa-miR-199a-3p
hsa-miR-4695-5p		hsa-miR-199a-5p
hsa-miR-4701-3p		hsa-miR-21-3p
hsa-miR-4728-3p		hsa-miR-215-5p
hsa-miR-4734		hsa-miR-218-5p

hsa-miR-4738-3p
hsa-miR-4758-5p
hsa-miR-4767
hsa-miR-4792
hsa-miR-492
hsa-miR-498
hsa-miR-512-5p
hsa-miR-515-3p
hsa-miR-515-5p
hsa-miR-516b-5p
hsa-miR-518a-3p
hsa-miR-518b
hsa-miR-518c-5p
hsa-miR-518e-3p
hsa-miR-519b-3p
hsa-miR-519c-3p
hsa-miR-519d-3p
hsa-miR-520a-5p
hsa-miR-520b
hsa-miR-520d-3p
hsa-miR-520f-3p
hsa-miR-520g-3p
hsa-miR-520h
hsa-miR-525-5p
hsa-miR-526b-5p

hsa-miR-22-5p
hsa-miR-221-5p
hsa-miR-28-3p
hsa-miR-29b-1-5p
hsa-miR-30a-3p
hsa-miR-3188
hsa-miR-326
hsa-miR-345-5p
hsa-miR-361-3p
hsa-miR-362-3p
hsa-miR-362-5p
hsa-miR-3659
hsa-miR-4274
hsa-miR-4291
hsa-miR-450a-5p
hsa-miR-455-5p
hsa-miR-4666b
hsa-miR-483-3p
hsa-miR-500a-3p
hsa-miR-500a-5p
hsa-miR-501-3p
hsa-miR-501-5p
hsa-miR-502-3p
hsa-miR-503-5p
hsa-miR-505-5p

hsa-miR-563
hsa-miR-598-3p
hsa-miR-6073
hsa-miR-636
hsa-miR-663b
hsa-miR-6738-5p
hsa-miR-6765-3p
hsa-miR-6767-5p
hsa-miR-6779-5p
hsa-miR-6784-5p
hsa-miR-6797-5p
hsa-miR-6801-3p
hsa-miR-6839-5p
hsa-miR-6845-5p
hsa-miR-6887-5p
hsa-miR-7152-3p
hsa-miR-760
hsa-miR-769-5p
hsa-miR-8064
hsa-miR-8071
hsa-miR-8087
hsa-miR-936

hsa-miR-532-3p
hsa-miR-5571-5p
hsa-miR-664a-3p
hsa-miR-6722-3p
hsa-miR-6759-3p
hsa-miR-7114-5p
hsa-miR-744-5p
hsa-miR-7845-5p
hsa-miR-99a-5p

Table S7. Differentially expressed miRNAs between control and CAD samples.

	Control - CAD		
	**logFC**	**P-value**	**FDR**
hsa-miR-6869-5p	-1.31	4.96E-07	2.15E-04
hsa-miR-574-3p	0.929	2.71E-05	5.86E-03
hsa-miR-4463	-0.64	1.84E-04	2.44E-02
hsa-miR-6850-5p	-0.75	2.54E-04	2.44E-02
hsa-miR-92a-3p	0.617	3.38E-04	2.44E-02
hsa-miR-93-5p	0.49	4.11E-04	2.44E-02
hsa-miR-4497	-0.85	4.27E-04	2.44E-02
hsa-miR-6727-5p	-0.77	4.51E-04	2.44E-02
hsa-miR-6800-5p	-0.69	8.87E-04	3.88E-02
hsa-miR-1271-5p	0.615	8.96E-04	3.88E-02
hsa-miR-4689	-0.63	1.01E-03	3.98E-02
hsa-miR-6821-5p	-0.96	1.41E-03	4.51E-02
hsa-miR-149-5p	0.705	1.45E-03	4.51E-02
hsa-miR-8485	0.728	1.51E-03	4.51E-02
hsa-miR-150-3p	-1.07	1.63E-03	4.51E-02
hsa-miR-4741	-0.7	1.67E-03	4.51E-02
hsa-miR-4271	-0.57	2.13E-03	5.42E-02
hsa-miR-933	0.441	2.33E-03	5.61E-02
hsa-miR-4327	-1.05	2.83E-03	6.45E-02
hsa-miR-3195	-0.62	3.15E-03	6.46E-02
hsa-miR-937-5p	-0.77	3.25E-03	6.46E-02
hsa-miR-129-2-3p	0.474	3.28E-03	6.46E-02
hsa-miR-6756-5p	-0.43	5.62E-03	1.06E-01

hsa-miR-130a-3p	0.739	6.70E-03	1.14E-01
hsa-miR-4634	-0.39	6.73E-03	1.14E-01
hsa-miR-320a	0.407	6.87E-03	1.14E-01
hsa-miR-4535	-0.57	7.75E-03	1.23E-01
hsa-miR-638	-0.39	7.93E-03	1.23E-01
hsa-miR-762	-0.73	1.01E-02	1.46E-01
hsa-miR-19a-3p	0.751	1.01E-02	1.46E-01
hsa-miR-5001-5p	-0.62	1.06E-02	1.48E-01
hsa-miR-3960	-0.42	1.17E-02	1.59E-01
hsa-miR-130b-3p	0.819	1.26E-02	1.62E-01
hsa-miR-939-5p	-0.4	1.27E-02	1.62E-01
hsa-miR-8063	-0.41	1.33E-02	1.65E-01
hsa-miR-6124	-0.43	1.45E-02	1.74E-01
hsa-miR-3180-5p	0.271	1.53E-02	1.78E-01
hsa-miR-193a-3p	0.593	1.56E-02	1.78E-01
hsa-miR-4739	-0.53	1.70E-02	1.85E-01
hsa-miR-193a-5p	0.515	1.71E-02	1.85E-01
hsa-miR-4466	-0.41	1.87E-02	1.88E-01
hsa-miR-874-3p	-0.57	1.89E-02	1.88E-01
hsa-miR-1185-2-3p	-0.46	1.91E-02	1.88E-01
hsa-miR-6870-3p	0.299	1.93E-02	1.88E-01
hsa-miR-3663-3p	-0.83	1.95E-02	1.88E-01
hsa-miR-367-3p	0.553	2.11E-02	1.93E-01
hsa-miR-2861	-0.5	2.15E-02	1.93E-01
hsa-miR-6858-3p	0.323	2.16E-02	1.93E-01

hsa-miR-17-5p	0.649	2.22E-02	1.93E-01
hsa-miR-18a-5p	0.771	2.22E-02	1.93E-01
hsa-miR-6090	-0.42	2.40E-02	2.02E-01
hsa-miR-210-3p	0.626	2.42E-02	2.02E-01
hsa-miR-20a-5p	0.733	2.53E-02	2.06E-01
hsa-miR-7641	0.647	2.75E-02	2.17E-01
hsa-miR-744-5p	0.462	2.77E-02	2.17E-01
hsa-miR-6798-5p	0.706	2.83E-02	2.17E-01
hsa-miR-6791-5p	-0.36	2.90E-02	2.17E-01
hsa-miR-3651	0.426	2.93E-02	2.17E-01
hsa-miR-4462	-0.62	2.96E-02	2.17E-01
hsa-miR-6737-3p	0.324	3.06E-02	2.21E-01
hsa-miR-1185-1-3p	-0.43	3.14E-02	2.21E-01
hsa-miR-1915-3p	-0.48	3.19E-02	2.21E-01
hsa-miR-766-3p	0.371	3.24E-02	2.21E-01
hsa-miR-484	0.414	3.26E-02	2.21E-01
hsa-miR-1260a	0.734	3.43E-02	2.27E-01
hsa-miR-33b-3p	0.378	3.45E-02	2.27E-01
hsa-miR-1825	0.298	3.58E-02	2.28E-01
hsa-miR-1207-5p	-0.48	3.58E-02	2.28E-01
hsa-miR-4532	-0.71	3.70E-02	2.29E-01
hsa-miR-1268b	-0.37	3.71E-02	2.29E-01
hsa-miR-30a-3p	0.716	3.81E-02	2.32E-01
hsa-miR-6728-5p	-0.3	4.11E-02	2.47E-01
hsa-miR-7704	-0.51	4.38E-02	2.59E-01

hsa-miR-1225-5p	-0.36	4.43E-02	2.59E-01
hsa-miR-125a-5p	0.393	4.60E-02	2.64E-01
hsa-miR-4428	0.519	4.69E-02	2.64E-01
hsa-miR-4666b	0.271	4.69E-02	2.64E-01
hsa-miR-6749-5p	-0.76	4.80E-02	2.65E-01
hsa-miR-126-3p	0.861	4.84E-02	2.65E-01
hsa-miR-27a-3p	0.566	4.99E-02	2.70E-01

Table S8. Differentially expressed miRNAs, pairwise comparison between patient groups.

Control-Acute CAD				Control-Stable CAD				Stable-Acute CAD			
	logFC	P-value	FDR		logFC	P-value	FDR		logFC	P-value	FDR
hsa-miR-6869-5p	-1.24	2.25E-05	9.73E-03	hsa-miR-6869-5p	-1.34	1.40E-06	6.07E-04	hsa-miR-503-5p	-1.14	3.06E-03	5.68E-01
hsa-miR-574-3p	0.88	5.94E-04	1.29E-01	hsa-miR-574-3p	0.95	6.27E-05	1.36E-02	hsa-miR-221-3p	-1.05	3.93E-03	5.68E-01
hsa-miR-4463	-0.67	1.33E-03	1.92E-01	hsa-miR-4497	-0.90	5.35E-04	2.67E-02	hsa-miR-421	-0.79	2.23E-03	5.68E-01
hsa-miR-92a-3p	0.59	4.11E-03	3.17E-01	hsa-miR-6727-5p	-0.86	1.99E-04	2.67E-02	hsa-miR-424-5p	-1.42	5.52E-03	5.98E-01
hsa-miR-6850-5p	-0.65	5.30E-03	3.17E-01	hsa-miR-6850-5p	-0.79	3.17E-04	2.67E-02	hsa-miR-361-5p	-0.45	8.96E-03	7.76E-01
hsa-miR-933	0.49	6.13E-03	3.17E-01	hsa-miR-3195	-0.74	6.78E-04	2.67E-02	hsa-miR-652-3p	-0.63	1.20E-02	8.65E-01
hsa-miR-93-5p	0.44	6.39E-03	3.17E-01	hsa-miR-4689	-0.70	6.30E-04	2.67E-02	hsa-miR-205-5p	-1.02	1.57E-02	9.71E-01
hsa-miR-210-3p	0.92	7.14E-03	3.17E-01	hsa-miR-4463	-0.63	5.57E-04	2.67E-02	hsa-miR-222-3p	-0.76	3.95E-02	9.88E-01
hsa-miR-4497	-0.75	7.53E-03	3.17E-01	hsa-miR-93-5p	0.51	6.51E-04	2.67E-02	hsa-miR-450a-5p	-0.62	3.04E-02	9.88E-01
hsa-miR-150-3p	-1.10	8.35E-03	3.17E-01	hsa-miR-92a-3p	0.63	6.72E-04	2.67E-02	hsa-miR-374c-5p	-0.62	2.28E-02	9.88E-01
hsa-miR-4271	-0.60	8.76E-03	3.17E-01	hsa-miR-149-5p	0.80	5.94E-04	2.67E-02	hsa-miR-374b-5p	-0.60	2.34E-02	9.88E-01
hsa-miR-424-5p	-1.55	8.80E-03	3.17E-01	hsa-miR-4741	-0.77	1.23E-03	3.80E-02	hsa-miR-374a-5p	-0.59	3.72E-02	9.88E-01
hsa-miR-4327	-1.08	1.19E-02	3.47E-01	hsa-miR-6800-5p	-0.73	1.09E-03	3.80E-02	hsa-miR-8069	0.28	4.23E-02	9.88E-01
hsa-miR-1271-5p	0.54	1.22E-02	3.47E-01	hsa-miR-1271-5p	0.65	1.18E-03	3.80E-02	hsa-miR-3960	0.33	3.04E-02	9.88E-01
hsa-miR-8485	0.69	1.22E-02	3.47E-01	hsa-miR-6821-5p	-1.02	1.53E-03	4.41E-02	hsa-miR-6789-5p	0.36	4.63E-02	9.88E-01
hsa-miR-6800-5p	-0.60	1.28E-02	3.47E-01	hsa-miR-3960	-0.52	1.69E-03	4.59E-02	hsa-miR-4530	0.72	1.83E-02	9.88E-01
hsa-miR-6727-5p	-0.58	1.42E-02	3.62E-01	hsa-miR-8485	0.74	2.61E-03	6.66E-02	hsa-miR-194-5p	1.14	3.51E-02	9.88E-01
hsa-miR-3663-3p	-1.06	1.56E-02	3.63E-01	hsa-miR-937-5p	-0.83	3.07E-03	7.39E-02	hsa-miR-215-5p	1.19	4.02E-02	9.88E-01
hsa-miR-503-5p	-1.03	1.59E-02	3.63E-01	hsa-miR-19a-3p	0.89	3.33E-03	7.59E-02				
hsa-miR-6756-5p	-0.45	1.82E-02	3.80E-01	hsa-miR-150-3p	-1.06	3.56E-03	7.72E-02				
hsa-miR-6821-5p	-0.81	1.92E-02	3.80E-01	hsa-miR-4271	-0.56	4.97E-03	9.16E-02				
hsa-miR-6858-3p	0.41	1.93E-02	3.80E-01	hsa-miR-4634	-0.43	5.29E-03	9.16E-02				
hsa-miR-4530	0.81	2.26E-02	4.10E-01	hsa-miR-361-5p	0.49	5.27E-03	9.16E-02				
hsa-miR-4689	-0.48	2.27E-02	4.10E-01	hsa-miR-129-2-3p	0.50	4.54E-03	9.16E-02				
hsa-miR-5001-5p	-0.69	2.41E-02	4.17E-01	hsa-miR-130a-3p	0.82	4.92E-03	9.16E-02				
hsa-miR-129-2-3p	0.43	2.71E-02	4.19E-01	hsa-miR-18a-5p	0.96	5.54E-03	9.23E-02				
hsa-miR-766-3p	0.48	2.74E-02	4.19E-01	hsa-miR-17-5p	0.80	5.76E-03	9.23E-02				
hsa-miR-638	-0.40	2.77E-02	4.19E-01	hsa-miR-4327	-1.03	5.99E-03	9.26E-02				

hsa-miR-6068	-0.43	2.80E-02	4.19E-01
hsa-miR-4741	-0.55	2.92E-02	4.21E-01
hsa-miR-8069	0.34	3.59E-02	4.83E-01
hsa-miR-149-5p	0.50	3.62E-02	4.83E-01
hsa-miR-33b-3p	0.47	3.72E-02	4.83E-01
hsa-miR-937-5p	-0.63	3.79E-02	4.83E-01
hsa-miR-193a-3p	0.60	4.94E-02	5.12E-01

hsa-miR-933	0.42	6.42E-03	9.58E-02
hsa-miR-4535	-0.62	7.13E-03	1.00E-01
hsa-miR-6124	-0.51	7.16E-03	1.00E-01
hsa-miR-874-3p	-0.68	7.73E-03	1.03E-01
hsa-miR-320a	0.43	7.86E-03	1.03E-01
hsa-miR-744-5p	0.56	1.01E-02	1.28E-01
hsa-miR-762	-0.79	1.05E-02	1.30E-01
hsa-miR-4466	-0.46	1.25E-02	1.38E-01
hsa-miR-6756-5p	-0.41	1.20E-02	1.38E-01
hsa-miR-652-3p	0.62	1.23E-02	1.38E-01
hsa-miR-130b-3p	0.89	1.22E-02	1.38E-01
hsa-miR-1185-2-3p	-0.52	1.33E-02	1.44E-01
hsa-miR-20a-5p	0.86	1.36E-02	1.44E-01
hsa-miR-3180-5p	0.30	1.46E-02	1.51E-01
hsa-miR-638	-0.39	1.50E-02	1.52E-01
hsa-miR-1268b	-0.45	1.55E-02	1.53E-01
hsa-miR-1185-1-3p	-0.51	1.63E-02	1.57E-01
hsa-miR-193a-5p	0.55	1.83E-02	1.72E-01
hsa-miR-1207-5p	-0.57	2.03E-02	1.75E-01
hsa-miR-8063	-0.42	2.06E-02	1.75E-01
hsa-miR-6791-5p	-0.41	1.91E-02	1.75E-01
hsa-miR-939-5p	-0.40	1.97E-02	1.75E-01
hsa-miR-221-3p	0.81	2.01E-02	1.75E-01
hsa-miR-5001-5p	-0.60	2.21E-02	1.84E-01
hsa-miR-4532	-0.81	2.64E-02	1.86E-01
hsa-miR-7704	-0.60	2.55E-02	1.86E-01
hsa-miR-4739	-0.54	2.50E-02	1.86E-01
hsa-miR-2861	-0.53	2.35E-02	1.86E-01
hsa-miR-6088	-0.43	2.61E-02	1.86E-01
hsa-miR-6870-3p	0.31	2.66E-02	1.86E-01
hsa-miR-125a-5p	0.47	2.39E-02	1.86E-01
hsa-miR-193a-3p	0.59	2.55E-02	1.86E-01
hsa-miR-27a-3p	0.68	2.63E-02	1.86E-01
hsa-miR-30a-3p	0.82	2.55E-02	1.86E-01
hsa-miR-1915-3p	-0.53	2.95E-02	2.00E-01
hsa-miR-4745-5p	-0.38	2.95E-02	2.00E-01

hsa-miR-7641	0.69	3.01E-02	2.00E-01
hsa-miR-1260a	0.81	3.05E-02	2.00E-01
hsa-miR-6090	-0.43	3.16E-02	2.04E-01
hsa-miR-367-3p	0.56	3.21E-02	2.05E-01
hsa-miR-145-5p	1.91	3.37E-02	2.11E-01
hsa-miR-3665	-0.51	3.43E-02	2.12E-01
hsa-miR-6798-5p	0.72	3.74E-02	2.28E-01
hsa-miR-4462	-0.63	4.08E-02	2.32E-01
hsa-miR-6891-5p	-0.54	4.01E-02	2.32E-01
hsa-miR-1229-5p	-0.52	3.93E-02	2.32E-01
hsa-miR-4763-3p	-0.37	4.02E-02	2.32E-01
hsa-miR-484	0.43	4.04E-02	2.32E-01
hsa-miR-4484	0.28	4.54E-02	2.42E-01
hsa-miR-23a-3p	0.53	4.58E-02	2.42E-01
hsa-miR-4428	0.57	4.33E-02	2.42E-01
hsa-miR-494-3p	0.64	4.49E-02	2.42E-01
hsa-miR-19b-3p	0.67	4.42E-02	2.42E-01
hsa-miR-126-3p	0.95	4.44E-02	2.42E-01
hsa-miR-106b-5p	0.46	4.70E-02	2.45E-01
hsa-miR-6789-5p	-0.36	4.77E-02	2.46E-01
hsa-miR-3663-3p	-0.72	4.90E-02	2.47E-01
hsa-miR-6728-5p	-0.31	4.99E-02	2.47E-01
hsa-miR-205-5p	0.80	4.97E-02	2.47E-01

Table S9. MicroRNAs expressed in one or two groups based on the microarray analysis. A miRNA was considered expressed if its signal was above threshold in at least half of the samples.

Expressed in control but not in acute CAD n = 7	Expressed in acute CAD but not in control n= 36	Expressed in control but not in stable CAD n = 16	Expressed in stable CAD but not in control n = 35	Expressed in stable CAD but not in acute CAD n = 20	Expressed in acute CAD but not stable CAD n = 30
hsa-miR-1263	hsa-let-7c-5p	hsa-miR-10a-5p	hsa-miR-100-5p	hsa-miR-1208	hsa-let-7c-5p
hsa-miR-192-3p	hsa-let-7d-5p	hsa-miR-10b-5p	hsa-miR-1208	hsa-miR-1263	hsa-let-7d-5p
hsa-miR-486-5p	hsa-miR-100-5p	hsa-miR-196b-5p	hsa-miR-1227-5p	hsa-miR-184	hsa-miR-10a-5p
hsa-miR-500a-5p	hsa-miR-1227-5p	hsa-miR-199a-3p	hsa-miR-1229-3p	hsa-miR-192-3p	hsa-miR-10b-5p
hsa-miR-517a-3p	hsa-miR-1229-3p	hsa-miR-199a-5p	hsa-miR-1273f	hsa-miR-194-3p	hsa-miR-143-3p
hsa-miR-517c-3p	hsa-miR-1273f	hsa-miR-3151-3p	hsa-miR-184	hsa-miR-204-5p	hsa-miR-181b-5p
hsa-miR-629-5p	hsa-miR-143-3p	hsa-miR-326	hsa-miR-187-5p	hsa-miR-2276-3p	hsa-miR-196b-5p
	hsa-miR-181b-5p	hsa-miR-486-5p	hsa-miR-1914-3p	hsa-miR-3150b-5p	hsa-miR-199a-3p
	hsa-miR-187-5p	hsa-miR-489-3p	hsa-miR-194-3p	hsa-miR-339-3p	hsa-miR-199a-5p
	hsa-miR-1914-3p	hsa-miR-517a-3p	hsa-miR-204-5p	hsa-miR-4433a-3p	hsa-miR-199b-5p
	hsa-miR-199b-5p	hsa-miR-517c-3p	hsa-miR-2276-3p	hsa-miR-4734	hsa-miR-214-3p
	hsa-miR-214-3p	hsa-miR-522-3p	hsa-miR-3150b-5p	hsa-miR-4792	hsa-miR-221-5p
	hsa-miR-221-5p	hsa-miR-563	hsa-miR-3188	hsa-miR-500a-5p	hsa-miR-224-5p
	hsa-miR-224-5p	hsa-miR-629-5p	hsa-miR-339-3p	hsa-miR-5010-3p	hsa-miR-3151-3p
	hsa-miR-3188	hsa-miR-634	hsa-miR-3679-3p	hsa-miR-5096	hsa-miR-326
	hsa-miR-34a-3p	hsa-miR-6759-3p	hsa-miR-4274	hsa-miR-675-3p	hsa-miR-34a-3p

hsa-miR-3679-3p	hsa-miR-4433a-3p	hsa-miR-6829-5p	hsa-miR-4652-3p
hsa-miR-4274	hsa-miR-4716-3p	hsa-miR-7-5p	hsa-miR-4697-5p
hsa-miR-4652-3p	hsa-miR-4734	hsa-miR-885-5p	hsa-miR-489-3p
hsa-miR-4697-5p	hsa-miR-4792	hsa-miR-887-3p	hsa-miR-522-3p
hsa-miR-4716-3p	hsa-miR-5010-3p		hsa-miR-542-5p
hsa-miR-542-5p	hsa-miR-5096		hsa-miR-551b-3p
hsa-miR-551b-3p	hsa-miR-6722-3p		hsa-miR-563
hsa-miR-6722-3p	hsa-miR-6731-3p		hsa-miR-634
hsa-miR-6731-3p	hsa-miR-675-3p		hsa-miR-6753-3p
hsa-miR-6753-3p	hsa-miR-6785-3p		hsa-miR-6757-3p
hsa-miR-6757-3p	hsa-miR-6794-5p		hsa-miR-6759-3p
hsa-miR-6765-3p	hsa-miR-6795-3p		hsa-miR-6765-3p
hsa-miR-6785-3p	hsa-miR-6806-5p		hsa-miR-92b-3p
hsa-miR-6794-5p	hsa-miR-6824-5p		hsa-miR-98-3p
hsa-miR-6795-3p	hsa-miR-6829-5p		
hsa-miR-6806-5p	hsa-miR-7-5p		
hsa-miR-6824-5p	hsa-miR-7845-5p		
hsa-miR-7845-5p	hsa-miR-885-5p		
hsa-miR-92b-3p	hsa-miR-887-3p		
hsa-miR-98-3p			

Supplementary Table legends

Table S10

Combined Reactome, KEGG and GO pathway enrichment analysis of miRNA targets predicted with TarPmiR and Targetscan. Analysis performed with the miRNAs either down- or up-regulated in the acute CAD patients compared to controls, miRNAs either down- or up-regulated in the stable CAD patients compared to the controls, miRNAs either down- or up-regulated in the stable CAD patients compared to the acute CAD patients. These analyses are presented each in separate Excel sheets. Population Total represents total genes in the genome background that are predicted to be affected by miRNAs in general. Pop Hits represents the number of genes involved in the specific pathway. List Total represents the number of genes predicted to be affected by the input miRNA list. Hits represents the total number of genes potentially regulated by the input miRNA list, which are involved in the specific pathway.

Table S11

Combined Reactome, KEGG and GO pathway enrichment analysis of miRNA targets predicted with TarPmiR and miRTarBase. Analysis performed with the miRNAs either down- or up-regulated in the acute CAD patients compared to controls, miRNAs either down- or up-regulated in the stable CAD patients compared to the controls, miRNAs either down- or up-regulated in the stable CAD patients compared to the acute CAD patients. These analyses are presented each in separate Excel sheets. Population Total represents total genes in the genome background that are predicted to be affected by miRNAs in general. Pop Hits represents the number of genes involved in the specific pathway. List Total represents the number of genes predicted to be affected by the input miRNA list. Hits represents the total number of genes potentially regulated by the input miRNA list, which are involved in the specific pathway.
